# MacroH2A histone variants modulate enhancer activity to repress oncogenic programs and cellular reprogramming

**DOI:** 10.1038/s42003-023-04571-1

**Published:** 2023-02-23

**Authors:** Wazim Mohammed Ismail, Amelia Mazzone, Flavia G. Ghiraldini, Jagneet Kaur, Manvir Bains, Amik Munankarmy, Monique S. Bagwell, Stephanie L. Safgren, John Moore-Weiss, Marina Buciuc, Lynzie Shimp, Kelsey A. Leach, Luis F. Duarte, Chandandeep S. Nagi, Saul Carcamo, Chi-Yeh Chung, Dan Hasson, Neda Dadgar, Jian Zhong, Jeong-Heon Lee, Fergus J. Couch, Alexander Revzin, Tamas Ordog, Emily Bernstein, Alexandre Gaspar-Maia

**Affiliations:** 1 Division of Experimental Pathology, Department of Lab Medicine and Pathology, Mayo Clinic, Rochester, MN USA; 2 Center for Individualized Medicine, Epigenomics program, Mayo Clinic, Rochester, MN USA; 3 Department of Oncological Sciences, Icahn School of Medicine at Mount Sinai, New York, NY USA; 4 Tisch Cancer Institute, Icahn School of Medicine at Mount Sinai, New York, NY USA; 5 Department of Pathology and Immunology, Baylor College of Medicine, Houston, TX USA; 6 Graduate School of Biomedical Sciences, Icahn School of Medicine at Mount Sinai, New York, NY USA; 7 Tisch Cancer Institute Bioinformatics for Next Generation Sequencing (BiNGS) Shared Resource Facility, Icahn School of Medicine at Mount Sinai, New York, NY 10029 USA; 8 Department of Physiology and Biomedical Engineering, Mayo Clinic, Rochester, MN USA

**Keywords:** Epigenomics, Data integration

## Abstract

Considerable efforts have been made to characterize active enhancer elements, which can be annotated by accessible chromatin and H3 lysine 27 acetylation (H3K27ac). However, apart from poised enhancers that are observed in early stages of development and putative silencers, the functional significance of *cis*-regulatory elements lacking H3K27ac is poorly understood. Here we show that macroH2A histone variants mark a subset of enhancers in normal and cancer cells, which we coined ‘macro-Bound Enhancers’, that modulate enhancer activity. We find macroH2A variants localized at enhancer elements that are devoid of H3K27ac in a cell type-specific manner, indicating a role for macroH2A at inactive enhancers to maintain cell identity. In following, reactivation of macro-bound enhancers is associated with oncogenic programs in breast cancer and their repressive role is correlated with the activity of macroH2A2 as a negative regulator of BRD4 chromatin occupancy. Finally, through single cell epigenomic profiling of normal mammary stem cells derived from mice, we show that macroH2A deficiency facilitates increased activity of transcription factors associated with stem cell activity.

## Abstract (web summary)

MacroH2A histone variants are shown to mark a subset of enhancers in both normal and cancer cells. In mice, macroH2A deficiency is shown to facilitate increased activity of transcription stem cell-associated transcription factors.

## Introduction

Enhancers are *cis*-regulatory elements found throughout the eukaryotic genome that are bound by transcription factors (TF) and coactivator complexes^1,2^ playing a key modulatory role in gene expression. Chromatin landscape profiling has revealed specific patterns at enhancer regions consisting of a nucleosome-depleted region that is flanked by histones harboring specific post-translational modifications (PTMs) such as H3K4me1 and H3K27ac^3^. This combination of PTMs has been broadly utilized for epigenomic annotation of active enhancers, facilitating systematic discovery and functional understanding of this important class of *cis*-regulatory elements^4^. However, our ability to define an inactive enhancer state has been more elusive due mainly to their association with repressed transcriptional activity. In the absence of H3K27ac (and, in some instances in the presence of H3K27me3), H3K4me1, which in and of itself is largely dispensable for transcription^5^, has been associated with enhancer states that are repressed or poised/primed for activation^6–9^. Recently, H3K27me3-rich genomic regions that negatively regulate gene expression via proximity or looping have been proposed as potential silencers^10^. Together, these data indicate that the regulation of repressed/poised *cis*-regulatory elements (CRE) may be more complex than previously thought. By extension, inactive states may have biological relevance in the context of cellular identity and homeostasis. This is particularly true during oncogenic transformation, where plasticity and reprogramming are altered due in part to genetic or structural disruption of *cis*-regulatory regions^11^ leading to re-activation or hijacking of enhancer elements^12^. Therefore, we hypothesized that dysregulation of the establishment and maintenance of repressive chromatin states in *cis*-regulatory regions could play a role in oncogenic transformation.

Histone variant incorporation into the nucleosome has distinct effects on gene expression, regulating cell specification in both development and cancer^13^. MacroH2A (mH2A) histone variants contain a 30 kDa non-histone domain (macro domain) at their C-termini^14^ and are associated with the inactive X chromosome^15^, various forms of heterochromatin^16^, and inactive genes^17–20^. MacroH2A1 and macroH2A2 isoforms are encoded by two distinct genes (*H2AFY* and *H2AFY2*, respectively), and macroH2A1 is alternatively spliced, resulting in two macroH2A1 isoforms, macroH2A1.1 and macroH2A1.2, that differ by only one exon in the macro domain^21^. The incorporation of mH2A variants into the genome occurs in large chromatin domains^22^ most often marked by the Polycomb-mediated repressive histone modification H3K27me3^19,23^ and in some instances by H2BK12ac^24^. While recent reports have described a dynamic process by which such mH2A domains are negatively defined by exclusion from actively transcribed regions^22^, other regions of the genome are enriched for macroH2A with undefined functions. Previously, mH2A variants have been implicated in the maintenance of cell identity when challenged during somatic cell reprogramming^23,25^, acting as an epigenetic barrier in association with H3K27me3 through co-localization at pluripotency genes in differentiated cells^21^. In cancer, expression of mH2A1 isoforms is somewhat context dependent, with mH2A1.1, but not mH2A1.2, generally acting as a tumor suppressor. Overall expression of mH2A1.1 and mH2A2 is reduced in several tumor types including melanoma, lung, bladder, and breast cancers, as compared to normal tissues and/or early cancer stages^26–30^.

Our current understanding of the role of histone variants at enhancers is limited. In this study, we demonstrate through extensive epigenomic analysis that mH2A variants regulate gene expression through enhancer modulation and identify a specific class of *cis*-regulatory elements, which we termed macro-Bound Enhancers (mBE). We find that mBE play a role in preserving cell identity through cell-specific modulation of transcription, with important implications for cellular reprogramming and activation of oncogenic pathways.

## Results

### Characterization of macro-bound enhancers

**Fig. 1:**
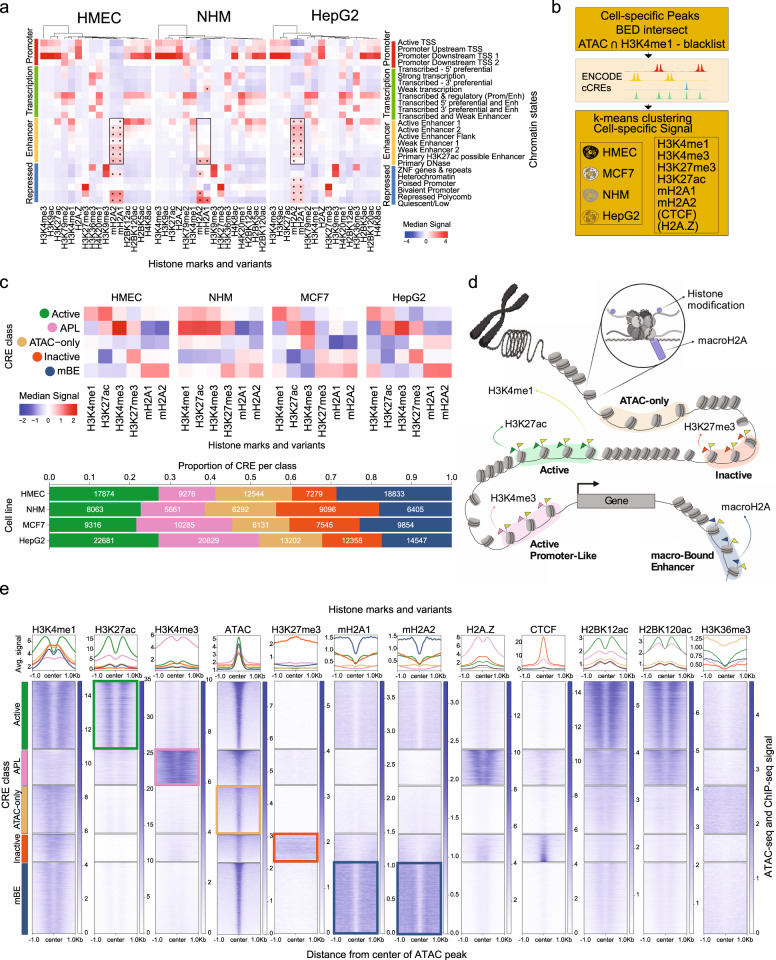
Characterization of macro-Bound Enhancers. **a** Heatmaps showing median signal scores of 14 histone marks from the Roadmap reference human epigenomes (imputed ChIP-Seq signal tracks for E119, E059 and E118) and that of histone variants macroH2A.1 and macroH2A.2 (signal tracks from ChIP-Seq experiments), across all genomic regions in the 25-state chromatin models from Roadmap, built for human mammary epithelial cells (HMEC), normal human melanocytes (NHM) and the human hepatocellular carcinoma cell line (HepG2), respectively. All values are centered and scaled along the column direction. Median signal scores of macroH2A variants in the enhancer states are highlighted using a box. MacroH2A variants enrichments that are statistically significant (*p* < 0.05, one-sided Mann–Whitney *U* test with Bonferroni correction) are marked with an asterisk. **b** Outline of algorithm used to classify cell-specific *cis*-regulatory elements (CRE). **c** Heatmaps showing median Z scores of the log-normalized input-corrected ChIP-seq signal of 6 histone marks/variants used in classifying the CRE sites in each CRE class (top). Bar plot (bottom) shows the proportion of CRE classes in each cell type. **d** Illustration showing each CRE class with corresponding marks (histone modifications or variants). **e** Average signal profile (top) and heatmaps (bottom) of ATAC-seq signal scores, and ChIP-seq signal scores of histone marks and variants around open chromatin regions (defined by ATAC-seq) grouped by the five CRE classes in HMEC.

We performed average signal comparison between ChIP-seq signal of mH2A variants and the 25-state chromatin model from Roadmap Epigenomics^31^ in two different primary cell types (human mammary epithelial cells, HMEC, and normal human melanocytes, NHM) and one cancer cell line (HepG2), which indicated a significantly higher median signal of mH2A at most enhancer states along with repressed polycomb and quiescent states (Fig. 1a). To gain a better understanding of the potential regulatory effects of mH2A deposition at enhancer elements, a pipeline was developed using the ENCODE candidate *cis*-regulatory elements (cCRE) framework (Fig. 1b)^32^. Following identification of cell-type specific *cis*-regulatory elements (CRE), defined as the intersection between cell-type agnostic cCREs from ENCODE and the cell type-specific open chromatin regions analyzed using ATAC-seq (in HMEC, NHM, HepG2, and the breast cancer cell line MCF-7), with the incorporation of H3K4me1 peaks and exclusion of a blacklist of ambiguous genomic regions^32^, *k*-means clustering (*k* = 5) was performed using the ChIP-seq signal from H3K4me1, H3K4me3, H3K27ac, H3K27me3, mH2A1, mH2A2 and when available, H2A.Z and CTCF. Average silhouette scores for all the cell lines were used to determine the optimal number of clusters (Supplementary Fig. 1a). The five clusters identified could be characterized as follows (Fig. 1c–e): Active Enhancers (enriched in H3K27ac), Active Promoter-Like (APL, enriched with H3K4me3), Inactive Enhancers (low H3K27ac), ATAC-only (mostly absent of any other mark used in the classification) and a large subset of enhancers with low H3K27ac and strong mH2A signal, a class of enhancers we coined macro-Bound Enhancers (mBEs), identified in the different cell types between 17 and 29% of all enhancers (Fig. 1c). Genomic annotation enrichment analysis of each of the five classes in the four cell lines revealed that the mBE class was highly represented at intergenic regions (Benjamini-Hochberg corrected *p* < 0.05; Supplementary Fig. 1b) indicating that the regulatory effect of such enhancers goes beyond the known role of macroH2A as regulator of gene expression through promoter and gene-body occupancy^19^.

Since mH2A variants have previously been associated with H3K27me3 around transcription start sites (TSS) and gene bodies, the presence of mH2A at enhancer elements lacking H3K27me3 was unexpected, which was most pronounced in HMEC and HepG2 (Fig. 1c, Supplementary Fig. 1c). The previously reported association of mH2A1.1 with H2BK12ac^24^ was not found at mBEs (Fig. 1e), probably due to the use of a mH2A1 antibody that does not discriminate between the two isoforms. Also, mBEs are devoid of H2A.Z, an H2A variant associated with active TSS and enhancers^33^. Interestingly, the ATAC-only class showed the highest average signal intensity for H3K36me3 in HMEC (Fig. 1e), which could be explained by its predominance at intronic regions of expressed genes (Supplementary Fig. 1b). Not surprisingly, all five classes show similar levels of conservation, but DNA methylation patterns are relatively low in active and mBE enhancers, which could suggest a primed state of mBEs (Supplementary Fig. 1d). Moreover, the overlap between super-enhancer clusters from all four cell types and mBEs is low (Supplementary Fig. 1e). For validation of the enhancer classification, we applied an alternate approach using chromHMM^34^ to build a chromatin state model with 11 histone marks in combination with the mH2A variants, followed by overlap enrichment analysis of the model with the five classes of enhancers. The first five states, which can clearly be identified as enhancer states (marked by H3K4me1), show clear enrichment for each of the 5 classes of enhancers defined by the *k*-means approach (Supplementary Fig. 2a–b).

**Fig. 2:**
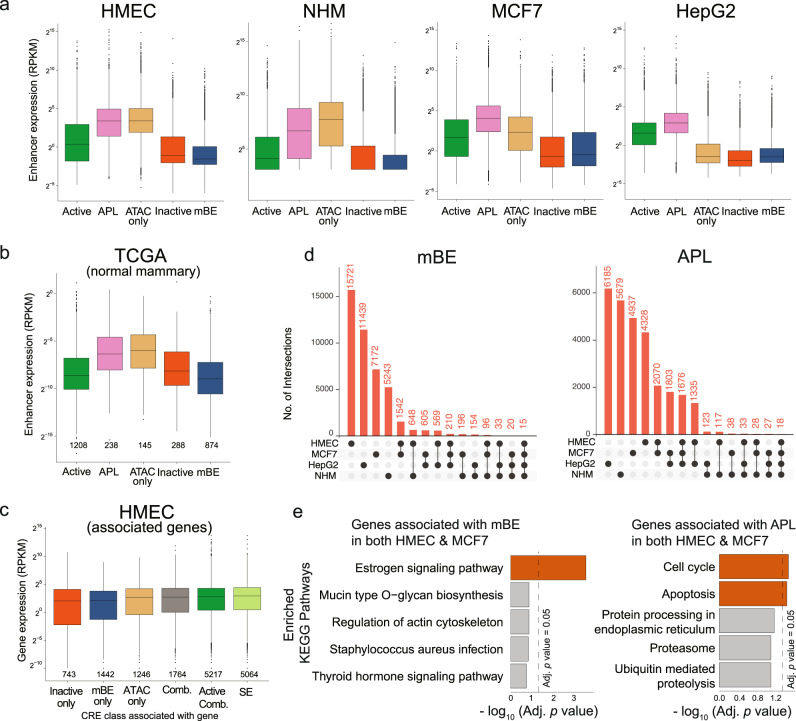
MacroH2A regulates enhancer activity. **a** Expression levels at the CRE grouped by the five classes in human mammary epithelial cells (HMEC), normal human melanocytes (NHM), the breast cancer cell line (MCF7) and the human hepatocellular carcinoma cell line (HepG2) quantified by RNA-seq data from ENCODE reference epigenome for HMEC (total), MCF7 (poly-A) and HepG2 (total), and from Fontanals-Cirera et al.^73^ for NHM (total). The number of datapoints, *n*, equals the number of CRE per class shown in Fig. 1c. **b** Expression levels by RNA-seq from normal breast samples (averaged across 113 samples) from TCGA^35^ at enhancers that overlap the annotated CRE in HMEC. The number of datapoints, *n*, is shown below each box. **c** Expression levels by RNA-seq from Roadmap reference epigenome (E119) at protein-coding genes directly associated with the CRE from HMEC (associations obtained from GeneHancer) and super-enhancers (annotated using LILY). *Inactive only*, *mBE only* and *ATAC only*: genes associated with only Inactive, mBE or ATAC only classes of CRE respectively; *Active Comb*: genes associated with at least one Active CRE and possibly other combination of CRE classes; *Comb*: genes associated with all other combinations of CRE classes; *SE*: genes associated with those CRE identified as super-enhancers. The number of datapoints, *n*, is shown below each box. The expression data is represented as boxplots where the middle line represents the median, the lower and upper edges of the rectangle represent the first and third quartiles and the lower and upper whiskers represent the interquartile range (IQR) × 1.5. Outliers beyond the end of the whiskers are plotted individually. **d** Upset plots showing the intersection of mBE and APL (Active Promoter-Like) CRE loci between the four cell lines. **e** Top 5 most significant KEGG pathways sorted by Benjamini-Hochberg adjusted *p*-value of the minimum hypergeometric (mHG) test performed by Cistrome-GO on genes associated with mBE and APL (Active Promoter-Like) common in HMEC and MCF7.

To validate our pipeline of enhancer mapping, we sought to compare expression of these regulatory elements through publicly available RNA-seq data, as a proxy for their activity^35^. In all four cell types, the highest expressing elements are APL, Active, and the ATAC-only enhancers, which corroborates the idea that most of these latter elements are present in intronic regions of expressed genes since they are also enriched with H3K36me3. The class with the lowest expression detected in the non-malignant cells is mBE both with total RNA (Fig. 2a) and polyA RNA (Supplementary Fig. 2c). The expression in normal mammary tissue^35^ of the enhancer elements as identified in HMECs revealed the same pattern (Fig. 2b). This suggests that the definition of such CREs in mammary epithelial cells is also reflective of enhancer activity in human samples. Moreover, enhancer-gene association of the five classes of CRE confirms that inactive and mBE enhancers are associated with the lowest expressing genes (Fig. 2c). Finally, we queried whether mBE would differ between biosamples. Not surprisingly, the strongest overlap from the four samples was in the APL class associated with TSS of active genes, with mBE and the other enhancer classes having fewer common elements (Supplementary Fig. 2d). Interestingly, the two samples derived from the breast (HMEC and MCF7) showed the greatest overlap in mBE (Fig. 2d, Supplementary Fig. 2e) indicating an important regulatory mechanism common to mammary tissue and breast cancer. Gene ontology (GO) analysis performed on genes ranked by regulatory potential scores of breast-associated APL and mBE, which are calculated based on distance of CREs from TSS of genes, indicated a fundamental difference (Fig. 2e). The mBE-associated genes were highly enriched in the estrogen signaling pathway, while APL-associated genes were mainly associated with cell cycle and apoptosis. These results suggest an important role of mBE as gatekeepers of cellular identity and regulation of developmental specifications.

### MacroH2A is a negative modulator of enhancer activity

**Fig. 3:**
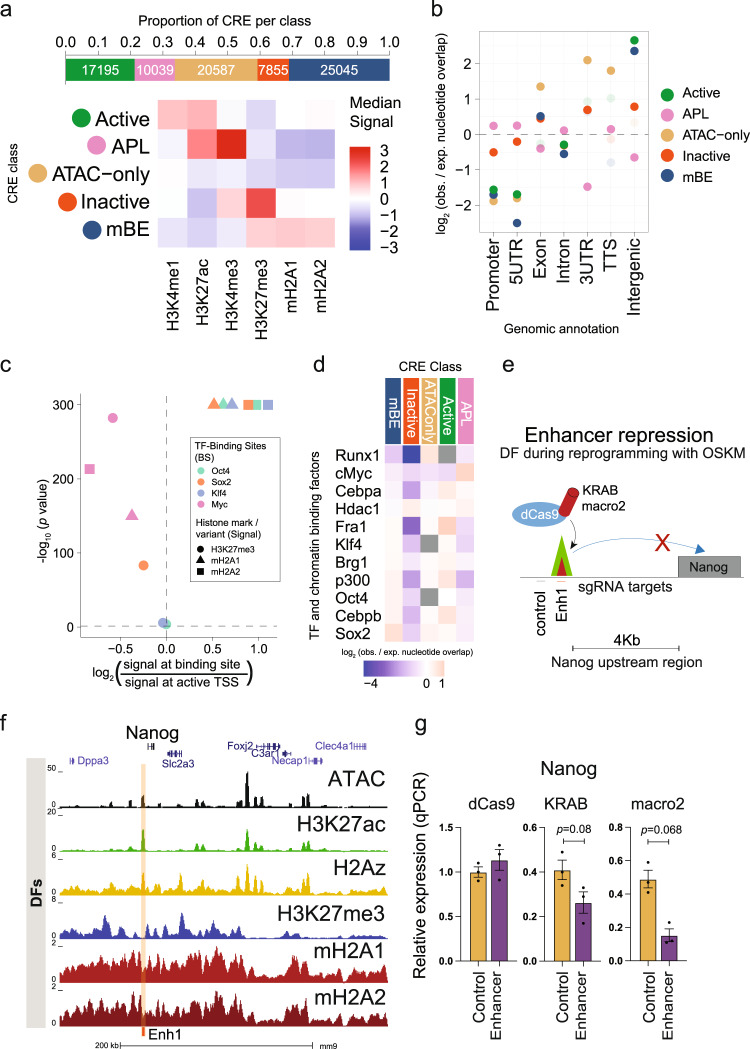
MacroH2A is a negative modulator of enhancer activity. **a** Proportion of CRE peaks in each CRE class (top) and heatmap (bottom) showing median Z scores of the log-normalized input-corrected ChIP-seq signal of the 6 histone marks/variants used in classifying the CRE peaks in each class in dermal fibroblasts (DF). **b** Genomic region enrichment of the CRE peaks in each class as calculated by GAT (enrichments that are statistically significant, Benjamini-Hochberg corrected *p*-value < 0.05, are shown in darker colors, and the rest in lighter colors). **c** Volcano plot showing the enrichment of the signals of macroH2A variants and H3K27me3 at the binding sites (BS) of Oct4, Sox2, Klf4 and cMyc (48 h after OSKM induction, binding sites data collected from Chronis et al.^37^), relative to the signals at active TSS (AT) regions in dermal fibroblasts (Mann–Whitney *U* test). Active TSS (AT) regions are defined as 500 bp up- and downstream of the transcription start sites that have RPKM > 1 measured by RNA-seq experiments on dermal fibroblasts. **d** Enrichment of TF and chromatin binding factors after 48 h of OSKM expression^37^, at CRE sites of DF cells as calculated by GAT, ranked by enrichment in mBE (enrichments that are not statistically significant, Benjamini-Hochberg corrected *p*-value > 0.05, are shown in gray). **e** Schematic of enhancer targeting in DF during reprogramming with OSKM using dCas9-KRAB and dCas9-macro2 and sgRNAs complementary to regions around the enhancer site. **f** UCSC genome browser snapshot of the Klf4 binding site upstream of the Nanog TSS in DF with open chromatin (ATAC-seq), H3K27me3, H3K27ac, H2A.Z, mH2A1 and mH2A2 data. **g** *Nanog* relative expression after 96 h of OSKM infection in DFs with dCas9, dCas9-KRAB or dCas9-macro2 (unpaired two tailed student’s *t*-test, *p* = 0.068) with sgRNAs targeting the enhancer site upstream of Nanog or control. Data are mean with SE (*n* = 3).

In order to address the functional role of mBEs, we performed cellular reprogramming in cells derived from double knockout (dKO) mice^36^ lacking the genes encoding both mH2A variants (*H2afy* and *H2afy2*)^23^. Since dermal fibroblasts (DFs) derived from this model demonstrated that mH2A variants act as a barrier to reprogramming^23^, we hypothesized that mBEs could be enriched at the consensus binding sites of the four iPS reprogramming factors, Oct4 (O), Sox2 (S), Klf4 (K) and Myc (M). Consistent with the results obtained in the human cells, CRE analysis of DFs revealed the highest enrichment of mBEs at intergenic regions (Benjamini-Hochberg corrected *p* = 0.0015), with similar distributions of the five classes (Fig. 3a, b). We next analyzed the enrichment of mH2A variants and H3K27me3 at the OSKM binding sites^37^ 48 hours after OSKM expression in DFs, relative to DF-specific active TSSs. Interestingly, the binding sites of the three pioneering factors (OSK) were significantly enriched in mH2A1 and mH2A2 (*p* < 0.0001), but not H3K27me3 (Fig. 3c, Supplementary Fig. 3a). To further probe mBEs as an epigenetic barrier during reprogramming, ChIP-seq peaks for four TFs highly expressed in fibroblasts (Fra1, Cebpa, Cebpb, and Runx1), three chromatin regulators (Brg1, p300 and Hdac1), and the OSKM factors obtained at 48 hr during iPS reprogramming^37^ were used to calculate the enrichment of binding sites at CRE sites of each class. This analysis revealed significant enrichment of Sox2 and Oct4 binding sites in mBEs (Benjamini-Hochberg corrected *p* = 0.0011), confirming the presence of mH2A variants at the same loci bound by the pioneer factors during the early phases of reprogramming (Fig. 3d).

To functionally address whether mH2A modulates enhancer activity, DFs isolated from mH2A dKO mice were used in iPS reprogramming experiments as described^23^. These cells completely lack mH2A variants, allowing implementation of a strategy to assess the effect of the macro domain at a single locus by means of CRISPR/Cas9-mediated epigenome editing using a dCas9 chimeric protein containing either the macro domain or the repressor KRAB domain (Fig. 3e). First, an embryonic stem cell (ESC) line (NG4) expressing green fluorescent protein (GFP) under the control of the *Nanog* promoter and regulatory enhancer (180 Kb upstream of TSS) was used to establish cell lines with different Cas9 constructs (Supplementary Fig. 3b-d). NG4 cells express green fluorescence under normal ESC growth conditions^38^, and targeting a known regulatory element of *Nanog* (Supplementary Fig. 3e, f) should decrease its expression. SpCas9 was used as a positive control for targeting of the region of interest, dCas9 alone was used as a negative control, and dCas9-KRAB was a positive control for negative modulation of the target enhancer (Supplementary Fig. 3g–i). Targeting was directed to the enhancer, the GFP transgene, and a control region upstream of the enhancer, and GFP expression was determined by fluorescence-activated cell sorting (FACS). The effect of dCas9-macro1.2 and dCas9-macro2 was comparable to dCas9-KRAB, especially at the enhancer and GFP (Supplementary Fig. 3h), indicating that the macro domains promote inhibitory effects both in transcribed regions (as expected) and at enhancer elements. After validating enhancer modulation in ESCs, a similar experiment was then performed to examine the effect on endogenous *Nanog* expression during the process of reprogramming of mH2A dKO DFs (Fig. 3e–g, Supplementary Fig. 3j). Expression of *Nanog* was also reduced upon targeting the enhancer with dCas9-macro2 after four days of iPS reprogramming (Fig. 3g). Thus, the presence of mH2A at enhancers during reprogramming may hinder their activation, explaining in part the role of macroH2A as an epigenetic barrier for reprogramming.

### Reactivation of macro-bound enhancers associates with oncogenic programs

**Fig. 4:**
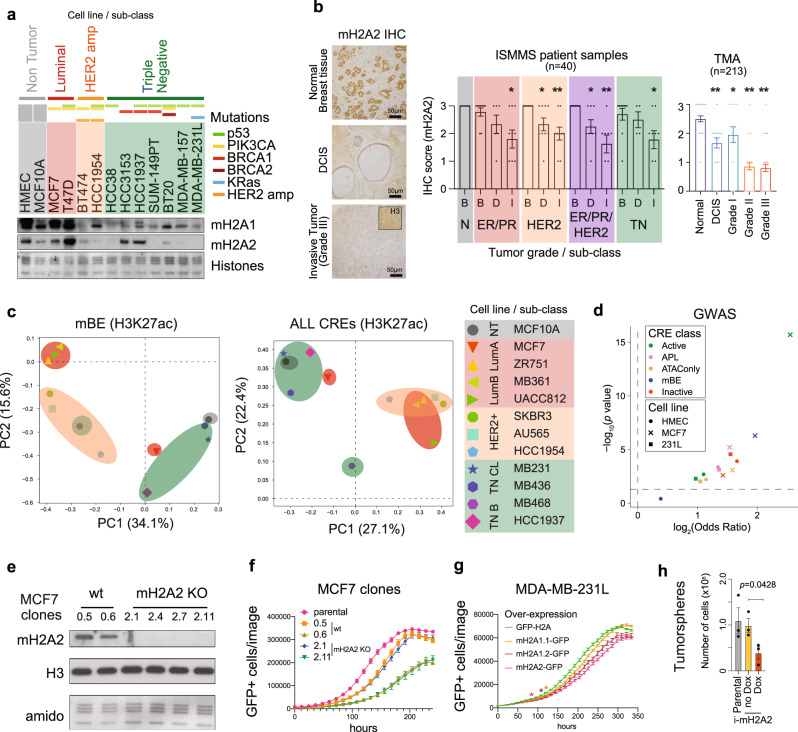
Reactivation of macro-Bound Enhancers associates with oncogenic programs. **a** Immunoblot of chromatin extracts were probed for mH2A1 and mH2A2 across a panel of breast cancer cell lines including the three different major sub-types (Luminal, HER2 positive and triple negative), and non-tumorigenic cells (human mammary epithelial cells, HMEC; and immortalized mammary cells MCF10A). Mutational status defined above. Amido Black of core histones used as loading control. **b** Immunohistochemistry (IHC) from normal breast tissue, ductal carcinoma in situ (DCIS) and grade III invasive tumors for mH2A2. Histone H3 IHC was used as a control (top right). Quantification of mH2A2 scoring for TMA and ISMMS patient samples according to the tumor grade or sub-class (below). B – Benign tissue, D – DCIS, I – Invasive. Column bar represents mean and SE. Unpaired (two tailed) student’s *t*-test *p < 0.05, **p < 0.005. **c** Principal component analysis of H3K27ac ChIP-seq signal in HMEC macro-Bound enhancers (left) and all CREs (right) in 12 breast cancer cell lines from Franco et al.^41^. **d** Volcano plot showing enrichment of breast cancer risk variants from GWAS studies (GWAS *p*-value < 5 × 10^−8^), in each CRE class in HMEC, MCF7 and 231 L cells, enrichment *p*-value and odds ratio calculated using GARFIELD. **e** Immunoblots for mH2A2 from chromatin extracts in MCF7 clones. H3 and histones (amido black) used as loading controls. **f** Proliferation of MCF7 clones (wild type and mH2A2 *KO*) transduced with H2A-GFP and analyzed by number of GFP cells using Incucyte. Data represented are mean with SE (*n* = 3). **g** Proliferation of MDA-MB-231L cells with over-expression of mH2A-GFP constructs (and H2A-GFP as control) determined by the number of GFP positive cells on Incucyte. Data are mean with SD (*n* = 9). Proliferation of cells over-expressing of mH2A1.1-GFP is significantly lower than control starting at 120 hours, mH2A1.2-GFP at 108 hours and mH2A2-GFP at 84 h (Two-way ANOVA with Dunnett’s multiple comparison test, *p < 0.05). **h** Tumorsphere formation assessed by number of cells upon mH2A2-GFP induction (Unpaired two tailed student’s *t*-test, *p* = 0.0428). Data represented are mean with SE (*n* = 3).

Given the above, mBEs may regulate cellular homeostasis and potentially serve as gatekeepers of cell identity by limiting plasticity. In turn, the loss of mH2A during cancer progression could serve as an opportunity for oncogenic gene expression programs by means of enhancer activation. Decreased mH2A expression has been described in a variety of different tumors^13,30^ and has been implicated in processes such as epithelial-mesenchymal transition (EMT) in breast cancer^39^. However, a thorough analysis of loss of mH2A variants has not been performed in mammary carcinoma. Chromatin fractionation of a panel of breast cancer lines revealed several cell lines with a prominent loss of mH2A, particularly mH2A2 (Fig. 4a). The loss of mH2A2 was not limited to a particular sub-type or mutational status, although highly associated with aggressive tumors such as triple negative (TN) and HER2-amplified cancers. We then investigated mH2A2 levels in two cohorts of patient samples (patients from Icahn School of Medicine Mount Sinai (ISMMS) and Breast Cancer Progression tissue microarrays, TMAs) by immunohistochemistry. Similar to the cell lines, mH2A2 was lost in invasive tumors and in tumors with advanced grades (II and III) when compared to ductal carcinoma in situ or grade I tumors, respectively (Fig. 4b).

To evaluate if the loss of mH2A was correlated with reactivation of enhancer elements associated with oncogenic programs, activity of the mammary epithelial CREs (as defined in HMEC) in breast cancer cells lines was analyzed using the ChIP-seq signal for H3K27ac from 12 different breast cancer cell lines^40,41^ including the non-tumorigenic (NT) cell line MCF10A. Principal component analysis (PCA) of the H3K27ac signal at HMEC mBEs suggested a correlation between cancer cell lines from the same cancer subtypes analyzed, i.e., luminal (Lum A and Lum B), HER2-amplified, and TN (including Basal (B) and Claudin Low (CL)), similar to the PCA of H3K27ac signal at all CREs (Fig. 4c). This compelling association indicates that breast cancer sub-types can be identified based on the activity of specific enhancers that were found enriched with mH2A in normal mammary epithelial cells. Further analysis of genome-wide association studies (GWAS)^42^ data showed an enrichment of breast cancer risk variants in mBE from the breast cancer cell line MCF7 (*p* < 0.0001, Fig. 4d).

Since mH2A2 had the most pronounced effect in the reprogramming studies^23^ we modeled the role of mH2A2 using cell lines that represent two extremes in terms of mH2A2 expression (MCF7 and MDA-MB-231L). We depleted mH2A2 in MCF7 cells by CRISPR/Cas9 genome editing using four sgRNAs (Fig. 4e, Supplementary Fig. 4a–d). After screening for efficient sgRNAs (Supplementary Fig. 4b), we isolated and expanded two non-targeting control clones (wt) and two mH2A2 knockout (KO) clones. Analysis of proliferation identified two pairs of clones (control: 0.5 and 0.6 and KO: 2.1 and 2.11) with similar proliferative potential indicating that mH2A2 depletion in MCF7 does not seem to affect their proliferative potential (Fig. 4f). However, over-expression of mH2A variants in MDA-MB-231L leads to decreased proliferative capacity, with mH2A2 over-expression having the most pronounced effect (Fig. 4g). Next, to test oncogenic potential, we used an inducible system to over-express mH2A2 in MDA-MB-231L cells (Supplementary Fig. 4e–g) and performed tumorsphere assays. Induced expression of mH2A2 led to a significant decrease in the tumorsphere growth (Fig. 4h). This data suggests that there is a context-dependent effect of mH2A deposition in the context of breast cancer, and in defining oncogenic programs that are specific to the sub-type of tumor and their distinct transcriptional dependencies.

### mH2A2 is a negative regulator of estrogen targets

**Fig. 5:**
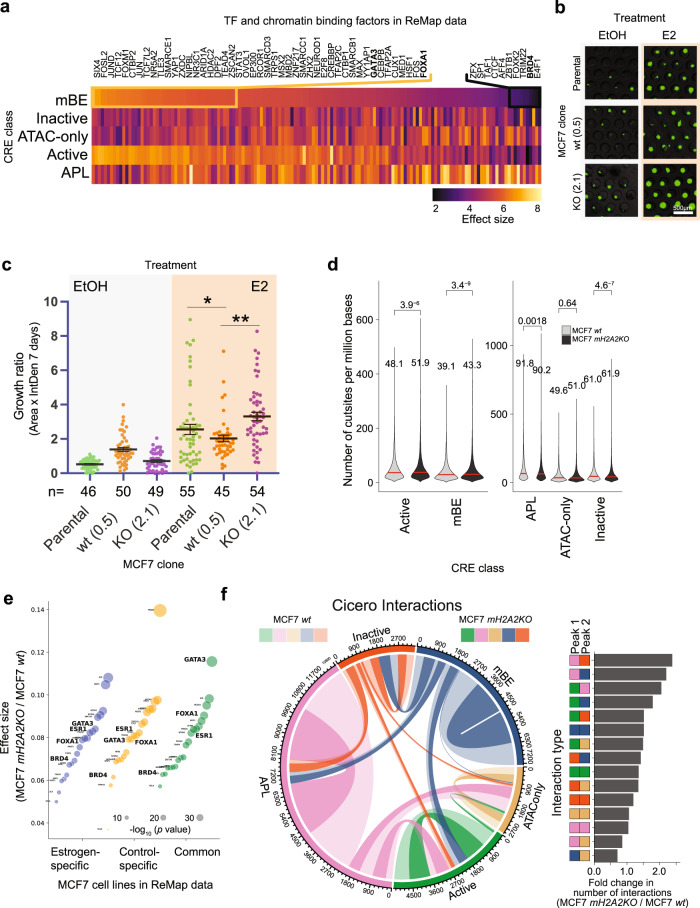
mH2A2 is a negative regulator of estrogen targets. **a** Transcription factors and DNA-binding molecules whose binding sites as defined by ChIP-seq peaks from ReMap data for MCF7 cells are significantly enriched (Benjamini-Hotchberg corrected *p*-value < 0.05) at CRE sites from each CRE class of MCF7 cells (enrichment statistics computed using ReMapEnrich). Effect size is defined as the log (base 10) ratio between the observed and expected number of overlaps. Molecules are ranked by enrichment in mBE. **b** Representative images of MCF7 spheroids. Scale bar, 500 µm. **c** Growth ratio of MCF7 3D spheroids after treatment with EtOH and E2 (Estradiol) in microwells after 7 days. Scatter plot of area factored with GFP Intensity Density in individual spheroids. Horizontal bars signify mean values +/− SE (left). Unpaired (two-tailed) student’s *t*-test *p < 0.05, **p < 0.005. **d** Violin plots showing the number of cut sites (per million bases) overlapping the five classes of CRE in MCF7 *wt* and *mH2A2KO* clones. The number of datapoints, *n*, equals the number of CRE per class shown in Fig. 1c. **e** Cohen’s effect size distributions of TF binding sites in *mH2A2KO* cells compared to *wt* MCF7 cells, grouped by binding sites found in estrogen- or control-specific cell lines (or both). **f** Circos chord diagram (left) showing the distribution of interactions between CREs that belong to each pair of CRE classes in MCF7 *wt* and *mH2A2KO* cells. Interactions were predicted using Cicero on scATAC-seq data using a threshold of co-accessibility score > 0.1. The width of each chord represents the number of interactions between CRE of each pair of classes. Lighter colors are used to represent *wt* counts and darker colors to represent *mH2A2KO* counts. The fold change of number of interactions per interaction type in *mH2A2KO* over those in *wt* are shown as a bar plot (right).

To understand the context-dependent effect of mH2A regulation at enhancers, we defined its potential role in MCF7, which is an estrogen receptor (ER) responsive cell line with a well-defined enhancer network^43^. We first performed in silico analysis of TF and chromatin regulator binding to DNA in MCF7 cells to understand mBE-related regulation. Since this is a commonly used model system, several publicly available ChIP-seq datasets are available to compare the binding of different factors and the annotated enhancers. We applied enrichment analysis of binding sites in MCF7 cells that exists in both the ReMAP database^44^ and our annotated enhancer sub-groups (Fig. 5a). As expected, Active Promoters were most enriched in DNA-binding proteins. Despite mBEs being repressive and mostly depleted of significant binding events, we identified preferential mBE binding of TFs associated with ER activation, namely, GATA3 and FOXA1, in MCF7 cells. A potential explanation is that mBEs help to maintain enhancer stability and define TF programs in a more robust and predictable way to generate and preserve cellular homeostasis and prevent unwanted cellular heterogeneity. Therefore, we hypothesized that mBEs could maintain ER-responsive enhancer elements inactive and in turn serve as a gatekeeper of the MCF7 enhancer network. This notion is also supported by the finding that MCF7 cells have mH2A1 and mH2A2 levels similar to non-tumorigenic cells (Fig. 4a). It follows from this hypothesis that disruption of mBE with the loss of mH2A2 would render the ER-dependent transcriptional program even more accentuated. To analyze ER response^45^, we monitored 3D spheroids of MCF7 clones (parental cells, clone 0.5 as wt and clone 2.1 as mH2A2 KO) in the presence or absence of Estradiol (E2) using microfluidic devices and printed microwells. After 7 days, mH2A2 KO MCF7 cells showed an increased response to E2 when compared with parental or control MCF7 cells, as measured by the tumorsphere assay (Fig. 5b, c). Such results suggest that the loss of the regulatory mBEs led to an overall increase of available ER regulatory regions.

To gain insights into the ER-responsive elements involved in E2 stimulation regulated by mH2A2, we mapped open chromatin regions at single-cell resolution in MCF7 cells after treatment with E2 for five days using single cell ATAC-seq (scATAC-seq). We aimed to understand the impact of mH2A2 loss on ER (ESR1) motif accessibility. We obtained high-quality single-cell profiles derived from a MCF7 wild type (control) and from a mH2A2 KO MCF7 clone (two replicates each) (Supplementary Fig. 5a–c). The control and mH2A2 KO scATAC-seq profiles were analyzed using UMAP projections and graph-based clustering (Supplementary Fig. 5d) using the MCF7 specific *cis*-regulatory elements that we characterized (Fig. 1c) as the input peak set. This analysis approach allowed us to examine the changes in chromatin accessibility through the lens of MCF7-specific regulatory elements. Even though we could observe an outlier cluster in the mH2A2 KO MCF7 clone, we confirmed from quality control (QC) statistics that the quality of cells alone do not justify the outlier (Supplementary Fig. 5b). Globally, depletion of mH2A2 allowed for the enrichment of cut sites overlapping with mBEs (*p* < 0.0001), whereas the enrichment was less pronounced in Active Enhancers (*p* < 0.0001, Fig. 5d). This increased availability of open chromatin regions in the absence of mH2A2 was specific to enhancers, as the promoter regions showed a significantly decreased transposase accessibility in mH2A2 KO cells vs. wild-type cells (*p* = 0.0018, Mann–Whitney *U* test). Binding events from the ReMAP database obtained in MCF7 cells with and without E2 exposure were then analyzed and compared to open chromatin regions (cut sites) in the two MCF7 clones (Fig. 5e). ESR1, GATA3, FOXA1, and BRD4 were among the TF and chromatin regulators that were significantly enriched in the absence of mH2A2. To assess the effect of mH2A2 KO on higher-order chromatin interactions, we used Cicero to calculate co-accessibility scores based on correlated open chromatin sites in these cells. Overall, we observed a significant increase in the number of interactions per peak (*p* = 0.00012, Supplementary Fig. 5e, f). On comparing the number of interactions (those with co-accessibility scores > 0.1) grouped by interaction-types based on the CRE class their end points belonged to, we observed that most interactions involve APL (active promoter-like) elements with all other CRE classes (Fig. 5f). When we compare the changes in number of interactions by interaction-type between wild type and mH2A2 KO, we noted that the highest increase is between APL and Inactive elements followed by interactions between APL and mBE. Four out of the top eight interaction-types by fold-change involve mBE. Such gained interactions between promoters and mBE are associated with genes with stem cell signatures, such as SOX9 and HES1 (Supplementary Fig. 5g, h, Supplementary Data 1).

### mH2A2 is a negative regulator of BRD4

**Fig. 6:**
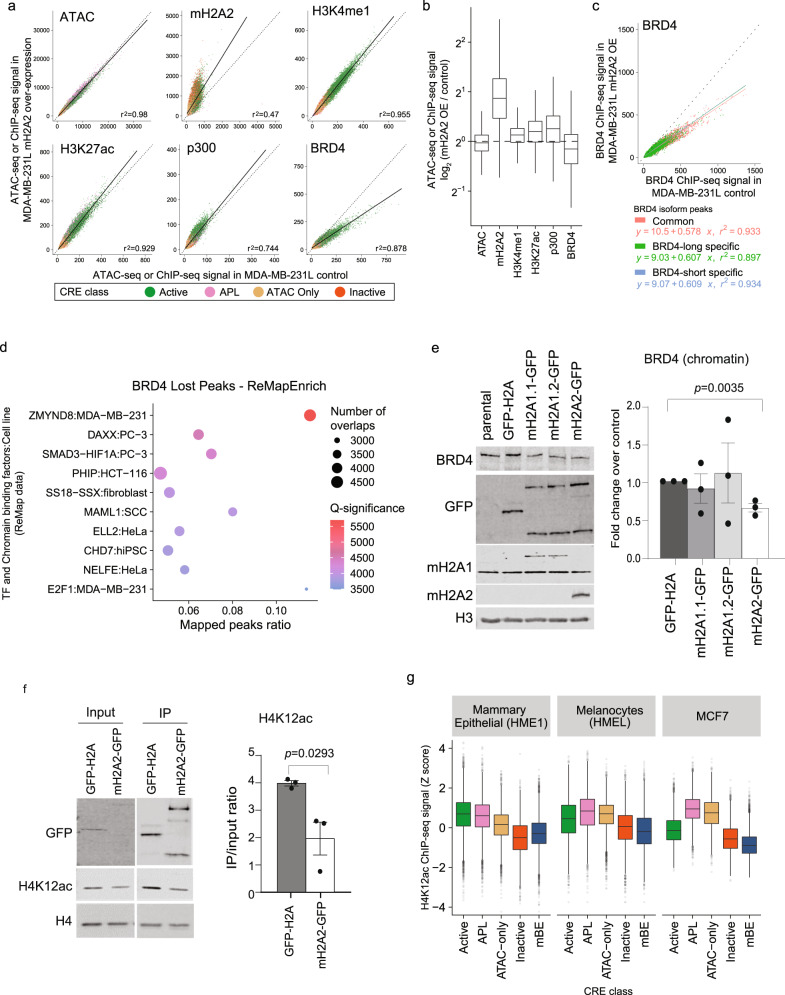
mH2A2 is a negative regulator of BRD4. **a** Scatter plot of input-corrected ChIP-seq signals for mH2A2, H3K4me1, H3K27ac, p300 and BRD4, and ATAC-seq after over-expression of mH2A2 against control over-expression (GFP-H2A) at all CRE (Active, *n* = 16341; APL, 5194; ATAC-only, 13921; Inactive, 14875 peaks) in MDA-MB-231L cells. APL, Active Promoter-Like. The linear regression line (solid line) is shown along with the line y = x for reference. **b** Boxplots showing the log_2_ fold change of input-corrected ChIP-seq signals of each histone mark, histone variant or DNA binding protein in **a** in mH2A2 over-expression over control over-expression. **c** Scatter plot of input-corrected ChIP-seq signals for BRD4 after over-expression of mH2A2 against control over-expression (GFP-H2A) at CUT&RUN peaks that are specific to short (*n* = 2151) and long (*n* = 27684) isoforms of BRD4, and those that were common to both (*n* = 21836) (CUT&RUN peaks from Wu et al.^46^) in MDA-MB-231L cells. The linear regression lines for each set of peaks are shown as solid lines along with the line y = x for reference. **d** Top 10 significantly enriched DNA-binding molecules whose binding sites as defined by ChIP-seq peaks from ReMap are enriched in peaks that lost BRD4 (fold change < 0.5) on over-expression of mH2A2 in MDA-MB-231L cells (enrichment statistics computed using ReMapEnrich). **e** Immunoblots from chromatin extracts in MDA-MB-231L cells with over-expression of mH2A1-GFP and mH2A2-GFP constructs (and H2A-GFP as control) probed for BRD4, GFP and histone H3 (loading control). Fold change quantification over control (H2A-GFP) after H3 normalization (right). Data represented are mean with SE, *n* = 3 (*t*-test). **f** Representative immunoblots from chromatin extracts in MDA-MB-231L cells with over-expression of mH2A2-GFP and H2A-GFP after MNase immunoprecipitation. Extracts were probed for GFP, H4 and H4K12ac. Quantification of IP over input ration (right). Data represented are mean with SE, *n* = 3 (*t*-test). **g** Boxplots showing the Z scores of the log-normalized input-corrected ChIP-seq signal of histone mark H4K12ac in mammary epithelial cells, melanocytes and MCF7 breast cancer cells. The number of datapoints, *n*, equals the number of CRE per class shown in Fig. 1c. In all boxplots, the middle line represents the median, the lower and upper edges of the rectangle represent the first and third quartiles and the lower and upper whiskers represent the interquartile range (IQR) × 1.5.

To gain insights into the global effect of the mH2A2 on enhancer regulation in a context where this histone variant is depleted, we turned to MDA-MB-231L that lacks mH2A2 and expresses mH2A1 at a reduced level (Fig. 4a). Since over-expression of mH2A led to a decrease in proliferation and tumorsphere formation, we sought to understand if enhancers were specifically affected by the ectopic expression of mH2A2. By performing ChIP-seq analysis of H3K4me1, H3K27ac, BRD4 and p300, together with ATAC-seq, we observed that BRD4 binding at CREs was reduced when mH2A2 was over-expressed (Fig. 6a, b, Supplementary Fig. 6a, b) suggesting that mH2A2 deposition around specific enhancers may inhibit BRD4 binding. BRD4 function has been widely associated with the activation of transcription through its association with promoters and enhancers, but its long and short isoforms have different roles^46^. By overlapping the specific binding sites of the different isoforms in MDA-MB-231^46^, we observed that the over-expression of mH2A2 did not affect BRD4 long and BRD4 short isoforms differently (Fig. 6c). We next analyzed the enrichment for peaks that lost BRD4 (Supplementary Fig. 6b) in binding sites of all DNA-binding molecules in the ReMAP dataset. The top enrichment for BRD4 lost peaks upon over-expression of mH2A2 was the zinc finger MYND-type containing protein 8 (ZMYND8) binding sites in MDA-MB-231 cells^47^ (Fig. 6d, Supplementary Fig. 6c). Notably, the expression of ZMYND8 did not change upon ectopic expression of mH2A variants (Supplementary Fig. 6d). ZMYND8 is a multidomain epigenetic reader, containing a BRD-PHD-PWWP cassette with a zinc finger MYND domain^48^, that interacts with HIF-1α and HIF-2α and enhances elongation of the global HIF-induced oncogenic genes by increasing recruitment of BRD4. Interestingly, this multidomain reader also interacts with H2AFY in a BRD domain-dependent manner^48^, suggesting complex interactions within enhancer elements that are dependent on multivalent binding effectors that mBE may help define, in a cell and context-specific manner. To validate that the loss of BRD4 is associated with enhancers, we chose a distal mBE for Cas9 targeting using four sgRNAs to quantify the expression levels of the neighboring genes RBKS and FOSL2. We found that genomic deletion of the enhancer does negatively affect the expression of RBKS but not significantly FOSL2 (Supplementary Fig. 6e).

We hypothesized that mBEs could negatively regulate enhancer activation through the eviction of BRD4 from chromatin. Using chromatin extracts from cells with ectopic expression of the three mH2A isoforms tagged with GFP, and GFP-H2A as a control in MDA-MB-231L, we observed the loss of BRD4 in chromatin (Fig. 6e) but not in whole cell extracts or by qPCR (Supplementary Fig. 6f, g). The most robust effect was response to mH2A2 over-expression. Interestingly, BRD4 binding is also negatively associated with mBEs in MCF7 cells (Fig. 5a). Re-expression of mH2A2 in the mH2A2 KO MCF7 cell clones also showed the loss of BRD4 from chromatin but not whole cell extracts (Supplementary Fig. 6h, i). Since BRD4 is a reader of H4 acetylated residues with high affinity for H4K12ac in both embryonic stem cells^49^ and breast cancer cells^50^, we queried if mH2A2 nucleosomes are devoid of H4K12ac. Immunoprecipitation studies show that mH2A2-GFP tagged nucleosomes show less H4K12ac when compared to H2A-GFP tagged nucleosomes (Fig. 6f). Moreover, genomic analysis of H4K12ac ChIP-seq data across cell types where the enhancer classes have been defined (human mammary epithelial^51^, melanocytes^52^, and MCF7^50,53^), show that mBEs are depleted of H4K12ac (Fig. 6g).

### macroH2A deficiency in MaSC reveals a stem-like signature associated with increased TF activity

**Fig. 7:**
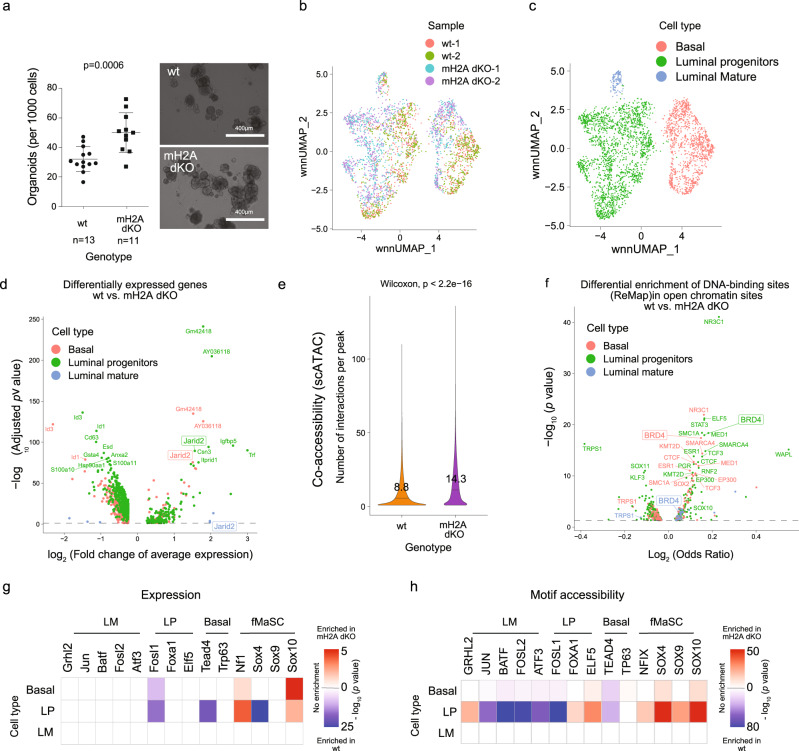
Loss of mH2A histone variants in MaSC reveals a proto-oncogenic signature associated with increased Sox10 activity. **a** Number of organoids per 100 cells plated from wt and mH2A dKO mouse mammary epithelial cells after 7 days in culture. Data are mean with SD (*p* = 0.0006, unpaired *t*-test). **b** Weighted nearest neighbor (WNN) UMAP plot—combining single cell gene expression and single cell ATAC-seq signals, of mammary stem cells from wild type (*n* = 1558) and mH2A double KO (*n* = 1558) (two replicates each). **c** WNN UMAP plot showing three cell types (Basal, *n* = 1113; Luminal progenitors, *n* = 1891; Luminal Mature, *n* = 112) identified after clustering by graph-based clustering of the combined scRNA-seq and scATAC-seq signals and identifying the cell-types using markers. **d** Volcano plot showing differentially expressed genes in each cell type tested (separately) using Mann–Whitney *U* test with Bonferroni correction. **e** Violin plot showing the number of interactions per open chromatin region in wt (*n* = 199052) and mH2AdKO (*n* = 217727) with interactions (co-accessibility score > 0.1) predicted from the scATAC-seq data using Cicero. Mean and *p*-values shown were obtained by comparing means using Mann–Whitney *U* test. **f** Volcano plot showing the differential enrichment of ChIP-seq peaks of DNA binding proteins from ReMap in open chromatin regions in each cell type tested using Fisher’s exact test. Open chromatin regions per cell type were chosen as peaks that had a non-zero number of cut-sites in at least 10% of the cells of that cell type. **g** Heatmap showing the enrichment of gene expression of transcription factors in each cell-type (enrichment represented by −log_10_ (*p*-value) from Mann–Whitney *U* test with Bonferroni correction on gene expression). **h** Heatmap showing the motif enrichment of transcription factors in each cell-type (enrichment represented by -log_10_ (*p*-value) from Mann–Whitney *U* test with Bonferroni correction on chromVAR motif enrichment scores). LP luminal progenitors, ER estrogen, fMaSC fetal mammary stem cells, LM luminal mature.

Next, we investigated the potential role of mH2A variants in restraining mammary epithelial cells from oncogenic programs. To do so, we investigated mammary gland development in 11-week-old virgin female 129 S wild type (WT, *H2afy*+*/+, H2Afy2*+*/+)* and mH2A dKO (*H2afy−/−, H2Afy2−/−)*^36^ mice. By analyzing 24 dKO and 15 WT mice, we found that while in WT mice, the mammary gland had properly gone through ductal morphogenesis that completely filled the fat pad, the dKO mice displayed either a “short” phenotype (decrease in the filling of the fat pad), a “long” phenotype (normal filling of the fat pad with an altered luminal cell ratio), or a “normal” (WT-like) phenotype (Supplementary Fig. 7a–d). While the ratio of basal to luminal cells remained largely the same in all mice, the long phenotype exhibited an increase in the luminal progenitor population (Supplementary Fig. 7e, f). This suggests that in some macroH2A dKO mice, the mammary epithelial cells present a more ‘stem-like’ state. To investigate this stem cell potential, we isolated mammary epithelial cells (MECs) from WT and dKO mammary glands and plated them in mammary stem cell media to form organoids in 3D cultures that enrich for mammary stem cells (MaSC)^54^. Importantly, by isolating MaSC from mammary glands across the range of phenotypes, the overall ability of the mH2A dKO cells to form organoids was significantly increased (Fig. 7a), akin to the effect of mH2A in the reprogramming studies. Next, we confirmed that the MaSC from the “long” phenotype maintained a similar potential (Supplementary Fig. 7g).

Given that MaSCs represent a dynamic group of adult stem cells that are responsible for generating different cell populations that constitute the adult mammary gland, we next used single cell profiling of the organoids using the Multiome platform that allows us to analyze the open chromatin (by scATAC-seq) and expression (by scRNA-seq) patterns from the same nuclei and compare the WT versus mH2A dKO. Because of the variability observed in mH2A dKO mice we chose to analyze the MaSC from a pair of littermates of the long phenotype (D92 wt and D93 mH2A dKO; Supplementary Fig. 7c). Using a weighted nearest neighbor approach to integrate and use both modalities for clustering, we identified three different sub-populations in the MaSC after 7 days of 3D culture (Fig. 7b, c, Supplementary Fig. 8a); basal (marked by Krt14 and Axl), luminal progenitors (marked by Krt18 and Elf5), and luminal mature, or estrogen-expressing cells (marked with Pgr and Esr1) (Supplementary Fig. 8b–d). In agreement with the FACS data, we observed an increase in luminal progenitor cells in the dKO organoids (Supplementary Fig. 8b). We then analyzed the expression and open chromatin patterns between the two genotypes. Most of the differentially expressed genes are in luminal progenitor cells, which is consistent with the enrichment of this subtype in dKO (Fig. 7d, Supplementary Data 2). Open chromatin interactions as measured by co-accessibility between different loci predicted by Cicero, shows a significantly higher number of chromatin interactions per peak (*p* < 0.0001, Fig. 7e). In addition, comparing the enrichment of binding sites of DNA binding proteins from ReMap dataset at WT and dKO open chromatin regions indicates a strong increase in accessibility of BRD4 binding sites across all lineages, suggesting that the loss of mH2A variants increases Brd4 activity (Fig. 7f), which was also observed when considering only enhancer binding, by removing binding sites in TSS regions (Supplementary Fig. 8e).

Finally, we hypothesized that the absence of mH2A would increase enhancer activity specifically allowing for stem cell programs to be activated. Recent epigenetic and transcriptomic profiling of the mammary gland implicated the SOX family of TFs in increasing cell plasticity in the mammary stem cell state^53^ and we probed expression and activity of a panel of known TFs in defining mammary gland differentiation. Interestingly, some fetal mammary stem cell (fMaSC) markers such as SOX10 are upregulated in basal and luminal progenitors (Supplementary Data 2), and TF activity inferred with motif enrichment in open chromatin regions, as predicted by chromVAR, was highly enriched for SOX10, SOX9 and SOX4 motifs in mH2A dKO, especially in luminal progenitor cells (Fig. 7g, h). Such TF activity has been identified in previous studies in fMaSC and human tumors^55^, suggesting that mH2A deficiency in the mammary gland indeed contributes to epigenetic plasticity, which could potentially lead to activation of oncogenic programs, such as those driven by the TF Sox10^55^.

## Discussion

To understand the process by which tumors hijack regulatory elements to their benefit, we must define such regulatory elements in homeostasis and their potential role in defining cell identity and cellular heterogeneity. A better characterization of enhancer activity in cancer may reveal unknown transcriptional dependencies, specific pathways and altered enhancer states that may be valuable in designing new therapeutic approaches. In addition, the role of histone variants at enhancer elements remains poorly defined. Here we characterize a specific class of regulatory elements that lack H3K27ac and are enriched with the mH2A histone variants. These macro-bound enhancers (mBEs) are associated with transcriptional modules that reflect cell-specific functions. The role of mH2A in modulating enhancer activity reveals a specific role for mH2A variants, beyond the described associations with the histone marks H3K27me3 and H2BK12ac. While our epigenomic analysis points out the enrichment of both mH2A1 and mH2A2 at mBE, our data suggest a specific role for mH2A2 as a repressor, consistent with its role as a barrier to iPS reprogramming^23^. Other studies have shown mH2A1 isoforms to be implicated in regulation of enhancers but were limited to one cellular model or a particular mH2A isoform, e.g., mH2A1.2 in skeletal muscle C2C12^56^ and mH2A1.1 in MDA-MB-231 cells^57^. Even if our focus was on the functional role of mH2A2 at enhancers, the enrichment of both variants is important to define mBEs and the effect we observed from the dKO MaSC suggests that mBEs function as a fine-tuning mechanism rather than indispensable regulators of normal development. Importantly, despite the variability of phenotypes observed in the mammary glands, which could be due to mouse-to-mouse variability in response to extrinsic cues such as hormones, we showed that the ability to form MaSC is increased with depletion of mH2A variants regardless of such variability. These studies using MaSC indicate that loss of mH2A unleashes cellular plasticity, which could contribute to diseases such as cancer.

The biological parallels between reprogramming and cancer transformation led us to inquire whether mBEs could have a role in oncogenic activation. Here we demonstrate that mBEs identified in human mammary epithelial cells are associated with specific breast cancer subtypes when reactivated. In fact, the specific oncogenic programs that characterize various sub-types of breast cancer are in part encoded in such mBEs and regulate specific transcriptional dependencies. For example, the loss of mH2A2 in MCF7 cells leads to a more robust response to estrogen. This may occur in part due to enhancer deregulation which allows cancers to gain access to transcriptional programs that were otherwise inaccessible. On the other hand, gain of function of mH2A2 led to loss of chromatin bound BRD4, a bromodomain-containing reader of histone acetylation that binds active enhancers and promoters, with concomitant loss of H4K12ac and a strong association with targets of ZMYND8. Mass spectrometry studies have shown that bromodomain and extraterminal domain (BET) proteins do in fact interact with mH2A histone variants^58^, and the multi-reader ZMYND8 has also been shown to interact with mH2A through its bromodomain^48^. At this point, it remains to be determined if the relationship between mBEs and tumor type-specific oncogenic programs is causal or a mere consequence of the loss of different mH2A isoforms in tumors. However, even the latter could be significant if these changes could be linked to variations in drug response or metastatic potential, and thus mH2A variants could be used as a biomarker.

To better understand how mH2A could function as a repressor of enhancer activity, we showed that the macro domain of mH2A isoforms is sufficient to promote inactivation of enhancers by making use of a chimeric dCas9-macro system to target a specific enhancer in ESCs and DFs during reprogramming. Such repression was comparable to the well characterized dCas9-KRAB system. These experiments are not direct surrogates of mH2A function in chromatin, because they do not incorporate into the nucleosome as the histone variants do but provide insights into the functions of the domains that are required for mH2A-mediated repression. Moreover, it adds an important tool to the growing set of repressive systems available for experimental modulation of enhancers, alongside the repressive KRAB and DNMT domains or EZH2^59^, which have been shown to work in a context-dependent manner.

## Methods

### Cell culture

Normal Human Melanocytes (NHM) were cultured in Dermal Cell Basal Medium (ATCC) with the addition of 5 µg/ml Insulin, 50 µg/ml Ascorbic Acid, 6 mM L-Glutamine, 1.0 µM Epinephrine, 1.5 mM Calcium Chloride, Peptide Growth Factor and M8 Supplement. Dermal fibroblasts (DFs) were isolated from neonatal mice and iPS reprogramming was performed as described^1^. MCF-7, DFs, and MDA-MB-231L cells were grown in DMEM (Gibco) with 4.5 g/l D-glucose, 110 mg/l sodium pyruvate, 10% FBS and 1% Penicillin/Streptomycin (Hyclone). HMEC cells were grown in complete Mammary Epithelial Cell Growth Media. For estradiol (E2) treatment, we transduced MCF-7 clones (parental cells, clone 0.5 as *wt* and clone 2.1 as *mH2A2 KO*) with H2A-GFP (for imaging quantification purposes) and grew them in 2D conditions with EtOH or 17β-estradiol (E2; used as an ER agonist) for 5 days in modified DMEM without phenol-red (Hyclone) with 4.5 g/l D-glucose, 4.0 L-glutamine, 10% charcoal-dextran–stripped FBS, 1% Penicillin/Streptomycin and 1 nM of 17-β-estradiol or EtOH. Cells were then plated as 3D spheroids for another 7 days in the presence or absence of E2, using microfluidic devices and printed microwells that allow for accurate growth quantification using GFP fluorescence, as described below. For growth curves, 1000 cells stably expressing H2A-GFP were plated in each well of a 96-well plate and their growth was followed for 14 days in Incucyte (Sartorius), with acquisition every 12 h.

### Constructs

The four transcription factors (Oct4, Sox2, Klf4, and Myc) used for iPS reprogramming are encoded in a polycystronic lentiviral vector (Stemcca, kindly provided by Gustavo Mostoslavsky, Boston University). LentiCRISPR v2 (Addgene plasmid # 52961) and lentiCas9-Blast (Addgene Plasmid #52962) were a gift from Feng Zhang^60^. To generate CRISPR clones in MCF7 cells, sgRNAs targeting H2AFY2 were selected using CRISPR Design Tool (http://crispr.mit.edu) and cloned using BsmBI enzyme (NEB). SgRNAs targeting the *H2AFY2* locus were: 1- GTTCAGCTAGGGCAGGTGTC, 2- GTTCAAGTACCGGATCAGCG, 3- GGCGGCAGTCATTGAGTACC. Human H2A and macroH2A isoforms were GFP-tagged and subcloned into pLKO.1 plasmid for lentiviral production. Tagged macroH2A2 isoform was subcloned into lentiviral vectors pLVX (Clontech) for dox-inducible expression together with pLVX-Tet3G-Neo. pHAGE EF1α dCas9-KRAB was a gift from Rene Maehr & Scot Wolfe (Addgene plasmid # 50919). pHAGE-EF1-dCas9 plasmids were generated by cloning macro domains from mH2A1.1, mH2A1.2, and mH2A2 in replacement of the KRAB domain. SgRNAs targeting the Nanog-GFP locus: Control (GACGGGTCTCCAGTAGTTCG), Enhancer (GACAGGAATGGGGGTTGGGGA), GFP-1 (GGGCGAGGAGCTGTTCACCG), GFP-2 (GTAGGTCAGGGTGGTCACGA). sgRNAs targeting the enhancer loci were: E1 (GCTAGCCTCCGTACCTCAGCA), E2 (GCTTGAGATCGTCAACCTGA), E3 (GGCTTAAAACGATAGCCATA), and E4 (GCGTCTTATTCCTGACGGTCC). SgRNAs were cloned using BbsI enzyme (NEB) into pLKO-GFP-H2A or pLKO-mCh-H2A. The packaging plasmids for the preparation of lentiviral particles were psPAX2 and pMD2G.

### Lentiviral production

Transgenic cell lines with stable integration of constructs were generated by lentiviral transduction followed by selection in 2 μg/ml puromycin (Millipore) or 5 μg/ml blasticidin (InvivoGen) or 400 μg/ml neomycin (Millipore). Lentiviral particles used in this study were produced in house as previously described^22^. Briefly, lentiviral vectors containing constructs of interest were transfected into 293 T cells together with packaging plasmids using calcium phosphate methods. Media containing lentiviral particles was collected at 36, 48, and 60 h post-transfection, filtered and concentrated by ultracentrifugation at 25,000 rpm for 90 min.

### MCF7 CRISPR/Cas9 mH2A2 knockout clones

LentiCRISPR v2 (Addgene plasmid # 52961) was used to generate CRISPR clones in MCF7 cells with sgRNAs targeting H2AFY2. After transduction, puromycin selection was performed and 1000 cells were plated in a 10 cm dish. After 3 or 4 weeks, clones were identified and selected from the empty vector control or H2AFY2 targeting. Following expansion, clones were identified by western blot.

### CRISPR/Cas9 knockout of enhancer regions

CRISPR–Cas9-based techniques were used to disrupt specific enhancers in the genome of MDA-MB-231L cells and to examine by RTqPCR the consequent changes in the expression of genes regulated by such enhancers. A pair of sgRNAs targeting the region of interest were designed and cloned into the pLKO.1-GFP vector as described above. After production of lentiviral particles, each sgRNA was introduced by lentiviral transduction in MDA-MB-231L cells stably transduced with Cas9. Three days after transduction, the cells were collected for RNA extraction and RTqPCR. The primers used for this purpose are listed in Supplementary Table 1.

### Flow cytometry analysis

NG4, MDA-MB-231, and mouse mammary cells were trypsinized, washed in PBS, strained with a 100 µm filter, and resuspended at 1 × 10^7^ cells/ml in FACS buffer (DPBS and 2% BSA) at 4 °C. GFP and mCherry fluorescence or secondary antibody fluorescence was analyzed by FACS on a LSRII machine and data was analyzed with FlowJo.

### Microfluidic devices and tumorspheres

Development of spheroids in the presence or absence of E2 was achieved by 3D cultures inside microfluidic devices as previously described^61^. Briefly, microfluidic devices were fabricated using standard soft-lithography using a mixture of 10:1 weight ratio of polydimethylsiloxane (PDMS) base to curing agent (Sylgard 184 Silicone Elastomer Kit, Dow Corning). 5 days after E2 treatment as described above, 4 × 10^5^ cells were deposited in the inlet of the device and allowed to flow through the culture chamber until cells filled the bottom of the microwells. Cells were then kept in culture in the microfluidic devices for 7 days at 37 °C in the presence of E2 or EtOH, changing media every 24 h. To track the growth of the spheroids bright-field images were acquired at days 1, 3, and 7 after seeding. Spheroid sizes were assessed using ImageJ to estimate the area at each time point, and then normalized to the area at day 1 to allow growth comparisons between wells.

### RNA-seq

RNA-seq was performed with two biological replicates (independent cultures). Approximately 500,000 DFs were used for each RNA isolation. Total RNA was extracted using RNeasy Mini Kit (Qiagen). RNA quality control was performed using Agilent RNA 6000 Nano Kit and all samples have RNA Integrity Number higher than 9.8. Total RNA (1.5 μg) was used for poly(A) mRNA selection using NEBNext Poly(A) mRNA Magnetic Isolation Module (NEB) according to the manufacturer’s protocol. Directional, strand-specific RNA libraries were prepared using NEXTflex Rapid Directional RNA-seq Kit (Bioo Scientific) according to the manufacturer’s protocol. Quality of libraries was analyzed using an Agilent bioanalyzer. Barcoded libraries were multiplexed and subjected to 80 bp single-end sequencing with an Illumina NextSeq 500 instrument.

### Native ChIP-seq (for histone variants)

Approximately 5 million cells for each preparation were used. Nuclei isolation was performed with 30,000,000 ~ 40,000,000 iDFs. Cells were resuspended with 2 ml Buffer I (0.32 M sucrose, 15 mM Tris, pH 7.5, 60 mM KCl, 15 mM NaCl, 5 mM MgCl_2_, and 0.1 mM EGTA). Then, 2 ml Buffer II (Buffer I with 0.4% NP-40) was added to the cell suspension, mixed, and incubated on ice for 10 min. The mixture was layered onto 8 ml Buffer III (1.2 M sucrose, 15 mM Tris pH 7.5, 60 mM KCl, 15 mM NaCl, 5 mM MgCl_2_ and 0.1 mM EGTA). Buffers were supplemented with 0.5 mM dithiothreitol, 0.1 mM phenylmethyl sulfonyl fluoride and 1X protease inhibitor cocktail (EDTA free). Nuclei were pelleted at 10,000 × *g* for 20 min at minimum deceleration. The supernatant was removed, and nuclei were gently resuspended with 50 μl Buffer A (0.32 M sucrose, 50 mM Tris pH 7.5, 4 mM MgCl_2_, and 1 mM CaCl_2_) per 5,000,000 cells and stored at −80 °C. For each chromatin immunoprecipitation (ChIP), an aliquot of 5,000,000 cells was thawed on ice and diluted with 350 μl Buffer A. CaCl_2_ was added to 3 mM, 8.5 units of MNase (Affymetrix) was added and the reaction was incubated at 37 °C for 10 min. The reaction was stopped by adding EGTA to 10 mM. Nuclei were spun down at 10,000 × *g* for 7 min. The supernatant was collected as S1 (mostly mononucleosomes). The pellet was gently resuspended with 400 μl Buffer B (50 mM Tris pH 7.5, 300 mM NaCl, 2 mM EDTA, and 0.1% NP-40) and extracted at 4 °C for 2 h with head-to-head rotation. Nuclei were spun down and supernatant was collected as S2 (longer chromatin fragments). S1 and S2 were pooled and further cleared at maximum speed for 5 min. Chromatin concentration was quantified spectroscopically (absorbance *A*_260_). For each immunoprecipitation, 100 μg chromatin was mixed with Buffer C (50 mM Tris pH 7.5, 150 mM NaCl, 2 mM EDTA, 0.05% NP-40) to 1 ml. Then, 50 μl was taken as input. 30 μl Magna ChIP Protein A + G magnetic beads (Millipore) were added and incubated for 2 h. After immunoprecipitation, beads were washed once with Buffer G 150 (50 mM Tris pH 7.5, 150 mM NaCl, 0.5% NP-40), twice with Buffer G 250 (50 mM Tris pH 7.5, 250 mM NaCl, 0.5% NP-40) and once with Tris-EDTA buffer (10 mM Tris pH 7.5 and 1 mM EDTA). Input and beads were incubated with 50 μg/ml RNase A for 1 h at 37 °C in 200 μl Tris-EDTA buffer. SDS was added to 0.5% and Proteinase K to 500 μg/ml. Samples were incubated overnight at 56 °C with constant mixing. Supernatant was collected from the beads. Input/ChIP DNA were purified with QIAquick PCR purification kit (Qiagen) and analyzed/quantified using Agilent 2100 Bioanalyzer High Sensitivity Kit.

### ChIP-seq

Approximately 3 million cells for histone modifications and regulators, from each sample, were used for input for native chromatin immunoprecipitation (nChIP). Cells were lysed on ice for 20 minutes in lysis buffer containing 0.1% Triton X-100, 0.1% deoxycholate, and protease inhibitor. Extracted chromatin was digested with 90 U of MNase enzyme (New England Biolabs) for 6 minutes at 25 °C. The reaction was quenched with 250 µM of EDTA post-digestion. A mix of 1% Triton X-100 and 1% deoxycholate was added to the digested samples and incubated on ice for 20 min. Digested chromatin was pooled and pre-cleared in IP buffer (20 mM Tris-HCl pH 7.5, 2 mM EDTA, 150 mM NaCl, 0.1% Triton X-100, and 0.1% deoxycholate) plus protease inhibitors with pre-washed Protein A/G Dynabeads (Thermo Fisher Scientific, Waltham, United States) at 4 °C for 1.5 h. Supernatants were removed from the beads and transferred to a 96-well plate containing the antibody-bead complex. The antibodies used are listed in Supplementary Table 2. Following an overnight 4 °C incubation, samples were washed twice with low salt buffer (20 mM Tris-HCl pH 8.0, 0.1% SDS, 1.0% Triton X-100, 2 mM EDTA, and 150 mM NaCl) and twice with high salt buffer (20 mM Tris-HCl pH 8.0, 0.1% SDS, 1% Triton X-100, 2 mM EDTA, and 500 mM NaCl). DNA-antibody complexes were eluted in elution buffer (100 mM NaHCO_3_, 1% SDS), incubated at 65 °C for 90 min. Protein digestion was performed on the eluted DNA samples at 50 °C for 30 min using protease mix (QIAGEN, Venlo, Netherlands). ChIP DNA was purified using Sera-Mag beads (Thermo Fisher Scientific) with 30% PEG before library construction. Size distribution and level of amplification were determined by analysis using Agilent bioanalyzer or Fragment Analyzer. Libraries were prepared by following a modified Illumina paired-end protocol and sequenced on an Illumina HiSeq 2500 to a median depth of ~25 million (H3K4me1 and H3K4me3) or ~50 million reads (H3K27me3 and Input) or single end protocol for histone variants to a median depth of ~80 million.

### Immunohistochemistry

Specimens were obtained from Icahn School of Medicine at Mount Sinai and considered non-human subject research. Tissue Microarray slides were provided by the NCI cancer Diagnosis program (CDP). Other investigators may have received slides from the same blocks. IHC was performed as described before^62^. In brief, 5 μm sections from formalin-fixed paraffin-embedded specimens were deparaffinized, incubated for antigen retrieval with Vector Citrate-Based Antigen Unmasking Solution (Vector Laboratories) in microwave for 10 min, and then exposed to 0.3% hydrogen peroxide to block endogenous peroxidase activity. After blocking with Vector Normal Horse Serum (2.5%) for 20 min, sections were incubated at 4 °C overnight with mH2A2 (1:350–1:500) prepared in 0.1% BSA. Slides were subsequently developed using Vector imPRESS Universal Kits anti-mouse/rabbit Ig or anti-goat Ig (Vector Laboratories), Vector DAB Peroxidase Substrate Kit as the chromagen (Vector Laboratories) and Harris Hematoxylin (Sigma) for counterstaining. Slides were then sealed and mounted with Permount (Sigma) and randomized for subsequent blinded review.

### Chromatin isolation and Western blot

Chromatin fractionation was performed as described^63^. Briefly, cells were washed in PBS and resuspended in 1 ml buffer A (10 mM HEPES pH 7.9, 10 mM KCl, 1.5 mM MgCl_2_, 0.34 M sucrose, 10% glycerol, 1 mM DTT and 1X protease inhibitor cocktail). Triton X-100 was added to 0.1% and the cells are incubated on ice for 10 min. Nuclei were collected by centrifugation at 4000 rpm at 4 °C. The supernatant was taken as the cytosolic fraction. Nuclei were washed once with buffer A and then lysed for 30 min in ‘No Salt’ buffer (3 mM EDTA, 0.2 mM EGTA, 1 mM DTT, and 1X protease inhibitor cocktail) on ice. Chromatin was pelleted by centrifugation at 4000 rpm at 4 °C and supernatant was enriched in soluble nuclear proteins. For western blotting, equal amounts of isolated chromatin, estimated by amido black (Sigma) staining, were run on an 8%, 15% or 4-15% SDS-PAGE gel, then transferred to PVDF membranes (Millipore). After blocking with Intercept® (PBS) Blocking Buffer (LI-COR) for 1 h at room temperature, the membrane was incubated with primary antibodies at 4 °C overnight. The membrane was then washed three times with PBST for 10 min and then incubated for 1 h at room temperature with appropriate secondary antibodies conjugated with Dylight (Invitrogen). After extensive washing, fluorescent detection was performed using the Odyssey® Fc imaging system (Li-Cor Biosciences). Alternatively, immunoblotting was performed as described^63^. For the quantification of the bands obtained by Western blot experiments, the relative density of the band obtained from the Odyssey® Fc imaging system after blotting with antibodies of interest was normalized to the relative density of the bands obtained by blotting with antibodies against housekeeping proteins (H3 or H4). This ratio was used to compare expressions between conditions.

### MNase immunoprecipitation

The MNase immunoprecipitation was performed by transducing ~3 × 10^6^ viable MDA-MB-231L cells with lentiviral constructs of GFP-tagged macroH2A2 or GFP-tagged H2A. After 3 days, the cells were trypsinized, washed, and counted. The total number of transfected cells was determined using a fluorescent cell counter. About 10 × 10^6^ transduced cells were used in the IP for the canonical H2A control, and for the macroH2A2 sample. 10 × 10^6^ cells were lysed in 1 ml of PBS containing 1X Complete EDTA-free protease inhibitor (Roche) and 0.2% Triton X-100 by rotation at 4 °C for 10 min. The solution was then centrifuged at 3300 × *g* for 5 min and the nuclear pellet was resuspended in 100 µl EX100 buffer (10 mM Hepes pH7.6, 100 mM NaCl, 1.5 mM MgCl_2_ 0.5 mM EGTA, 10% [v/v] glycerol, 1X EDTA-free protease inhibitor, 1 mM DTT, and 2 mM CaCl_2_ in deionized water). MNase digestion was initiated upon the addition of 0.4 µl MNase enzyme (New England Bioscience) and was carried out at 37 °C for 10 min. The reaction was then quenched with EGTA to a concentration of 10 mM. The supernatant was then collected after a 7 min spin at 10,000 × *g*. 900 µl of adjusted EX100 buffer (150 mM NaCl and 0.1% NP-40) was added to the supernatant. To immunoprecipitate the nucleosomes, 20 µl of GFP-trap magnetic beads (Chromotech) were equilibrated two times in EX100 buffer for each sample group. The supernatant was then added to the bead slurry and rotated overnight at 4 °C. The next day, the beads were washed once with cold buffer G150 (50 mM Tris-HCl pH 7.5, 150 mM NaCl, and 0.1% NP-40) and twice with cold buffer G250 (50 mM Tris-HCl pH7.5, 250 mM NaCl, and 0.5% NP-40). The immunoprecipitate was eluted by resuspending beads with 40 µl of 2X Laemmli buffer (BioRad) and boiling the samples for 5 min at 95 °C. The samples were then immediately used for Western blot, as described above. For the quantification of the immunoblot bands, the normalized values of the IP conditions were divided by the normalized values of the input conditions and this ratio was used to compare relative expression of H4K12ac between conditions.

### Mice

All mouse experiments were approved by and performed under the guidelines of the Institutional Animal Care and Use Committee (IACUC) from Icahn School of Medicine at Mount Sinai (protocol IACUC-2014-0093). MacroH2A double knockout (dKO – H2Afy;^−/−^H2AFy2^−/−^) (JAX strain 025481) were a kindly provided by Dr. John Pehrson. 129/S6 WT mouse strain was purchased from Jackson laboratory and backcrossed with the mdKO mice in order to generate a heterozygote offspring which were further inbred to generate WT and mdKO with the same background. All mice were humanely sacrificed by CO_2_ asphyxia followed by cervical dislocation as outlined by approved IACUC protocol. Mice were maintained on a 12 h day/night cycle.

### Mouse primary MEC and tumor cells isolation and FACS

Primary MECs cells were isolated from the mammary glands of 11-week-old females. Cells were initially minced and digested with 0.75 mg/ml collagenase A (Roche) in Advanced DMEM/F12 medium at 37 °C for 2 h. The tissue was further digested with 0.05% trypsin for 5 min followed by 5 mg/ml neutral dispase (Worthington) with 1000 μg/ml DNase (Roche) for 5 min. The digested cells were filtered through 40 μm cell strained to obtain single cells. Red blood cell lysis buffer and EasyStep Mouse Epithelial Cell Enrichment kit (Stem Cell) was used to negatively select blood cells. Various MEC subpopulations were FACS sorted using specific cell lineage markers (EpCAM, CD49b, CD49f, Sca1 – MECs). For antibodies list, please see Supplementary Table 2.

### Histology and whole mounting

For histological examination of mouse mammary glands, tissues were fixed in 10% Neutral Buffered Formalin overnight and embedded in paraffin. Sections (10 μm) were prepared and stained with hematoxylin and eosin (H&E). For whole-mount analysis, formalin-fixed cells were stained with carmine overnight and de-stained with acidic alcohol (70% ethanol/1% HCl) for proper staining intensity. The tissues were then dehydrated and cleared with Hemo-De clearing agent (Fisher) before imaging.

### Organoid culture

MaSC were prepared as above and cultured in Advanced DMEM/F/12 containing 3% Matrigel, 5% heat-inactivated FBS, 10 ng/ml EGF, 20 ng/ml bFGF, 4 mg/ml heparin, and 5 mM Y- 27632 in ultra-low attachment plates (Corning).

### Antibodies

The following antibodies were used in this study: H3K27me3 (Millipore 07-449), H3K27ac (Abcam ab4729, Western blot), H3K27ac (Cell signaling, 8173BC, ChIP), H3K4me1 (homemade EDL), H4K12ac (Millipore, 07-595), P300 (Clone NM11, Active Motif 61401), BRD4 (Bethyl A301-985A50, ChIP), BRD4 (Abcam ab128874, Western blot), Cas9 (Millipore MAC133), H2A.Z (Abcam ab150402), mH2A1 (Abcam ab37264, ChIP), mH2A1 (Millipore 07-219, Western blot), mH2A2 (Homemade, Bernstein Lab^23^), H3 (Abcam Ab1791), H4 (Abcam, ab177840), GFP (Roche 11814460001), Beta-Actin (Sigma, A5441), Flag (Sigma, F1804), Mouse IgG – DyLight 680 (Invitrogen SA5-10170), Rabbit IgG DyLight 800 (Invitrogen SA5-10044). The antibodies used in this study are listed in Supplementary Table 2.

### cDNA generation and RT-qPCR

Total RNA was extracted using the RNeasy Mini Kit (Qiagen) according to the manufacturer’s protocol. cDNA was generated using First-Strand cDNA Synthesis System (ORIGENE). qPCR was performed using FastStart Universal SYBR Green Master Mix (Rox) (Roche) or PowerUp SYBR Green Master Mix (Applied Biosystems). The primers used are listed in Supplementary Table 1.

### ATAC-seq

DNA for ATAC-seq was prepared from 50,000 cells following the OMNI-ATAC procedures as described by *Corces et al*^64^. with modifications using the Nextera kit (Illumina). The cells were lysed for 3 min on ice and transposed for 30 minutes at 37 °C following clean-up. The DNA libraries were prepared with 5–10 cycles of PCR amplification with the NEB High Fidelity Master Mix (New England Biolabs, Ipswich, United States). Clean-up was done using the Zymo DNA Clean and Concentrator kit (Zymo Research, Irvine, United States) and followed with AMPure XP (Beckman Coulter, Brea, United States) bead clean-up to remove primer dimers and under-digested chromatin. Sequencing was performed on an Illumina HiSeq 4000 to a depth of ~30 million reads per sample.

### Single cell ATAC-seq

Approximately 100,000 viable cells per sample were subjected to transposase assays (exposing buffered nuclei to Tn5 transposase) before proceeding to single cell partitioning into gel beads in emulsion, barcoding, library construction, and sequencing following established 10X Genomics protocols. The target cell recovery was ~8000 cells per sample. For details on the 10X Genomics Chromium platform including demonstrated protocols on sample preparation, library construction, instrument settings, and sequencing parameters please see the manufacturer’s resources (https://support.10xgenomics.com/single-cell-atac). Genomic libraries were sequenced on an HiSeq 4000 (Illumina) before demultiplexing, alignment to the reference genome, and post-alignment quality control.

### Single cell multiome-seq

Organoids from mammary stem cells were digested with 0.05% trypsin (for 8 min at 37 °C. Cells were then used for nuclei isolation procedure according to 10X genomics specification (https://support.10xgenomics.com/single-cell-multiome-atac-gex/sample-prep/doc/demonstrated-protocol-nuclei-isolation-for-single-cell-multiome-atac-gene-expression-sequencing). Approximately 5000 nuclei per sample were subjected to transposase assays (exposing buffered nuclei to Tn5 transposase) before proceeding to single cell partitioning into gel beads in emulsion, barcoding, pre-amplification, ATAC library construction, cDNA followed by GEX library construction and sequencing following established 10X Genomics protocols. The target cell recovery was ~4000 cells per sample. For details on the 10X Genomics Chromium platform including demonstrated protocols on sample preparation, libraries construction, instrument settings, and sequencing parameters please see the manufacturer’s resources (https://support.10xgenomics.com/single-cell-multiome-atac-gex). ATAC and GEX libraries were sequenced separately on an HiSeq 4000 (Illumina) before demultiplexing, alignment to the reference genome, and post-alignment quality control.

### Data processing and analysis

#### ATAC-seq

Sequenced reads from ATAC-seq experiments (HMEC and DF, one replicate each) were trimmed for adapter sequences using TrimGalore v0.4.5 with default parameters, then aligned to the corresponding reference genome (hg19 for HMEC and MDA-MB-231L; mm9 for DF) using bowtie2 v2.3.3.1^65^ with parameter (–maxins 2000). The aligned reads were filtered for alignment quality q30 and sorted using Samtools v1.9^66^ (default parameters). Duplicate read mappings were removed using Picard v2.9.0 (default parameters). Peaks were called using Macs2 v2.2.7.1^67^ with parameters -q 5e-5–nolambda–keep-dup all–slocal 10000). DeepTools v3.5.0^68^ bamCoverage with parameters (–binSize 10–scaleFactor 0.5–skipNonCoveredRegions–normalizeUsing RPKM) was used to calculate signal—number of reads per bin (bigWig) files from the alignment (BAM) files. Quality control (QC) statistics are reported in Supplementary Data 3. ataqv^69^ was used to calculate the fraction of reads in peaks (FRiP) and TSS enrichment.

#### ChIP-seq

Sequenced reads from ChIP-seq experiments (mH2A1 and mH2A2 in HMEC, NHM, MCF7 and MDA-MB-231L; H3K27me3, H3K4me1, H3K27ac, BRD4, p300 in MDA-MB-231L, one replicate each) were trimmed for adapter sequences using TrimGalore with default parameters. Reads were aligned to reference genome hg19 using bowtie with parameters (-k 1 -m 20–best -S -n 2 -l 65–chunkmbs 200) for single-end samples and using bowtie2 with parameter (–maxins 2000) for paired-end samples (Supplementary Data 3). The aligned reads were filtered for alignment quality q30 and sorted using Samtools v1.9 (default parameters) followed by duplicate removal using Picard (default parameters). Narrow peaks for H3K4me1 with corresponding input control were called using Macs2 callpeak command with parameters (–bw 150–bdg–SPMR -q 1e-2). The input-corrected signal tracks (bigwig) were obtained using Macs2 bdgcmp command with parameters (–method FE), followed by bedClip and bedGraphToBigWig commands from UCSC Genome Browser Tools^70^, both with default parameters. QC statistics are reported in Supplementary Data 3.

#### RNA-seq

Quantification of RNA-seq signal at the cell-type specific *cis*-regulatory elements (CRE) (Fig. 2a) was performed using bedtools v2.27.1^71^ multicov command (with default parameters) followed by RPKM computation using edgeR v3.36.0^72^, in cases where the alignment (BAM) files (aligned to hg19 reference genome) were readily available from ENCODE reference epigenomes (HMEC: ENCSR460EGF, MCF7: ENCSR247DVY and HepG2: ENCSR888GEN). In the case of NHM, RNA-seq reads obtained from Fontanals-Cirera et al.^73^ were aligned to hg19 reference using STAR v2.7.3a^74^ with parameters (–runMode alignReads–outFilterMultimapNmax 10–outFilterMismatchNmax 10–outFilterType BySJout–outFilterIntronMotifs RemoveNoncanonicalUnannotated–outSAMtype BAM SortedByCoordinate–quantMode GeneCounts) followed by bedtools multicov for quantification and edgeR for RPKM calculation. For enhancer expression in normal mammary tissue (Fig. 2b), RPKM quantification in 15,808 enhancers in normal breast tissue from 113 samples was obtained from Chen et al. The subset of these enhancers that overlapped with the classified CRE in HMEC were identified using bedtools intersect. The average expression across all samples in each enhancer that overlapped with CRE in the five classes are plotted as a boxplot (Fig. 2b). To analyze the expression of genes regulated by different combinations of CRE in HMEC (Fig. 2c), we obtained the RPKM quantification at protein coding regions from Roadmap Reference Epigenome E119. We obtained the association of genes with CRE by finding those CRE that overlap with known associations in GeneHancer v4.4^75^. From the resulting associations, genes were grouped into five categories—genes that associate only with inactive CRE (*Inactive only*), genes that associate only with macro bound enhancers (*mBE only*), genes that associate only with ATAC-only CRE (*ATAC only*), genes that associate with at least one one active enhancer (*Active Comb*), and genes that associate with all other combinations of CRE classes (*Comb*). Genes that were associated with super-enhancers are separately grouped. Super-enhancers were predicted using LILY as described below.

#### Super-enhancer prediction for each cell type

Super-enhancers were predicted using LILY^76^ with parameters (maxDistanceToStitch=12500, distFromTSS=2500) from H3K27ac data - narrow and broad peaks and the signal tracks. Narrow peaks, broad peaks, and bigwig signals were readily available for HMEC, NHM, and HepG2 from Roadmap Epigenomics Project (identifiers: E119, E059, and E118, respectively). For MCF7, we obtained the genomic alignment files (BAM files) for both H3K27ac target and input control from ENCODE Reference Epigenome (ENCSR247DVY) and used HMCan v1.41^77^ (with parameters: smallBinLength 50, largeBinLength 100000) as recommended by LILY documentation to produce the narrow and broad peaks and the bigwig signal track, which was then input to LILY (with parameters: maxDistanceToStitch=12500, distFromTSS=2500) for super-enhancer prediction.

#### Single cell ATAC-seq

Reads from 10x Genomics single-cell ATAC-seq experiments (MCF7 *wt* and *mH2A2KO*, two replicates each) were processed using 10x Genomics Cell Ranger ATAC v2.0.0^78^. The reads were aligned to the pre-built human reference genome GRCh38 – v2020-A-2.0.0 (May 3, 2021) provided by 10x Genomics. MCF7 specific *cis*-regulatory elements (CREs) (lifted over from hg19 to hg38 coordinates using liftOver tool) were used as the input peaks. Read trimming, alignment, duplicate marking (ATAC) and cell calling were performed by Cell Ranger. Cell Ranger ATAC aggr functionality was used to aggregate the 4 samples resulting in read-depth normalized cut-site counts. Downstream processing was done using Seurat v4.0.4^79^ and Signac v1.4.0^80^. Cells with <200 unique peaks detected (ATAC) and those with transcription start site (TSS) enrichment score (as calculated by Signac) <1, were removed for quality control resulting in 27,297 cells. QC statistics reported by Cell Ranger ATAC are listed in Supplementary Data 3. QC plots of mean TSS enrichment scores and fragment length distribution are shown in Supplementary Fig. 5a. The remaining cells after QC filtering were randomly subsampled from each sample to match the cell count of the sample with the lowest number of cells giving 19,396 cells for downstream analysis. The cut-site count matrix was normalized, dimensionality reduced and projected into UMAP space (Supplementary Fig. 5d) using Signac functions (RunTFIDF, FindTopFeatures, RunSVD and RunUMAP) with default parameters. The UMAP was calculated using the LSI components 2 to 20 (LSI1 correlates highly with read depth). Clusters were identified by computing the shared nearest neighbor graph from LSI components 2–20 using Seurat’s FindNeighbors function, followed by FindClusters function with parameters (algorithm = 3, resolution = 0.15). QC statistics per cluster, as calculated using Signac functions are shown in Supplementary Fig. 5b, statistical significance tested using Kruskal-Wallis test. Standard deviation explained by each LSI dimension is shown in Supplementary Fig. 5c. For the effect size bubble plot (Fig. 5e), we computed Cohen’s effect size comparing the means of the distributions of the number of TF binding sites per cell in the wild-type and knock-out populations. The calculation was done on TF binding sites unique to the MCF7 cells grown in estrogen, those unique to control MCF7, and the binding sites in both groups, from peaks obtained from ReMap (Biotypes: MCF-7_E2 for estrogen; MCF-7 and MCF-7_ETOH for control). *p*-values were computed using the Mann–Whitney U test. The *cis*-regulatory interactions between CRE were predicted separately for each condition (*wt* and *mH2A2KO*) using Cicero v1.3.5^81^ function run_cicero with parameters (sample_num = 100). Interactions with co-accessibility scores above 0.1 were counted as confident interactions. The distribution of interactions between CREs that belong to each pair of enhancer classes was plotted as a Circos chord diagram (Fig. 5f) using the R package Circlize v0.4.14^82^. The distribution of number of interactions per peak in *wt* and *mH2A2KO* cells are shown in Supplementary Fig. 5e. Gene set enrichment analysis (Supplementary Fig. 5g) of all the genes whose promoters gained interactions (co-accessibility score > 0.1) in MCF7 *mH2A2KO* compared to *wt* was done using gost function from gprofiler2^83^, which performs a hypergeometric test with multiple testing correction using its native g:SCS method. Promoter-enhancer interactions around *TBX2*, *SOX9,* and *HES1* genes were plotted using UCSC Genome Browser (Supplementary Fig. 5h).

#### Single cell Multiome-seq

Reads from 10x Genomics single-cell Multiome experiments (mammary stem cells in mouse - MaSC *wt* and *mH2AdKO*, two replicates each), with single cell gene expression (GEX) and single cell ATAC-seq (ATAC) assayed simultaneously for each cell, were processed using 10x Genomics Cell Ranger ARC v2.0.0. The reads were aligned to the pre-built human reference genome mm10 – v2020-A-2.0.0 (May 3, 2021). Read trimming, alignment, duplicate marking (ATAC), UMI counting (GEX), peak calling (ATAC), and joint cell calling were performed by Cell Ranger ARC. Cell Ranger ARC aggr functionality was used to aggregate the 4 samples resulting in read-depth normalized expression and cut-site counts. Downstream processing was done using Seurat and Signac. Cells with more than 35% of reads mapped to mitochondrial genes (GEX), those with <200 unique genes detected (GEX), those with <200 unique peaks detected (ATAC), and those with transcription start site (TSS) enrichment score (as calculated by Signac) (ATAC) <1, were removed for QC. QC statistics reported by Cell Ranger ARC are listed in Supplementary Data 3. The resulting 4242 cells were randomly subsampled from each sample to match the cell count of the sample with the lowest number of cells giving 3116 for downstream analysis. The RNA count matrix was normalized, dimensionality reduced, and projected into UMAP space (Fig. 7b, c) using Seurat functions (NormalizeData, FindVariableFeatures, ScaleData, RunPCA and RunUMAP) with default parameters. PCA was calculated on the top 3000 most variable features and the UMAP was calculated using PCs 1–50. The cut-site count matrix was normalized, dimensionality reduced, and projected into UMAP space using Signac functions (RunTFIDF, FindTopFeatures, RunSVD, and RunUMAP) with default parameters. The UMAP was calculated using the LSI components 2–50 (LSI component 1 correlates highly with read depth). Clusters were identified by computing the weighted nearest neighbor graph using PCs 1–50 and LSI components 2–50 together using Seurat’s FindMultiModalNeighbors function, followed by FindClusters function with parameters (algorithm = 3, resolution = 0.12). The clusters were identified as cell-types - Basal, Luminal progenitors and Luminal Mature based on their gene markers (Supplementary Fig. 8c, d). Differential gene expression testing (Fig. 7d) was done on the log-normalized counts using Seurat’s FindMarkers function with parameters (min.pct = 0). The statistical test applied was the Mann–Whitney *U* test with *p*-values adjusted using Bonferroni correction based on the total number of genes in the dataset. Differential gene expression testing comparing *wt* and *mH2AdKO* was done for each cell type independently (although shown together in the volcano plots for efficient visualization). The *cis*-regulatory interactions and co-accessibility scores were predicted separately for each condition (*wt* and *mH2AdKO*) using Cicero function run_cicero with parameters (sample_num = 100). Interactions with a co-accessibility score above 0.1 were counted as confident interactions to calculate the distribution of interactions per peak (Fig. 7e). Differential enrichment analysis of binding sites of DNA binding proteins in the open chromatin regions (Fig. 7f) was done using Fisher’s exact test on the number of overlaps between binding sites of each protein in ReMap 2022 (mm10 reference genome), with the open chromatin regions identified for each genotype (*wt* and *mH2AdKO*). The testing was done separately for each cell type. The overlaps were identified using bedtools intersect. The set of open chromatin regions per cell type per genotype was called by filtering only those peaks that had a non-zero cut site count in at least 10% of cells in the group. The same analysis was done after removal of ReMap binding sites that overlap transcription start sites to obtain Supplementary Fig. 8e.

#### Comparison of ChIP-seq signals against chromatin state model (Fig. 1a)

Imputed signal tracks (bigwig) for 14 histone marks - H3K4me3, H3K9ac, H3K27ac, H3K79me2, H3K4me1, H2A.Z, H3K9me3, H3K27me3, H4K20me3, H3K36me3, H2BK12ac, H2BK120ac, H2BK5ac and H4K8ac, and the 25-state chromatin state model based on imputed data (BED) were downloaded from the Roadmap Epigenomics Project^31^, for reference epigenomes of HMEC, NHM, and HepG2 (EIDs: E119, E059, and E118 respectively). The average signal scores per genomic region in the chromatin state model were then calculated using computeMatrix program from deepTools with parameters (scale-regions–binSize 50–regionBodyLength 50) for all 14 histone marks and for mH2A1 and mH2A2 (from this study). Median signal scores for each chromatin state across all genomic regions in the state, were plotted as heatmap using the R program heatmap.2 with parameters (scale = ‘column’). Mann–Whitney *U* test with Bonferroni correction was performed to calculate the statistical significance of the difference in scores in each state compared to those in all other states, for each histone mark or variant.

#### Cell-type specific CRE classification

The following procedure as shown in Fig. 1b was adopted for each cell-type to subset and classify cell-type specific *cis*-regulatory elements (CRE) from ENCODE candidate *cis*-regulatory elements (cCRE). First, peaks that were common in both ATAC-seq and H3K4me1 were obtained using bedtools intersect. Then peaks that overlap ENCODE blacklist v2^84^ regions—regions that are known to have anomalous, unstructured, or high signals were removed. From this list, only peaks whose center overlapped with at least one ENCODE cCRE Registry V3^32^ (downloaded using the UCSC Table Browser^85^) were chosen for downstream analysis. The signal intensity for each peak was calculated as the summation of the input-corrected signals (raw signals for ATAC-seq) over a window of 2000 base pairs around the center of each peak using computeMatrix program from deepTools with parameters (reference-point–referencePoint center–upstream 1000–downstream 1000). The signal intensities for each peak were then normalized by the total intensity, then multiplied by a scale factor of 10,000 and then log-transformed. The z-scores of these log-normalized intensities of the six signals – H3K4me1, H3K4me3, H3K27ac, H3K27me3, macroH2A1 and macroH2A2 (with H2A.Z and CTCF, for those cell-types where data for these signals was available), were fed as input to *k*-means clustering algorithm with *k* = 5 to classify the peaks set into 5 clusters. The choice of *k* = 5 was made by analyzing the average silhouette scores (Supplementary Fig. 1a) and the number of biologically meaningful classes of open chromatin regions that can be assigned based on the enrichment of the six signals used for classification. The clusters were then named as Active, APL, ATAC-only, Inactive and mBE, based on the signal(s) that identify each cluster (Fig. 1c and Fig. 3a). Mann–Whitney *U* test with Bonferroni correction was used to test for statistical significance of the difference in signal in each cluster compared to all other clusters, for each histone mark (or variant). To further validate this classification, we compared the overlap enrichment of the classified CRE peaks against a chromatin state model built using chromHMM v1.23^34^ with 11 histone marks - H3K4me1, H3K27ac, H3K9ac, H3K4me3, H2A.Z, CTCF, H3K9me3, H4K20me1, H3K79me2, H3K36me3, H3K27me3 from the reference epigenome E119 (for HMEC), and the histone variants mH2A1 and mH2A2 (Supplementary Fig. 2a, b). The chromHMM commands BinarizeBam, LearnModel, and OverlapEnrichment were all run using default parameters. We picked the 13-state chromatin model since it was the most biologically interpretable model.

#### Analysis of cell-type specific CRE classes

Genomic annotation enrichment analysis of the CRE peaks in each class (Supplementary Fig. 1b and Fig. 3b) was done using Genome Association Tester (GAT)^86^ v1.3.4 with promoter, 5’ UTR, exon, intron, 3’ UTR, transcription termination site (TTS) and intergenic region annotations for hg19 and mm9 genomes obtained from HOMER v4.11^87^. Contig positions obtained from UCSC Genome Browser (Map Contigs track) for each genome were used as corresponding workspaces. CpG Island annotations obtained from HOMER for each genome were used as isochore in the GAT enrichment test, since CpG islands correlate with peaks in genic regions and this known effect is not of interest here. ChIP-seq signal heatmaps (Fig. 1e) were produced using the plotHeatmap program from deepTools with default parameters. To classify the HMEC CRE as super-enhancers, we predicted super-enhancer peaks from H3K27ac data using LILY, then called any CRE that overlapped at least one predicted super-enhancer peak, as a super-enhancer CRE. Intervene v0.6.5^88^ was used to create the upset plots of intersection of CRE peaks in each class between the four cell lines, the fraction of overlap of cell-specific CRE with cell-specific super-enhancers, and the pairwise fraction of overlap between all sets of peaks (Fig. 2d, Supplementary Figs. 1e and 2d, e). Cistrome-GO^89^ was used to perform ontology analyses of gene regulation by macro bound enhancers (mBE) and active promoter-like CRE (APL) in both mammary epithelial cells (HMEC) and MCF7 cells. Given a set of peaks, Cistrome-GO ranks genes by their likelihood of being regulated by TFs binding at those peaks by calculating a regulatory potential score defined as the weighted sum of peak contributions, then performs pathway enrichment analysis based on gene ranks using the minimum hypergeometric (mHG) test^90^. We use this method to assess the pathway enrichment of genes that are likely targets of mBE peaks that are common to both breast cell lines (HMEC and MCF7). We report the top 5 most significant KEGG pathways (Fig. 2e) sorted by Benjamini-Hochberg adjusted *p*-value of the mHG test performed by Cistrome-GO. Enrichment of TF and chromatin binding factors after 48 h of OSKM expression, at CRE sites of each CRE class of dermal fibroblasts (Fig. 3d) was calculated using GAT with the CRE classes as annotations, and TF and chromatin binding factors ChIP-seq peaks as segments of interest. The combined peaks of all TF and chromatin binding factors used in this analysis merged using bedtools merge was used as the workspace. Log (base 2) of fold change from this analysis is shown as a heatmap (Fig. 3d) with enrichments that were not statistically significant (Benjamini-Hochberg corrected *p* > 0.05) shown in gray. Enrichment of binding sites of TF and DNA-binding molecules at CRE sites of each CRE class of MCF7 cells was calculated using the R package ReMapEnrich v0.99.0. CRE sites from each CRE class of MCF7 cells were tested against a catalog of ChIP-seq peaks downloaded from ReMap 2022 (biotypes: MCF-7, MCF-7_E2, and MCF-7_ETOH) with parameter byChrom = TRUE. The effect size, defined as the log (base 10) ratio between the observed and expected number of overlaps, is shown as a heatmap (Fig. 5a) for those molecules whose binding sites are significantly enriched (Benjamini-Hochberg corrected *p* < 0.05) in all CRE classes. To calculate enrichment of breast cancer risk variants in each CRE class of HMEC, MCF7 and 231 L cells, GARFIELD v2^91^ was used with default parameters. GWAS summary association statistics for breast cancer risk variants were obtained from Michailidou et al.^42^. Statistical significance of enrichments using variants below GWAS *p*-value threshold < 5 × 10^−8^, as calculated by GARFIELD are reported as volcano plot (Fig. 4d). The principal component analysis of the H3K27ac signals from different breast cancer subtypes in the mBE CRE sites compared to that in all CRE sites (Fig. 4c) was done using deepTools programs multiBigwigSummary and plotPCA with default parameters with the corresponding signal track (bigwig) and peaks (BED) files. For MDA-MB-231L cell-line, *k*-means was performed with *k* = 4 instead of 5 since we do not expect to see the mBE cluster as this cell-line is devoid of macroH2A. Based on the fold change (FC) of BRD4 signals between mH2A2 over-expression and control GFP over-expression, the CRE peaks were classified into 3 groups – BRD4 Loss (FC < 0.5), BRD4 Neutral (0.5 < FC < 1.5) and BRD4 Gain (FC > 1.5) (Supplementary Fig. 6b). ChIP-seq peaks from ReMap 2022 that are significantly enriched in peaks that lost BRD4 (Fig. 6d) were calculated using ReMapEnrich. CUT&RUN peaks for BRD4 short and long isoforms (Fig. 6c) were obtained from Chen et al. Peaks that were exclusive to the short and long isoforms and those that were common in both, were identified using bedtools intersect. CrossMap v0.5.2^92^ with the appropriate UCSC chain files was used to liftOver bigwig or bed files between hg19 and hg38 wherever necessary. ChIP-Seq signal scores per peak were calculated as described in the CRE classification procedure above. UCSC Genome Browser^93^ was used for genomic visualization of ATAC-seq and ChIP-seq signal tracks.

#### Analysis of macroH2A variants at the binding sites of reprogramming factors (Fig. 3c)

To quantify the enrichment of signals of macroH2A variants and H3K27me3 at the binding sites of Oct4, Sox2, Klf4 and cMyc, relative to the signals at active TSS regions in dermal fibroblasts, we define the dermal fibroblasts specific active TSS peaks as follows. First, we define a set of peaks that include 500 bp upstream and 500 bp downstream of all transcription start sites in mm9 genome as TSS peaks. Sequencing reads from RNA-seq experiments on dermal fibroblasts (2 replicates) were then aligned to mm9 reference genome using STAR followed by quantification of expression at the TSS peaks using bedtools multicov and RPKM computation using edgeR. TSS peaks that had an average RPKM > 1 were defined as active TSS peaks. We calculated the average signal scores of H3K27me3, mH2A1, and mH2A2 at the ChIP-seq peaks of Oct4, Sox2, Klf4, and cMyc (peaks obtained from Chronis et al.), and at active TSS peaks, using computeMatrix with parameters (scale-regions–binSize 50–regionBodyLength 50). Enrichments of H3K27me3, mH2A1, and mH2A2 signals at the reprogramming factor binding sites relative to the signals at active TSS peaks are represented as the fold change of median signal at binding sites of each factor over the median signal at active TSS peaks, statistical significance of enrichment tested using Mann–Whitney *U* test.

### Statistics and reproducibility

Data are presented as median (and standard error) unless denoted otherwise. Medians were the preferred measure of central tendency and non-parametric hypothesis tests were used for comparisons unless stated otherwise. Continuous variables were compared using the Mann–Whitney *U* test, categorical variables using Fischer’s exact test. Statistical tests resulting in *p* < 0.05 are considered statistically significant. Multiple testing correction is performed using Bonferroni correction unless specified otherwise. *p* values, number of samples, and the statistical test used, are reported in the respective figure captions. All computer programs used for analyzing the data, computing statistics, and generating plots are made publicly available as a GitHub repository (https://github.com/LabFunEpi/mBE) for reproducibility.

### Reporting summary

Further information on research design is available in the Nature Portfolio Reporting Summary linked to this article.

## Supplementary information

Supplementary Information

Description of Additional Supplementary Files

Supplementary_Data_1

Supplementary_Data_2

Supplementary_Data_3

Supplementary_Data_4

Supplementary_Data_5

Supplementary_Data_6

Supplementary_Data_7

Supplementary_Data_8

Supplementary_Data_9

Supplementary_Data_10

Supplementary_Data_11

Reporting Summary

## Data Availability

The ChIP-seq and ATAC-seq datasets generated and analyzed in this study have been deposited into the NCBI Gene Expression Omnibus (GEO) data base (https://www.ncbi.nlm.nih.gov/geo/) with accession number GSE171599. The reference to the source of all data used in this study along with accession numbers and object identifiers are listed in Supplementary Table 3. The versions and references/links of all software tools and public databases used in this study are listed in Supplementary Table 4. The source data underlying all graphs and charts are provided as Supplementary Data 4–10. Uncropped western blots are available in Supplementary Data 11.
