# Supplementary material for: MacroH2A histone variants modulate enhancer activity to repress oncogenic programs and cellular reprogramming
Source: Commun Biol. 2023 Feb 23;6:215. doi: 10.1038/s42003-023-04571-1 (PMC9950461; doi:10.1038/s42003-023-04571-1)
Supplement: Supplementary file 1 — Supplementary Information [file 42003_2023_4571_MOESM1_ESM.pdf]

MacroH2A histone variants modulate enhancer activity to repress oncogenic programs and cellular reprogramming

Wazim Mohammed Ismail[+], Amelia Mazzone[+], Flavia G. Ghiraldini, Jagneet Kaur, Manvir Bains, Amik Munankarmy, Monique S. Bagwell, Stephanie L. Safgren, John Moore-Weiss, Marina Buciuc, Lynzie Shimp, Kelsey Leach, Luis F. Duarte, Chandandeep S. Nagi, Saul Carcamo, Chi-Yeh Chung, Dan Hasson, Neda Dadgar, Jian Zhong, Jeong-Heon Lee, Fergus J. Couch, Alexander Revzin, Tamas Ordog, Emily Bernstein, Alexandre Gaspar-Maia

[+] These authors contributed equally to this work

Supplementary Figure 1. Classification of cell-specific *cis*-regulatory elements

Supplementary Figure 2. Validation and characterization of classified cell-specific *cis*-regulatory elements

Supplementary Figure 3. iPS reprogramming and dCas9-mediated enhancer targeting strategies

Supplementary Figure 4. mBE characterization of MCF7 clones with mH2A2 depletion via CRISPR-Cas9 and over-expression of mH2A2 in MDA-MB-231L cells

Supplementary Figure 5. Single-cell ATAC analysis of MCF7 CRISPR/Cas9 clones

Supplementary Figure 6. Characterization of BRD4 and ZMYND8 in MDA-MB-213L cells with mH2A2 over-expression

Supplementary Figure 7. Phenotypic analysis of mammary glands from mH2A double knockout mice

Supplementary Figure 8. Multiome analysis of mammary stem cells derived from mH2A double knockout mice

---

Supplementary Table 1. Primers used in this study

Supplementary Table 2. Antibodies used in this study

Supplementary Table 3. Sequencing data sources

Supplementary Table 4. Software and databases referenced used in this study

Supplementary Figure 1

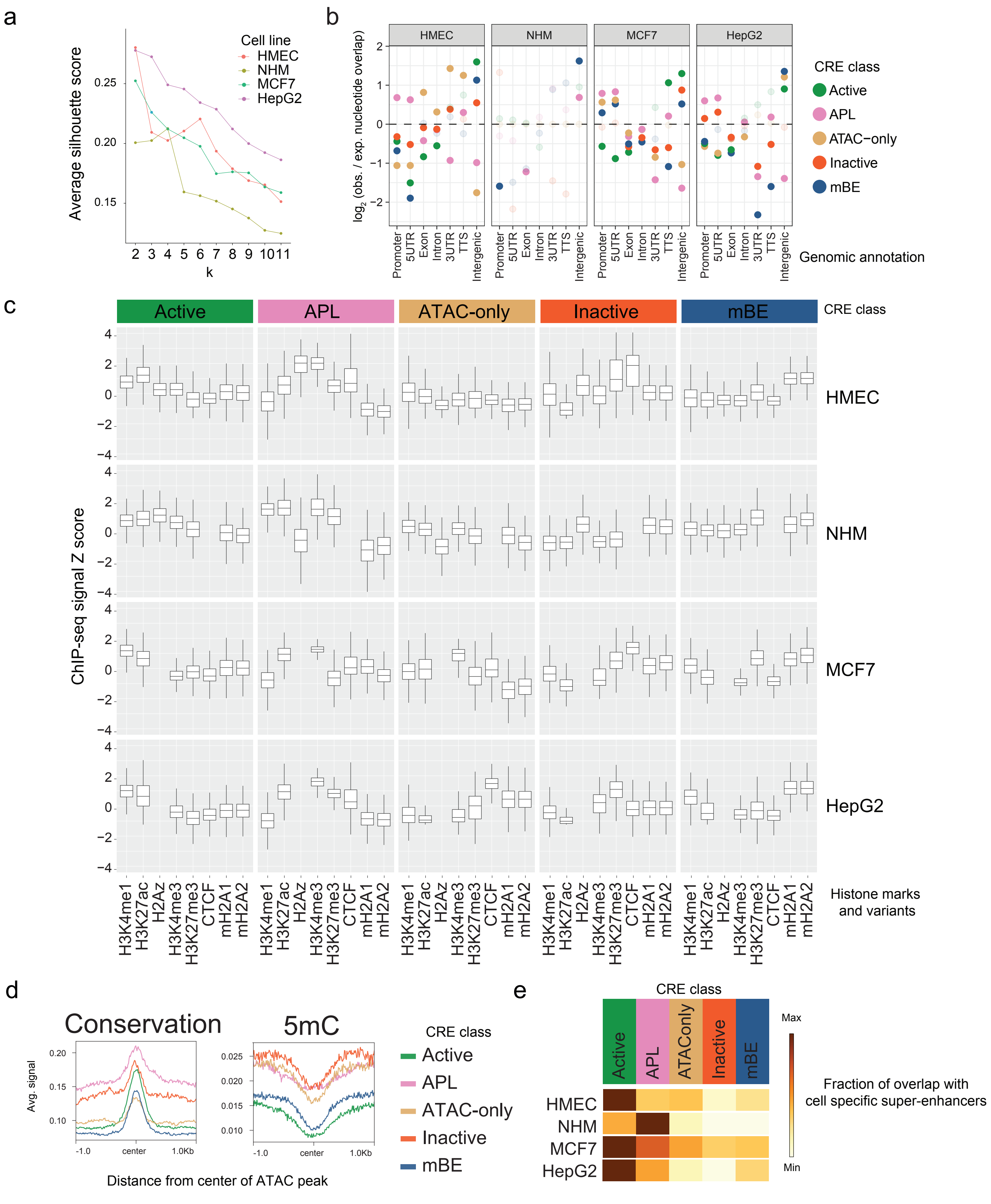

Supplemental Figure 1. Classification of cell-specific *cis*-regulatory elements. a) Average silhouette coefficient for k-means clustering using different numbers of centers. b) Genomic region enrichment of the CRE peaks in each class as calculated by GAT for each cell-type, respectively (enrichments that are statistically significant, Benjamini-Hochberg corrected p-value < 0.05, are shown in darker colors, and the rest in lighter colors). c) Boxplots showing the distributions of the Z scores of the log-normalized input-corrected ChIP-seq signals of the 8 histone marks/variants used to classify cell-specific *cis*-regulatory elements in 4 cell types – human mammary epithelial cells (HMEC), normal human melanocytes (NHM), the breast cancer cell line (MCF7) and the human hepatocellular carcinoma cell line (HepG2). The middle line represents the median, the lower and upper edges of the rectangle represent the first and third quartiles and the lower and upper whiskers represent the interquartile range (IQR) x 1.5. The number of datapoints, n, equals the number of CRE per class shown in Fig.1c. d) Signal profile of Conservation (phastCons scores for Human, hg19, from UCSC) and Methylation signal (from RoadMap reference epigenome E119) around open chromatin regions grouped by the five CRE classes. e) Heatmap showing the fraction of overlap of CRE in each class with super-enhancers predicted individually for each cell-type using LILY with H3K27ac data from Roadmap Epigenomics Project for human mammary epithelial cells (HMEC), normal human melanocytes (NHM) and the hepatocellular carcinoma cell line (HepG2), and from ENCODE Reference Epigenome for the breast cancer cell line (MCF7). The color scale is min-max scaled to compare fractions across all cell types. The maximum overlap ranged from 0.22 in NHM to 0.93 in HMEC.

Supplementary Figure 2

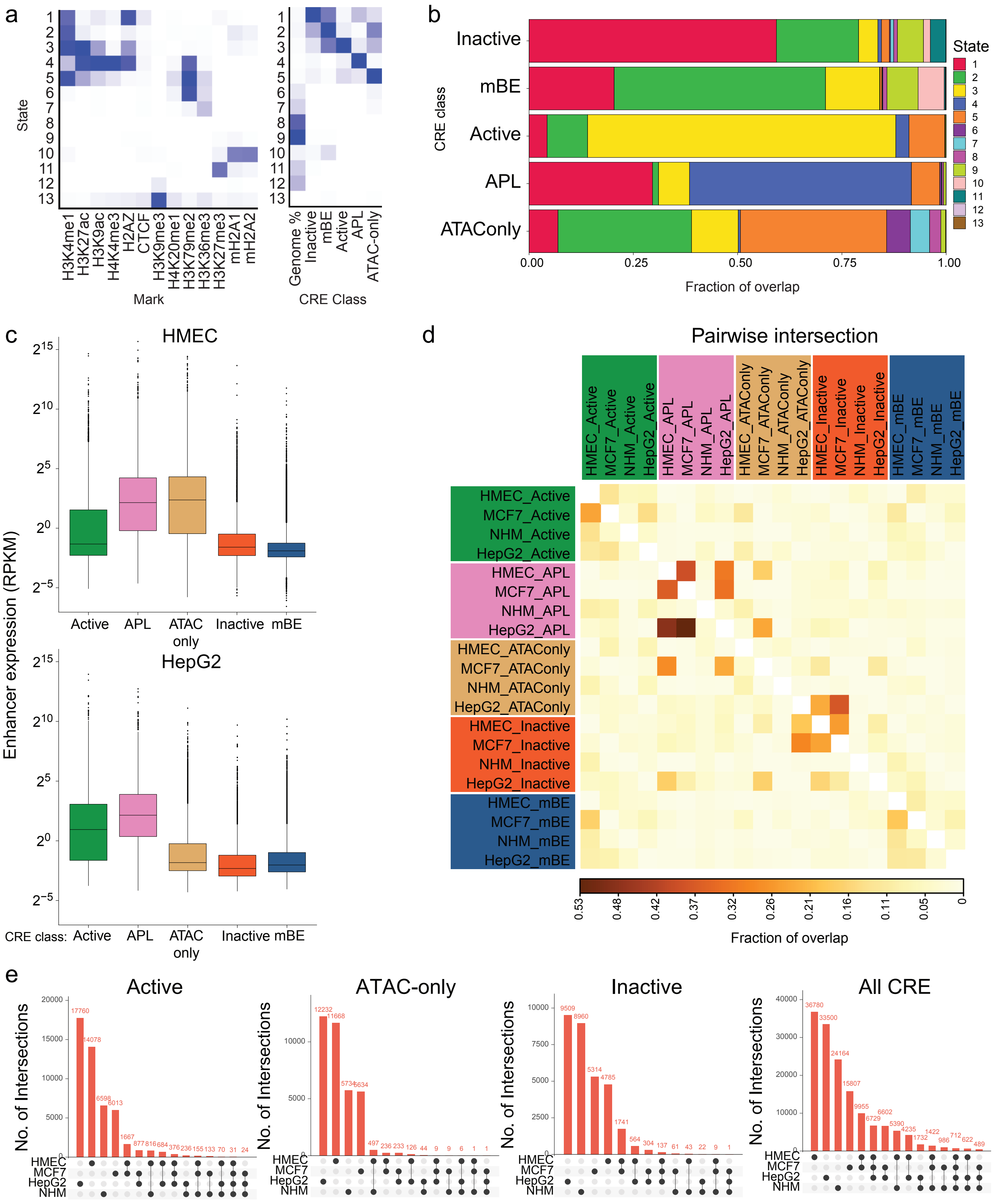

Supplemental Figure 2. Validation and characterization of classified cell-specific *cis*-regulatory elements. a) Heatmaps showing the emission parameters (left) of the 13-state chromatin model built using chromHMM with 13 signals - 11 histone marks from the Roadmap reference epigenome E119 and the histone variants mH2A1 and mH2A2, and the overlap enrichment (right) of states with the five CRE classes – Active, APL (Active Promoter-Like), ATAC-only, Inactive and mBE (macro-Bound Enhancers), from human mammary epithelial cells (HMEC), as calculated by chromHMM. b) Fraction of overlap in base pairs of each CRE class with each chromatin state predicted by chromHMM shown as a stacked bar plot. c) Expression levels at the CRE grouped by the five classes in human mammary epithelial cells (HMEC) and the hepatocellular carcinoma cell line (HepG2) quantified by RNA-seq from ENCODE's polyA-RNA datasets. The expression data is represented as boxplots where the middle line represents the median, the lower and upper edges of the rectangle represent the first and third quartiles and the lower and upper whiskers represent the interquartile range (IQR) x 1.5. Outliers beyond the end of the whiskers are plotted individually. The number of datapoints, *n*, equals the number of CRE per class shown in Fig. 1c. d) Pairwise intersection heatmap representing fraction of overlap between CREs in all four cell lines. e) Upset plots showing the intersection of Active, ATAC-only, Inactive and all CREs between the four cell lines.

Supplementary Figure 3

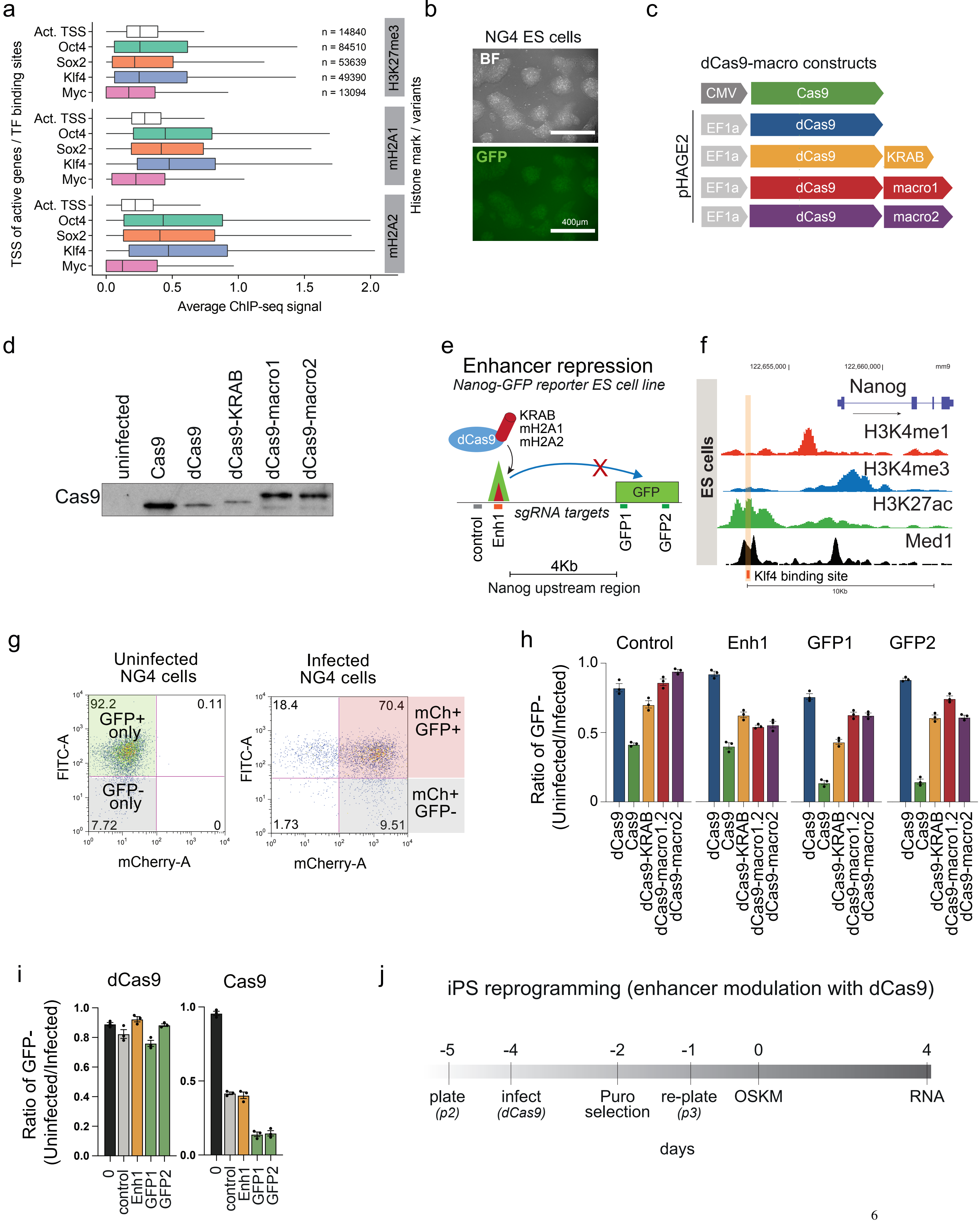

Supplemental Figure 3. iPS reprogramming and dCas9-mediated enhancer targeting strategies. a) Boxplots showing the distribution of the average input-corrected ChIP-Seq signals of macroH2A variants and H3K27me3 at the binding sites (BS) of Oct4, Sox2, Klf4 and cMyc (48hrs after OSKM induction, binding sites data collected from Chronis et al.[37]) and at active TSS (AT) regions in dermal fibroblasts. Active TSS (AT) regions are defined as 500 bp up- and downstream of the transcription start sites that have RPKM > 1 measured by RNA-seq experiments on dermal fibroblasts. The middle line represents the median, the lower and upper edges of the rectangle represent the first and third quartiles and the lower and upper whiskers represent the interquartile range (IQR) x 1.5. The number of datapoints, *n*, in each distribution is as shown in the plot. b) NG4 embryonic stem cell line engineered to contain a Nanog::GFP transgene[38]. Scale bar, 400 μm. c) dCas9 constructs with a pHAGE2 lentiviral backbone. d) Immunoblot of Cas9 for all constructs in NG4 whole cell extracts. e) Schematic of enhancer targeting using dCas9 chimaeras and sgRNAs complementary to regions around the Nanog regulatory upstream region and the transgenic GFP gene in the Nanog-GFP ES cell line NG4. f) UCSC genome browser snapshot of the Klf4 binding site upstream of the Nanog TSS in an ES cell line with H3K4me1, H3K4me3, H3K27ac and Med1 (modENCODE). g) GFP/mCherry flow cytometry plots of uninfected NG4 stem cells and infected with the sgRNA vector with mCherry reporter. h) Ratio of differentiated cells (GFP negative) between experimental and uninfected NG4 cells. Experimental conditions are cells expressing Cas9 or dCas9 constructs with sgRNAs targeting regions around the Nanog regulatory region. Data represented as mean with SE (n=3). i) Data represented are ratio of GFP cells over control infection from FACS with dCas9 alone and Cas9 after sgRNA targeting. Data represented as mean with SE (n=3). j) Schematic of iPS reprogramming studies of DFs with dCas9 infections of series shown in c and sgRNA targeting shown in e.

Supplementary Figure 4

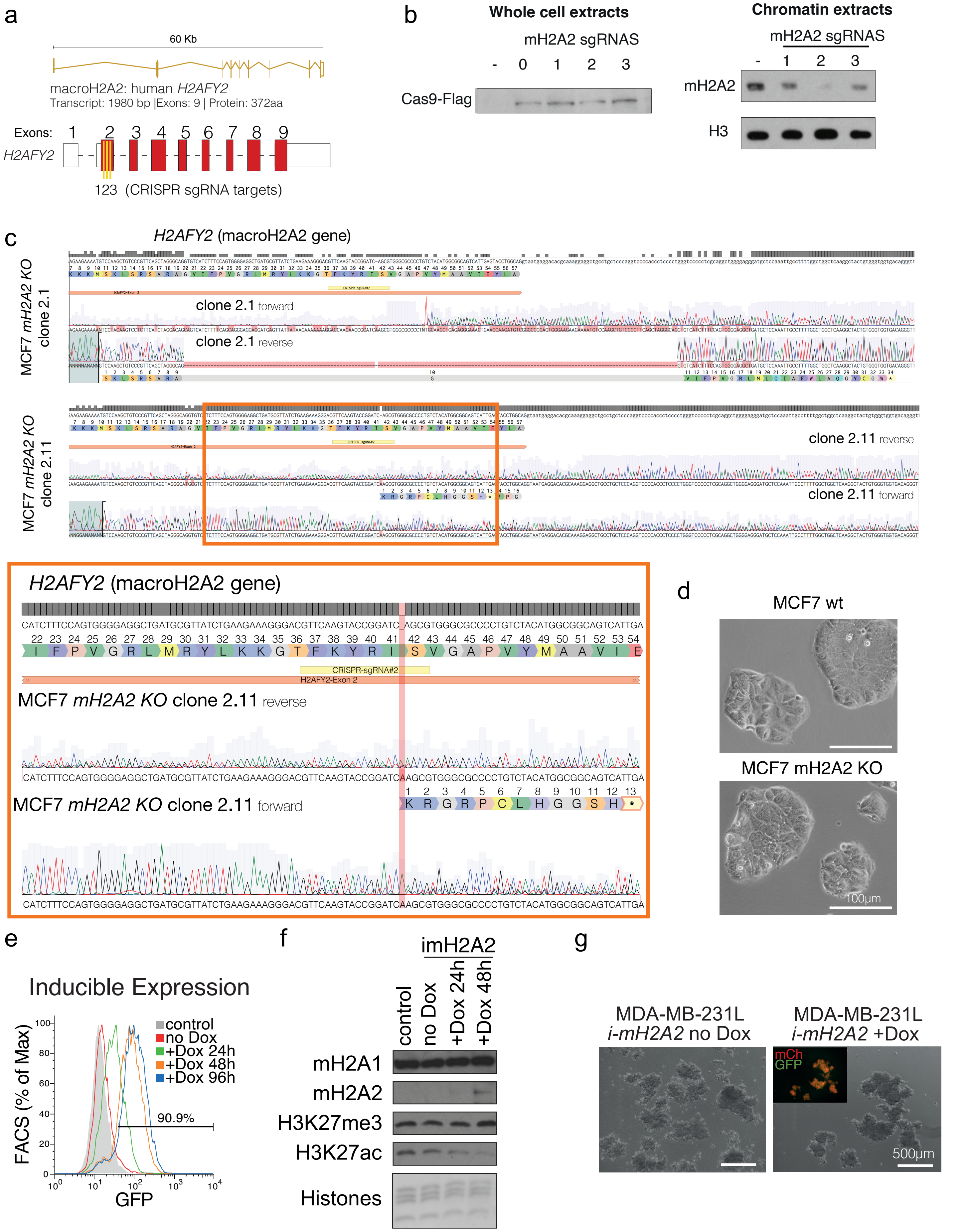

Supplemental Figure 4. mBE characterization in MCF7 clones with mH2A2 depletion via CRISPR-Cas9 and over-expression of mH2A2 in MDA-MB-231L cells. a) Targeting of the *H2AFY2* gene encoding mH2A2 with CRISPR/Cas9. b) Immunoblots of whole cell extracts for Cas9 (using a Flag antibody), and chromatin extracts for mH2A2 and H3 as a loading control in MCF7 cells transduced with Lenti-Cas9 and sgRNA targets 1-3. c) Sanger sequencing of two independent MCF7 clones showing genome editing of the sgRNA#2 locus. d) Brightfield image of MCF7 clones. e) Induction of mH2A2-GFP expression analyzed by FACS upon addition of doxycycline. f) Induction of mH2A2-GFP expression analyzed by western blot upon addition of doxycycline. g) Tumorsphere formation after induction of mH2A2-GFP expression (brightfield and fluorescence: mCherry and GFP).

Supplementary Figure 5

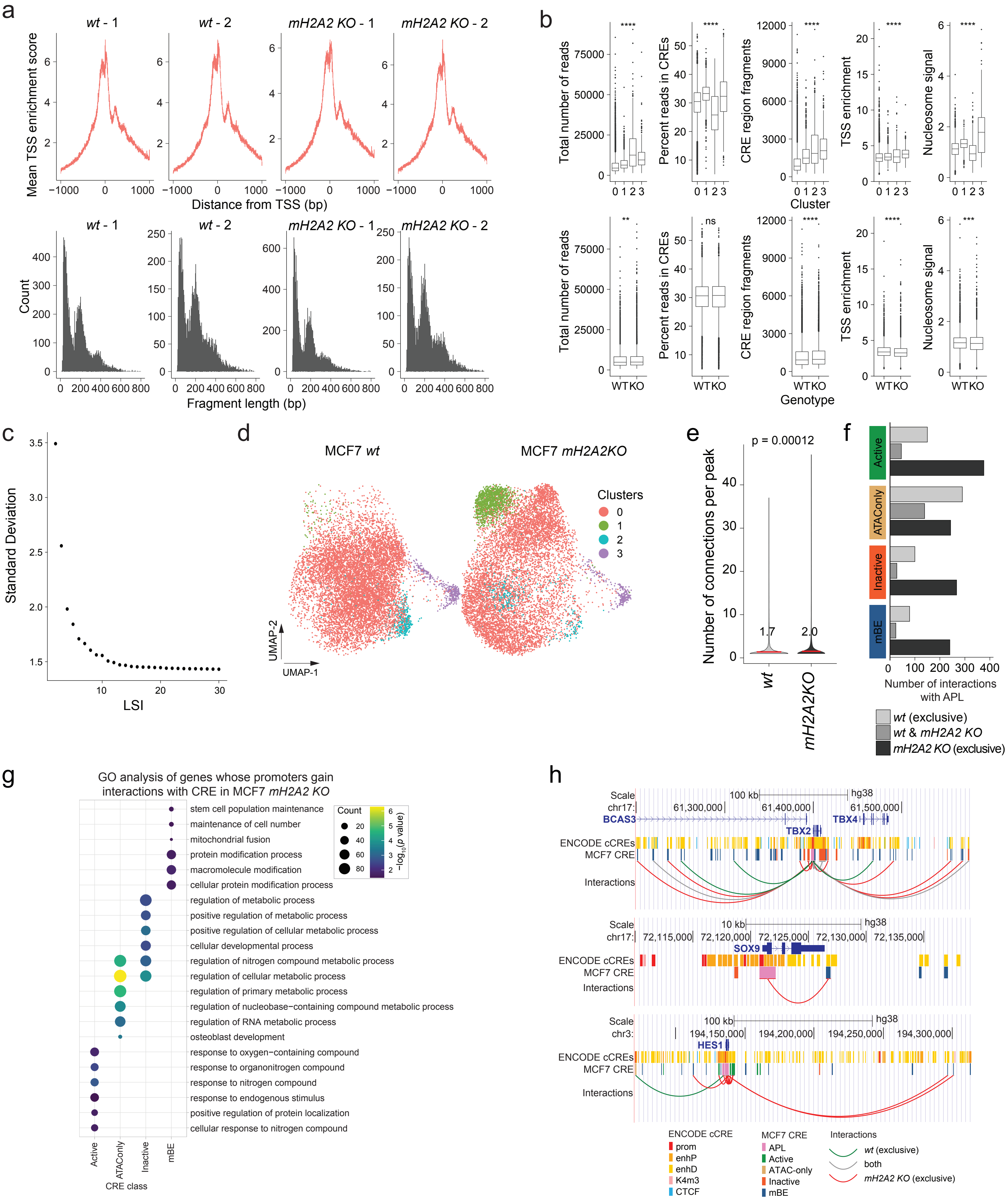

Supplemental Figure 5. Single-cell ATAC analysis of MCF7 CRISPR/Cas9 clones. a) (Top) Mean transcriptional start site (TSS) enrichment score - the ratio of fragments centered at the TSS to fragments in TSS-flanking regions, for each sample. (Bottom) Histograms of fragment length for all the cells per sample showing the periodicity of nucleosomes in the scATAC-seq data. b) Boxplots showing QC statistics per cluster and per genotype. The middle line represents the median, the lower and upper edges of the rectangle represent the first and third quartiles and the lower and upper whiskers represent the interquartile range (IQR) x 1.5. c) Standard deviation along each Latent Semantic Indexing (LSI) dimension used for choosing the number of dimensions used in the graph-based clustering. d) UMAP plot of cells showing clusters identified by graph-based clustering of scATAC-seq signals from MCF7 wild type (*n* = 9698) and MCF7 mH2A2 KO (*n* = 9698) (two replicates each) after 5 days of treatment with E2. e) Violin plot showing the number of interactions per open chromatin region with interactions (co-accessibility score > 0) predicted from the scATAC-seq data using Cicero. In each violin plot the comparison of means was done using the Mann-Whitney U test. Mean values are shown for each violin. The middle line represents the median. The number of datapoints, *n*, equals the number of CRE per class in MCF7 shown in Fig.1c. f) Number of interactions between active promoter-like (APL) elements and each of the four other classes, predicted by Cicero with co-accessibility score greater than 0.1, grouped by interactions exclusively in MCF7 *wt*, those exclusively in MCF7 *mH2A2KO*, and those in both genotypes. g) Gene set enrichment analysis of genes whose promoters gained interactions with each of the four other classes, in MCF7 *mH2A2KO* compared to *wt*. Only the top 6 most significant Gene Ontology (Biological Process) terms by p-value are shown. p-values are from hypergeometric test with multiple testing correction done by g:Profiler's native g:SCS method. The number of gene hits with the term are shown by the size of the circle. h) UCSC Genome Browser view of ENCODE candidate *cis*-regulatory elements, MCF7 specific *cis*-regulatory elements and promoter-enhancer interactions around *TBX2*, *SOX9* and *HES1* genes.

Supplementary Figure 6

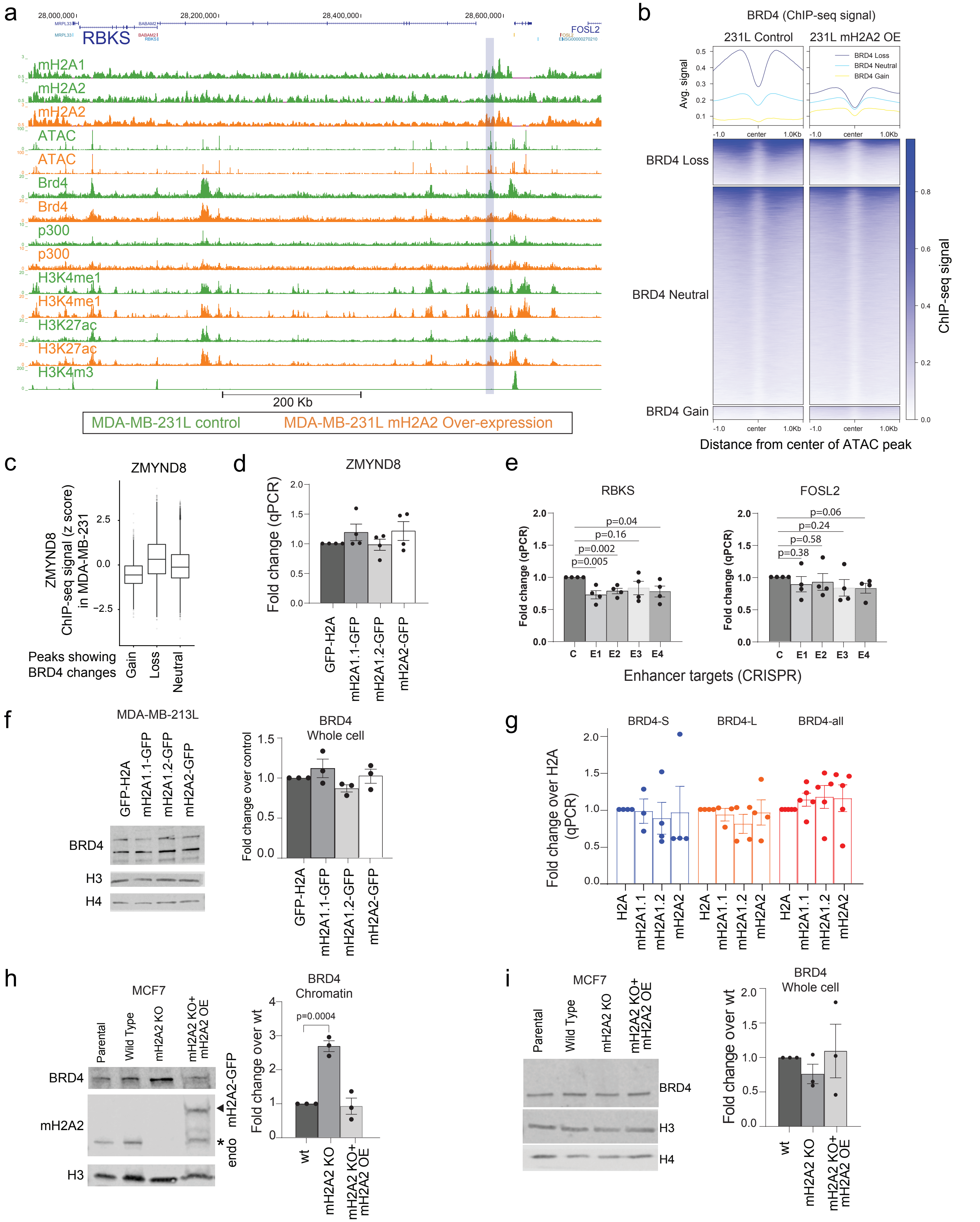

Supplemental Figure 6. Characterization of BRD4 and ZMYND8 in MDA-MB-213L cells with mH2A2 over-expression. a) USCS genome browser snapshot of a representative BRD4 peak lost with mH2A2-GFP overexpression. Green tracks represent GFP control sample; Orange tracks represent mH2A2-GFP sample. b) Average signal profile (top) and heatmaps (bottom) of ChIP-seq signal scores of BRD4 around open chromatin regions (defined by ATAC-seq) grouped by BRD4 Loss (FC < 0.5, *n* = 8362), BRD4 Neutral (0.5 < FC < 1.5, *n* = 39575) and BRD4 Gain (FC > 1.5, *n* = 2391) regions in MDA-MB-213L control vs mH2A2 over-expression cells. c) Boxplots showing distributions of the Z scores of the log-normalized input-corrected ChIP-seq signal of ZMYND8 (data from Chen et al[47].) around open chromatin regions (defined by ATAC-seq) grouped by BRD4 Loss (*n* = 8362), BRD4 Neutral (*n* = 39575) and BRD4 Gain (*n* = 2391) regions in MDA-MB-213L control vs mH2A2 overexpression cells. d) qPCR using RNA extracted from MDA-MB-231L cells with over-expression of mH2A-GFP constructs (and H2A-GFP as control) using primers against ZMYND8. Data are mean with SE, n=4 (p>0.05, t-test). e) qPCR using RNA extracted from MDA-MB-231L+Lenti-Cas9 targeting sgRNAs against mBE enhancers (E1-4) that have lost BRD4 enrichment upon mH2A2 over-expression. Control (C) sgRNA is a non-targeting vector. P values are indicated in the figure, n=3-4 (t-test). f) Immunoblots from whole cell extracts in MDA-MB-231L cells with over-expression of mH2A-GFP constructs (and H2A-GFP as control) probed for BRD4 and histones H3 and H4. Fold change quantification over control (H2A-GFP) after H3 normalization (right). Data are mean with SE (n=3, p>0.05, t-test). g) qPCR using RNA extracted from MDA-MB-231L cells with over-expression of mH2A-GFP constructs (and H2A-GFP as control). Primers (Supplementary Table 1) are designed to detect the either the short (blue) or long (orange) variants of BRD4 only, or total BRD4 (red). Data are mean with SE n=3-5 (p>0.05, t-test). h) Immunoblots from chromatin extracts in MCF7 clones with knockout or over-expression of mH2A-GFP constructs (and H2A-GFP as control) probed for BRD4, mH2A2 and histone H3 (loading control). Fold change quantification over wild type after H3 normalization (right). Data are mean with SE, n=3, significant p value indicated in the figure (t-test), i) Immunoblots from whole cell extracts in MCF7 clones with knockdown or over-expression of mH2A-GFP constructs (and H2A-GFP as control). Quantification of immunoblots (right), n=3 (p>0.05, t-test).

Supplementary Figure 7

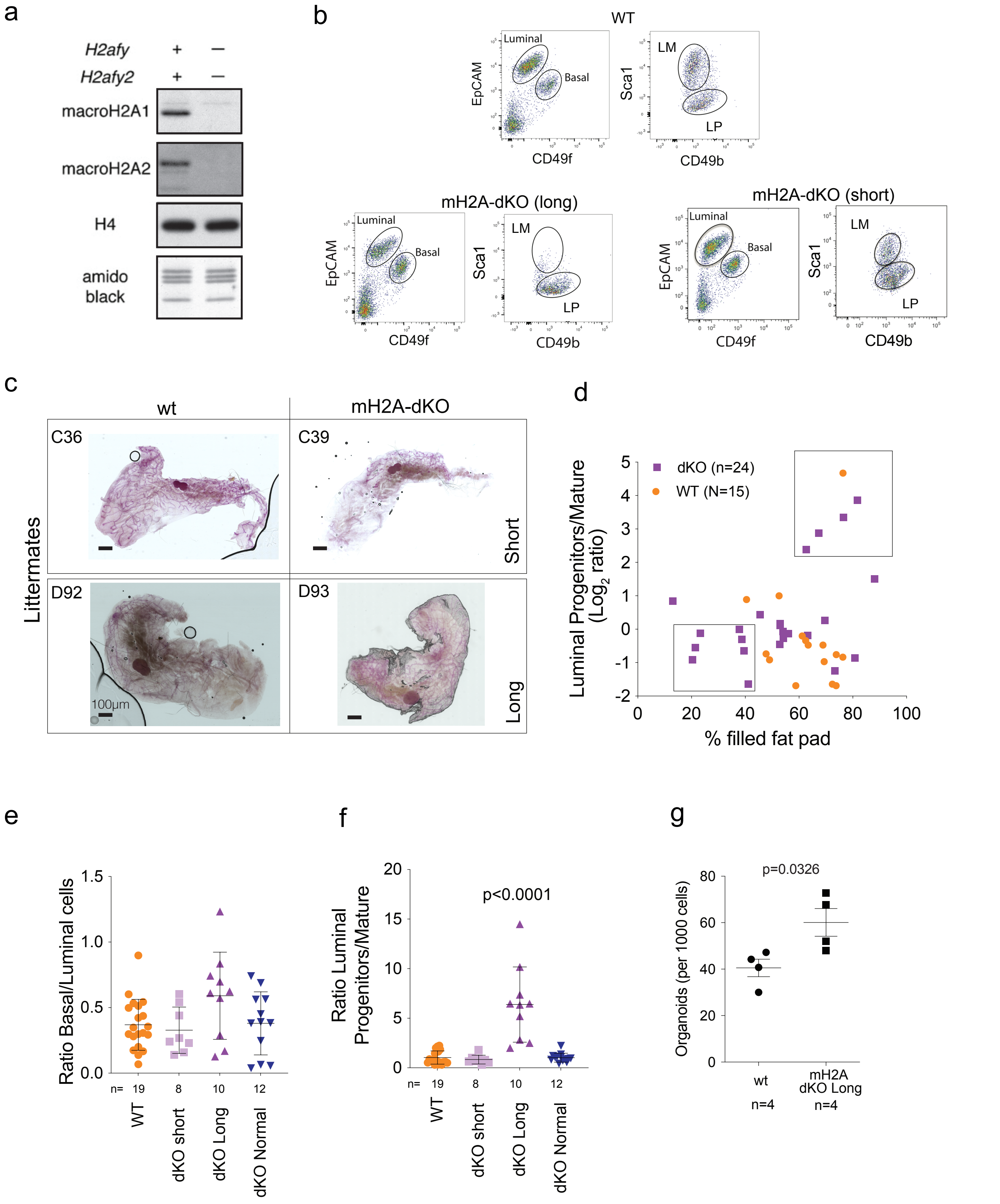

Supplemental Figure 7. Phenotypic analysis of mammary glands from mH2A double knockout mice. a) Western blot analysis of mammary epithelial cells from WT and dKO mice for expression levels of macroH2A1 (H2AFY) and macroH2A2 (H2AFY2). H4 and amido black staining of histones used as loading controls. b) EpCAM/CD49f flow cytometry plots of mammary epithelial cells (Lineage negative) with luminal and basal cell distribution in WT mammary epithelial cells. Luminal cells were further subdivided into luminal mature and luminal progenitors using Sca1/CD49b surface markers. c) Carmine stain of whole mount mammary glands from WT and mH2A dKO littermates. d) The ratio between the cell luminal mature and luminal progenitors in for WT and dKO plotted as Log2 scale against the percentage of fat pad filled, highlighting two different clusters (short and long). e) The ratio between basal and luminal cells from the mammary gland in WT and the three classes of mH2A dKO (short, long and normal). f) The ratio between luminal progenitors and mature cells from the mammary gland in WT and the three classes of mH2A dKO (short, long and normal). g) Scatter dot plot with number of organoids per 100 cells plated from wt and mH2A dKO mouse mammary epithelial cells with the long phenotype after 7 days in culture. Data are mean with SE (*p*=0.0326, unpaired t-test).

Supplementary Figure 8

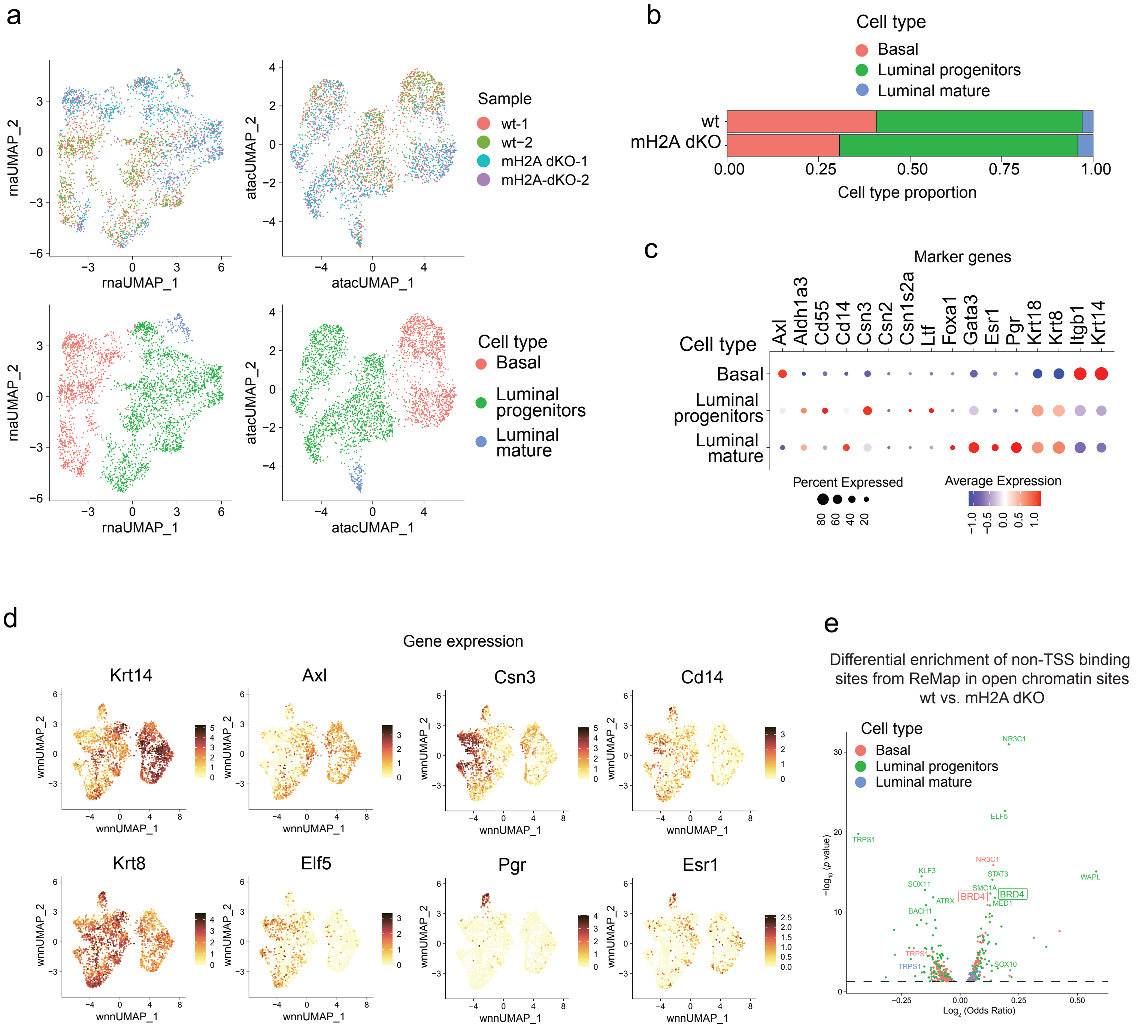

Supplemental Figure 8. Multiome analysis of mammary stem cells derived from mH2A double knockout mice. a) UMAP plots based on scRNA-seq and scATAC-seq signals from the single cell Multiome-seq assay on mammary stem cells, colored by sample (*n* = 779 each) and by cell type (Basal, *n* = 1113; Luminal progenitors, *n* = 1891; Luminal mature, *n* = 112) identified by graph-based clustering of the combined scRNA-seq and scATAC-seq signals and identifying them by their markers. b) Cell type proportions in each cluster for each genotype. c) Dot plot showing the average gene expression of marker genes for sub-populations of mammary stem cells – basal cell markers, luminal cell markers and milk proteins. d) Weighted nearest neighbor (WNN) UMAP plot - combining single cell gene expression and single cell ATAC-seq signals from the single cell Multiome-seq assay, showing expression of marker genes. e) Volcano plot showing the differential enrichment of ChIP-seq peaks of DNA binding proteins from ReMap in open chromatin regions excluding TSS, in each cell type tested using Fisher's exact test.

Supplementary Table 1- Oligos used for RTqPCR

Gene	Forward	Reverse
BRD4-short	TCCGAAACAGGTCCTGCCTA	GAGTCCTGTCCCTTTCACGG
BRD4-long	CTCCAGTTGCGCCCAAAAAG	GAAGCTGTCGCTGGATGACT
BRD4-common	AGCGCTTGGAAAACAACTATTACTG	TCCAGAGCTTCTGCCATTAAGAC
TBP	GGTTTGCTGCGGTAATCATGA	CTCCTGTGCACACCATTTTCC
RPL19	CAGATAATGGGAGGAGCCGGG	CCTGAGCATACTCATGGCTGC
Hs-mH2A1	TGGGAAGAAGAACTCCACCA	CCGTCCCTTCTTGTTGTCTC
Hs-mH2A1.1	GAAGAAGCAGGGTGAAGTCAG	GGTGAACGACAGCATCACTG
Hs-mH2A1.2	GAAGAAGCAGGGTGAAGTCAG	TGGTAGGATTGATTATGGCCTCC
Hs-mH2A2	GTTCAGCTAGGGCAGGTGTC	CAACTGCCAGCAAGATGTGT
FOSL2	CAGAAATTCCGGGTAGATATGCC	GGTATGGGTTGGACATGGAGG
RBKS	ACCTTGTTCAATCCAGCCCC	CCACCGTGAGGCCAGTTAAA
ZMYND8	GCCACGTCGAGCCACTT	CGGATAAACTCCCTGCTTGTCC
brd4	CCATGGACATGAGCACAATC	TGGAGAACATCAATCGGACA
L7	AGCGGATTGCCTTGACAGAT	AACTTGAAGGGCCACAGGAA
nanog	AGGGTCTGCTACTGAGATGCTCTG	CAACCACTGGTTTTTCTGCCACCG
mm-mH2A1.1	CGGTGGTGAAGTAGGAAACAC	GCTGCCAATGGATGGGAAG
mm-mH2A1.2	GGTGGAGGCCATAATCAATCC	GCCAAGCAGTTTTTCACCGTC
mm-mH2A2	AAGGCAAGTCAGAGACGATCC	TCTTTATCGCTGTCCTTTGCTT

Supplementary Table 2 - Antibodies used in this study

Target	Company	Catalog #	Lot #	Host	Use
H3K27me3	Millipore	07-449	3018864	Rabbit polyclonal	WB/ChIP
H3K27ac	Abcam	ab4729	GR3202987-4	Rabbit polyclonal	WB
H3K27ac	Cell signaling	8173BC	(D5E4)	Mouse monoclonal	ChIP
H3K4me1	EDL(Homemade)		1	Rabbit polyclonal	ChIP
H4K12ac	Millipore	07-595	1713305	Rabbit polyclonal	WB
P300 (Clone NM11)	Active Motif	61401	01813001	Mouse monoclonal	ChIP
BRD4	Bethyl	A301-985A50	4	Rabbit polyclonal	ChIP
BRD4	Abcam	ab128874	GR3251918-5	Rabbit monoclonal	WB
Cas9	Millipore	MAC133		Mouse monoclonal	WB
H2A.Z	Abcam	ab150402		Rabbit polyclonal	WB/ChIP
mH2A1	Abcam	ab37264	GR3219297-2	Rabbit polyclonal	ChIP
mH2A1	Millipore	07-219		Rabbit polyclonal	WB
mH2A2	Bernstein lab - Homemade		#1691	Rabbit	WB/ChIP
H3	Abcam	ab1791	GR3197444-2	Rabbit monoclonal	WB
H4	Abcam	ab177840	GR3235044-3	Rabbit monoclonal	WB
GFP	Roche	11814460001	27575600	Mouse monoclonal	WB
ERα	Santa Cruz	SC8002	B1920	Mouse monoclonal	WB
Mouse IgG DyLight 680	Invitrogen	SA5-10170	TK2670245	Donkey	WB
Rabbit IgG DyLight 800	Invitrogen	SA5-10044	TL2687937	Donkey	WB
APC - CD49b	BD Pharmigen	558295		Hamster	FACS
PerCP/Cy5.5 CD49f	Biolegend	313617		Rat	FACS
APC/Cy7 CD326 (EpCAM)	Biolegend	118217		Rat	FACS
V500 Ly6A/E - Sca1	BD horizon	561229		Rat	FACS
Anti - CD61 PE	eBioscience	12-0611-81		Hamster	FACS

Supplementary Table 3 – Source of individual datasets

Data sets	Source	Identifier/Link
HMEC, NHM and HepG2: ChIP-seq H2AZ, H2BK120ac, H2BK12ac, H2BK5ac, H3K27ac, H3K27me3, H3K36me3, H3K4me1, H3K4me3, H3K79me2, H3K9ac, H3K9me3, H4K20me1, H4K8ac and the 25-state chromatin model	Roadmap	https://egg2.wustl.edu/roadmap/web_portal/imputed.html; HMEC: E119, NHM: E059, HepG2: E118 (Imputed ChIP-seq signals)
Human Mammary Epithelial Cells (HMEC) ChIP-seq H3K4me1, H3K4me3, H3K27ac, H3K27me3, CTCF, RNA-seq (Total and PolyA)	ENCODE Phase 3 (Reference Epigenome: ENCSR460EGF)	H3K4me1 - ENCFF869PMY<br>H3K4me3 - ENCFF607JQM<br>H3K27ac - ENCFF936NNA<br>H3K27me3 - ENCFF055JAQ<br>H3K36me3 - ENCFF121IEZ<br>CTCF - ENCFF233OSO<br>RNA-seq (Total) - ENCFF499SYJ<br>RNA-seq (PolyA) - ENCFF284MYQ
HMEC ChIP-seq mH2A.1, mH2A.2, ATAC-seq	This study	
HMEC ChIP-seq H2A.z, H3K4me1 (narrowPeak), H2BK12ac, H2BK120ac, H3K36me3, DNA Methylation (5mC)	Roadmap	https://egg2.wustl.edu/roadmap/web_portal/processed_data.html#ChipSeq_DNaseSeq; E119
TCGA RNA-seq expression at enhancers in normal breast tissue	PMID: 29625054	15808_enhancer_BRCA_normal_113.tsv obtained from authors of PMID: 29625054
HMEC RNA-seq (RPKM at protein coding) for GeneHancer analysis	Roadmap	https://egg2.wustl.edu/roadmap/web_portal/processed_data.html#RNAseq_uni_proc; 57epigenomes.RPKM.pc.gz (RNA-seq RPKM at pc)
Normal Human Melanocytes (NHM) ChIP-seq H3K4me1, H3K4me3, H3K27ac, H3K27me3	ENCODE Phase 3 (Reference Epigenome: ENCSR918YMF)	H3K4me1 - ENCFF454ZRX<br>H3K4me3 - ENCFF920CYZ<br>H3K27ac - ENCFF355EPR<br>H3K27me3 - ENCFF373HCG
NHM ChIP-seq H2A.z	PMID: 26051178	GSE68222
NHM ATAC-seq, RNA-seq	PMID: 29149598	GSE94488 (Runs: ATAC-seq - SRR5228487; RNA-seq - SRR5228548)
NHM ChIP-seq mH2A.1, mH2A.2	This study	
MCF7 ChIP-seq H3K4me1, H3K4me3, H3K27ac, H3K27me3, CTCF, RNA-seq (PolyA)	ENCODE Phase 3 (Reference Epigenome: ENCSR247DVY)	H3K4me1 - ENCFF733JSN<br>H3K4me3 - ENCFF119HFM<br>H3K27ac - ENCFF997LWW<br>H3K27me3 - ENCFF346UXY<br>CTCF - ENCFF254RJD<br>RNA-seq (PolyA) - ENCFF712EIK
MCF7 ATAC-seq	PMID: 28867293	GSE101736 (Run: SRR5857006)
MCF7 H3K4me1 (peaks), H3K4me3 (peaks)	PMID: 29273624	GSE85158 (Runs: H3K4me1 (peaks) - SRR3997217; H3K4me3 (peaks) - SRR3997219)
MCF7 ChIP-seq mH2A.1, mH2A.2, scATAC-seq wt, mH2A2KO	This study	
HepG2 ChIP-seq H3K4me1, H3K4me3, H3K27ac, H3K27me3, CTCF, RNA-seq (Total and PolyA)	ENCODE Phase 3 (Reference Epigenome: ENCSR888GEN)	H3K4me1 - ENCFF900MSU<br>H3K4me3 - ENCFF554PRV<br>H3K27ac - ENCFF401PIF<br>H3K27me3 - ENCFF099LEI<br>CTCF - ENCFF989NNN<br>RNA-seq (Total) - ENCFF770XVY<br>RNA-seq (PolyA) - ENCFF989ZMQ
HepG2 ChIP-seq mH2A.1, mH2A.2	PMID: 28283545	GSE58175
MDA-MB-231L ChIP-seq mH2A.1, mH2A.2, H3K27me3, BRD4, p300, H3K4me1, H3K27ac, ATAC-seq and RNA-seq	This study	
MDA-MB-231 ChIP-seq BRD4 (short and long isoforms) (peaks)	PMID: 32446320	GSE136151
MDA-MB-231 ChIP-seq ZMYND8	PMID: 29629903	GSE108833
Dermal fibroblasts ChIP-seq H3K27ac, H3K27me3, H2A.z, mH2A.1, mH2A.2	PMID: 23463008	GSE40813

Mouse Embryonic Fibroblast (MEF) ChIP-seq H3K4me3	ENCODE Phase 3 (Reference Epigenome: ENCSR030BUT)	H3K4me3 - ENCFF581VIC
Dermal fibroblasts RNA-seq, ATAC-seq	This study	
Dermal fibroblasts OSKM binding sites in iPS reprogramming (ChIP-seq peaks)	PMID: 28111071	GSE90895
Mammary Stem Cells (MaSC) Multiome	This study	
Data used for super enhancer prediction using LILY:<br>HMEC, NHM and HepG2 H3K27ac (ChIP-seq fold change over input, narrowPeak and broadPeak)<br>MCF7 H3K27ac (genomic alignments; BAM)	HMEC, NHM and HepG2: Roadmap<br>MCF7: ENCODE Phase 3 (Reference Epigenome: ENCSR247DVY)	https://egg2.wustl.edu/roadmap/web_portal/processed_data.html#ChipSeq_DNaseSeq; HMEC: E119, NHM: E059, HepG2: E118<br>H3K27ac - ENCFF855RCK<br>Input control - ENCFF587UZE
MCF10A, MCF7, ZR751, MB361, UACC812, SKBR3, AU565, HCC1954, MB231, MB436, MB468, HCC1937 – ChIP-seq H3K27ac	PMID: 29273624	GSE85158
Breast Cancer genetic variants from GWAS	PMID: 29059683	https://bcac.ccge.medschl.cam.ac.uk/bcacdata/oncoarray/oncoarray-and-combined-summary-result/gwas-summary-results-breast-cancer-risk-2017
Mammary Epithelial (HME1), Melanocytes (HMEL) and MCF7 - ChIP-seq H4K12ac	PMID: 31433991 (HME1), 28445736 (HMEL), 25788266 and 25017071 (MCF7)	GSE133728 (HME1)<br>GSE58953 (HMEL)<br>GSE65886 (MCF7 Veh)<br>GSE55921 (MCF7 Veh - Input)
Conservation		http://hgdownload.cse.ucsc.edu/goldenpath/hg19/phastCons100way/
ReMap	PMID: 29126285	ReMap2022: http://remap.univ-amu.fr/
TSS annotations		https://github.com/buenrostrolab/tss-annotation

Supplementary Table 4 – List of Software and Databases used

Tool	Reference
**SOFTWARES**	
TrimGalore v0.4.5	https://github.com/FelixKrueger/TrimGalore
bowtie2 v2.3.3.1	Langmead B, Salzberg S. Fast gapped-read alignment with Bowtie 2. Nature Methods. 2012, 9:357-359.
Samtools v1.9	Twelve years of SAMtools and BCFtools<br>Petr Danecek, James K Bonfield, Jennifer Liddle, John Marshall, Valeriu Ohan, Martin O Pollard, Andrew Whitwham, Thomas Keane, Shane A McCarthy, Robert M Davies, Heng Li<br>*GigaScience*, Volume 10, Issue 2, February 2021
Picard v2.9.0	"Picard Toolkit." 2019. Broad Institute, GitHub Repository. https://broadinstitute.github.io/picard/; Broad Institute
Macs2 v2.2.7.1	Zhang, Y., Liu, T., Meyer, C.A. et al. Model-based Analysis of ChIP-Seq (MACS). Genome Biol 9, R137 (2008)
ataqv v1.3.0	Orchard, P., Kyono, Y., Hensley, J., Kitzman, J.O. & Parker, S.C.J. Quantification, Dynamic Visualization, and Validation of Bias in ATAC-Seq Data with ataqv. Cell Syst 10, 298-306 e4 (2020).
UCSC Genome Browser	Kent WJ, Sugnet CW, Furey TS, Roskin KM, Pringle TH, Zahler AM, Haussler D. The human genome browser at UCSC. Genome Res. 2002 Jun;12(6):996-1006.
UCSC Genome Browser Tools	Kent WJ, Zweig AS, Barber G, Hinrichs AS, Karolchik D. BigWig and BigBed: enabling browsing of large distributed data sets. Bioinformatics. 2010 Sep 1;26(17):2204-7.
DeepTools v3.5.0	Ramírez, Fidel, Devon P. Ryan, Björn Grüning, Vivek Bhardwaj, Fabian Kilpert, Andreas S. Richter, Steffen Heyne, Friederike Dündar, and Thomas Manke. "deepTools2: a next generation web server for deep-sequencing data analysis." Nucleic Acids Research (2016): gkw257.
Bedtools v2.27.1	Quinlan AR, Hall IM. BEDTools: a flexible suite of utilities for comparing genomic features. Bioinformatics. 2010 Mar 15;26(6):841-2.
edgeR v3.36.0	Robinson MD, McCarthy DJ, Smyth GK. edgeR: a Bioconductor package for differential expression analysis of digital gene expression data. Bioinformatics. 2010;26(1):139-140.
STAR v2.7.3a	Dobin A, Davis CA, Schlesinger F, Drenkow J, Zaleski C, Jha S, Batut P, Chaisson M, Gingeras TR. STAR: ultrafast universal RNA-seq aligner. Bioinformatics. 2013 Jan 1;29(1):15-21.
Salmon v1.5.2	Patro, R., Duggal, G., Love, M. I., Irizarry, R. A., & Kingsford, C. (2017). Salmon provides fast and bias-aware quantification of transcript expression. Nature Methods.
tximport v1.22.0	Soneson C, Love MI, Robinson MD (2015). "Differential analyses for RNA-seq: transcript-level estimates improve gene-level inferences." F1000Research
10x Genomics Cell Ranger ATAC v2.0.0	Satpathy, Ansuman T. et al, Massively parallel single-cell chromatin landscapes of human immune cell development and intratumoral T cell exhaustion. Nature Biotechnology. 37: 925-936
Seurat v4.0.4	Hao and Hao et al. Integrated analysis of multimodal single-cell data. Cell (2021)
Signac v1.4.0	Stuart et al. Single-cell chromatin state analysis with Signac. Nature Methods (2021).
Cicero v1.3.5	Pliner HA, Packer JS, McFaline-Figueroa JL, Cusanovich DA, Daza RM, Aghamirzaie D, Srivatsan S, Qiu X, Jackson D, Minkina A, Adey AC, Steemers FJ, Shendure J, Trapnell C. Cicero Predicts cis-Regulatory DNA Interactions from Single-Cell Chromatin Accessibility Data. Mol Cell. 2018 Sep 6;71(5):858-871.e8.
Circlize v0.4.14	Gu Z, Gu L, Eils R, Schlesner M, Brors B (2014). "circlize implements and enhances circular visualization in R." Bioinformatics, 30, 2811-2812.

gprofiler2	Kolberg, L., Raudvere, U., Kuzmin, I., Vilo, J. and Peterson, H., 2020. gprofiler2–an R package for gene list functional enrichment analysis and namespace conversion toolset g: Profiler. F1000Research, 9(ELIXIR):709.
ChromHMM v1.23	Ernst J, Kellis M. Chromatin-state discovery and genome annotation with ChromHMM. Nature Protocols, 12:2478-2492, 2017.
HOMER v4.11	Heinz S, Benner C, Spann N, Bertolino E et al. Simple Combinations of Lineage-Determining Transcription Factors Prime cis-Regulatory Elements Required for Macrophage and B Cell Identities. Mol Cell 2010 May 28;38(4):576-589.
HMCan v1.41	"HMCan: a method for detecting chromatin modifications in cancer samples using ChIP-seq data" Haitham Ashoor; Aurelie Herault; Aurelie Kamoun; Francois Radvanyi; Vladimir B. Bajic; Emmanuel Barillot; Valentina Boeva. Bioinformatics, 2013
LILY	Boeva, V., Louis-Brennetot, C., Peltier, A. et al. Heterogeneity of neuroblastoma cell identity defined by transcriptional circuitries. Nat Genet 49, 1408–1413 (2017).
Intervene v0.6.5	Khan A, Mathelier A. Intervene: a tool for intersection and visualization of multiple gene or genomic region sets. BMC Bioinformatics. 2017;18:287. doi: 10.1186/s12859-017-1708-7.
Cistrome-GO	Li S, Wan C, Zheng R, et al. Cistrome-GO: a web server for functional enrichment analysis of transcription factor ChIP-seq peaks[J]. Nucleic acids research, 2019.
GREAT v4.0.4	McLean, C., Bristor, D., Hiller, M. et al. GREAT improves functional interpretation of cis-regulatory regions. Nat Biotechnol 28, 495–501 (2010).
ReMapEnrich v0.99.0	https://github.com/remap-cisreg/ReMapEnrich
GAT v1.3.4	Heger A, Webber C, Goodson M, Ponting CP, Lunter G. GAT: a simulation framework for testing the association of genomic intervals. Bioinformatics. 2013 Aug 15;29(16):2046-8.
GARFIELD v2	Iotchkova V, Ritchie GRS, Geihs M, Morganella S, Min JL, Walter K, Timpson NJ; UK10K Consortium, Dunham I, Birney E, Soranzo N. GARFIELD classifies disease-relevant genomic features through integration of functional annotations with association signals. Nat Genet. 2019 Feb;51(2):343-353.
CrossMap v0.5.2	Zhao H, Sun Z, Wang J, Huang H, Kocher JP, Wang L. CrossMap: a versatile tool for coordinate conversion between genome assemblies. Bioinformatics. 2014;30(7):1006-1007.
**DATABASES**	
GeneHancer v4.4	Fishilevich S, Nudel R, Rappaport N, et al. GeneHancer: genome-wide integration of enhancers and target genes in GeneCards. Database (Oxford). 2017.
Ensembl grch37/release-104	Fiona Cunningham et al. Ensembl 2022, Nucleic Acids Research, Volume 50, Issue D1, 7 January 2022, Pages D988–D995
Roadmap Epigenomics Project	Roadmap Epigenomics Consortium., Kundaje, A., Meuleman, W. et al. Integrative analysis of 111 reference human epigenomes. Nature 518, 317–330 (2015).
ENCODE cCRE Registry V3	The ENCODE Project Consortium., Moore, J.E., Purcaro, M.J. et al. Expanded encyclopaedias of DNA elements in the human and mouse genomes. Nature 583, 699–710 (2020).
ENCODE blacklist v2	Amemiya, H.M., Kundaje, A. & Boyle, A.P. The ENCODE Blacklist: Identification of Problematic Regions of the Genome. Sci Rep 9, 9354 (2019).
UCSC Table Browser	Karolchik D, Hinrichs AS, Furey TS, Roskin KM, Sugnet CW, Haussler D, Kent WJ. The UCSC Table Browser data retrieval tool. Nucleic Acids Res. 2004 Jan 1;32(Database issue):D493-6.
ReMap 2022	ReMap 2022: a database of Human, Mouse, Drosophila and Arabidopsis regulatory regions from an integrative analysis of DNA-binding sequencing experiments. Fayrouz Hammal, Pierre De Langen, Aurélie Bergon, Fabrice Lopez, Benoit Ballester. Nucleic Acids Research, 2021 Nov 9;gkab996
EMBL-EBI GWAS Catalog 2021-06-14	Buniello A, et al. The NHGRI-EBI GWAS Catalog of published genome-wide association studies, targeted arrays and summary statistics 2019. Nucleic Acids Research, 2019
TSS annotations	https://github.com/buenrostrolab/tss-annotation
