# Supplementary material for: MacroH2A histone variants modulate enhancer activity to repress oncogenic programs and cellular reprogramming
Source: Commun Biol. 2023 Feb 23;6:215. doi: 10.1038/s42003-023-04571-1 (PMC9950461; doi:10.1038/s42003-023-04571-1)
Supplement: Supplementary file 2 — Description of Additional Supplementary Files [file 42003_2023_4571_MOESM2_ESM.pdf]

Description of Additional Supplementary Files

File name: **Supplementary_Data_1.xlsx**
Description: Results of Gene Ontology (GO) analysis of genes whose promoters gain interactions with CRE in MCF7 mH2A2 knockout from single cell ATAC-seq data analysis.

File name: **Supplementary_Data_2.xlsx**
Description: Differentially expressed genes comparing macroH2A dKO (avg_log2FC > 0) and wt (avg_log2FC < 0) mammary stem cells.

File name: **Supplementary_Data_3.xlsx**
Description: QC statistics for sequencing experiments

File name: **Supplementary_Data_4.xlsx**
Description: Source data for graphs and charts (except for Fig. 1e, Fig. 7e, Supp. Fig. 1c-d, Supp. Fig. 3a and Supp. Fig. 6b)

File name: **Supplementary_Data_5.zip**
Description: Source data for Fig. 1e - ATAC-seq signal scores, and ChIP-seq signal scores of histone marks and variants around open chromatin regions grouped by the five CRE classes in HMEC

File name: **Supplementary_Data_6.txt**
Description: Source data for Fig. 7e - Number of interactions per open chromatin region in wt and mH2AdKO mammary stem cells

File name: **Supplementary_Data_7.txt**
Description: Source data for Supp. Fig. 1c - Z scores of the log-normalized input-corrected ChIP-seq signals of the 8 histone marks/variants used to classify cell-specific CRE in 4 cell types

File name: **Supplementary_Data_8.zip**
Description: Source data for Supp. Fig. 1d - Signal profile of Conservation (phastCons scores for Human, hg19, from UCSC) and Methylation signal (from RoadMap reference epigenome E119) around open chromatin regions grouped by the five CRE classes

File name: **Supplementary_Data_9.txt**
Description: Source data for Supp. Fig. 3a - Average input-corrected ChIP-Seq signals of macroH2A variants and H3K27me3 at the binding sites of Oct4, Sox2, Klf4 and cMyc (48hrs after OSKM induction) and at active TSS regions in dermal fibroblasts

File name: **Supplementary_Data_10.zip**
Description: Source data for Supp. Fig. 6b - ChIP-seq signal scores of BRD4 around open chromatin regions, grouped by BRD4 Loss, BRD4 Neutral and BRD4 Gain regions in MDA-MB-213L control vs. mH2A2 over-expression cells

File name: **Supplementary_Data_11.pdf**
Description: Uncropped western blot images
