# Supplementary material for: MacroH2A histone variants modulate enhancer activity to repress oncogenic programs and cellular reprogramming
Source: Commun Biol. 2023 Feb 23;6:215. doi: 10.1038/s42003-023-04571-1 (PMC9950461; doi:10.1038/s42003-023-04571-1)

Figure 4a

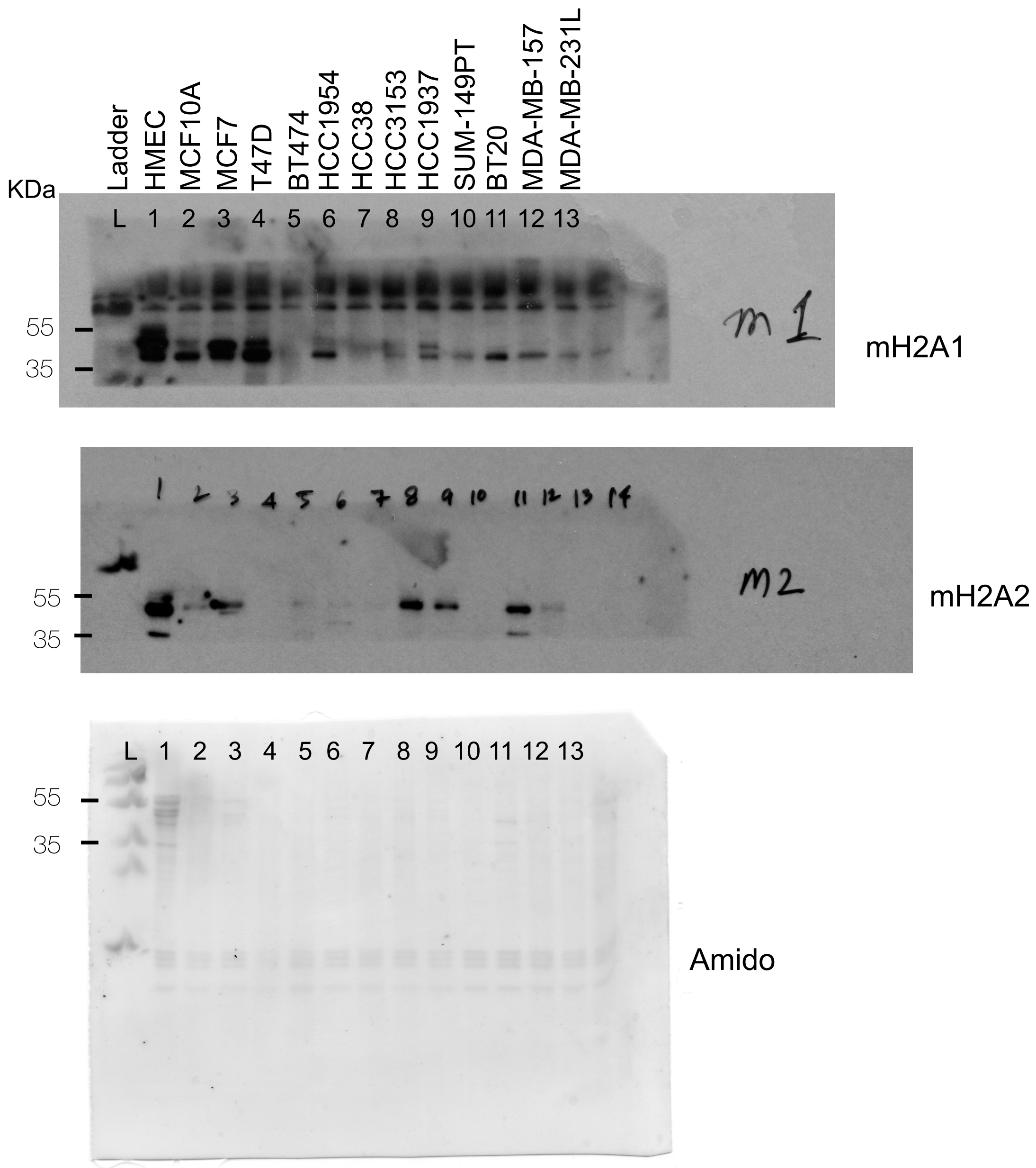

Ladder
HMEC
MCF10A
MCF7
T47D
BT474
HCC1954
HCC38
HCC3153
HCC1937
SUM-149PT
BT20
MDA-MB-157
MDA-MB-231L
KDa
L 1 2 3 4 5 6 7 8 9 10 11 12 13
55
35
m1
mH2A1
1 2 3 4 5 6 7 8 9 10 11 12 13 14
55
35
m2
mH2A2
L 1 2 3 4 5 6 7 8 9 10 11 12 13
55
35
Amido

Figure 4e

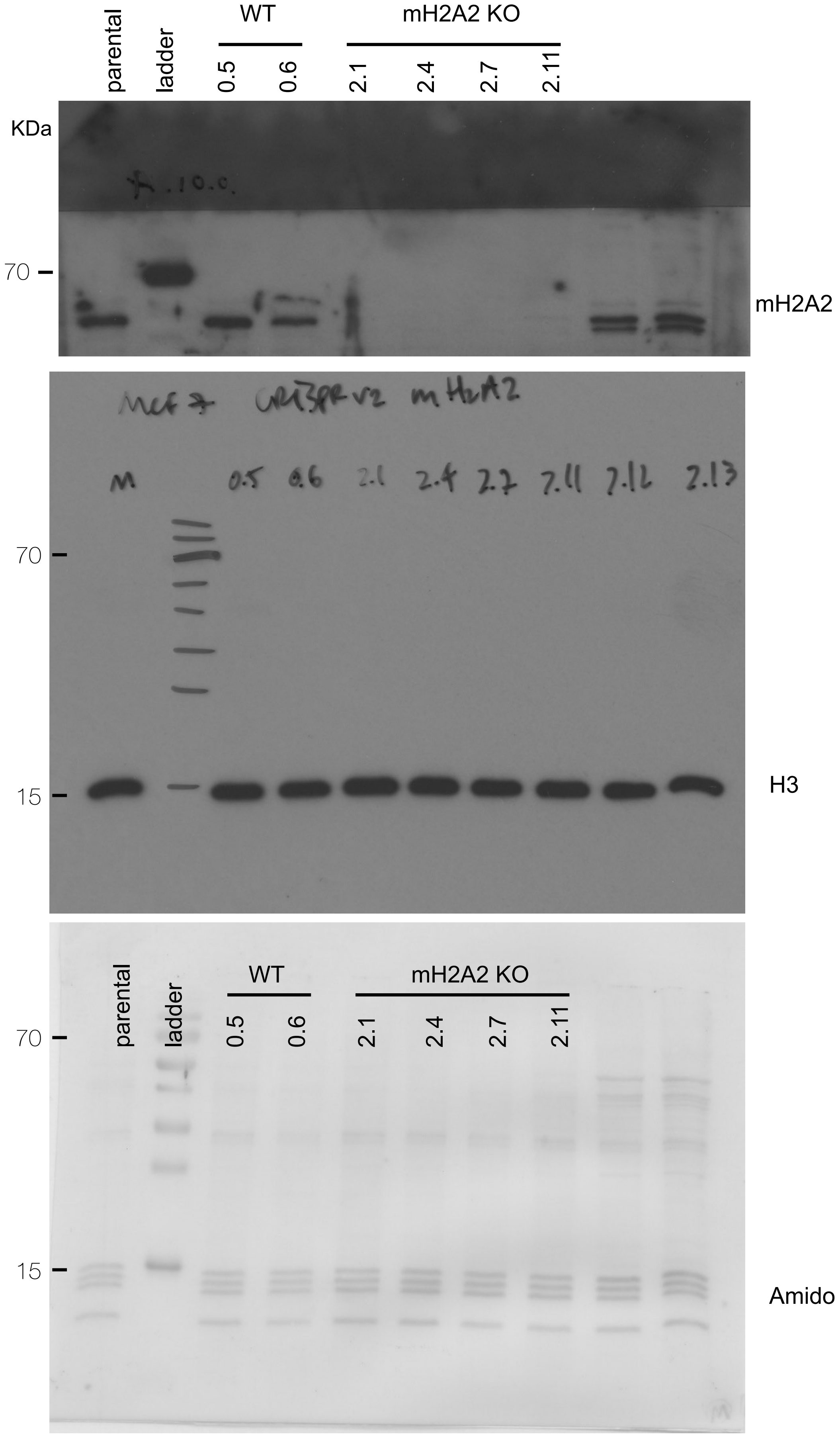
parental
ladder
WT
0.5
0.6
mH2A2 KO
2.1
2.4
2.7
2.11
KDa
70
mH2A2
70
15
H3
parental
ladder
WT
0.5
0.6
mH2A2 KO
2.1
2.4
2.7
2.11
70
15
Amido

Figure 6e

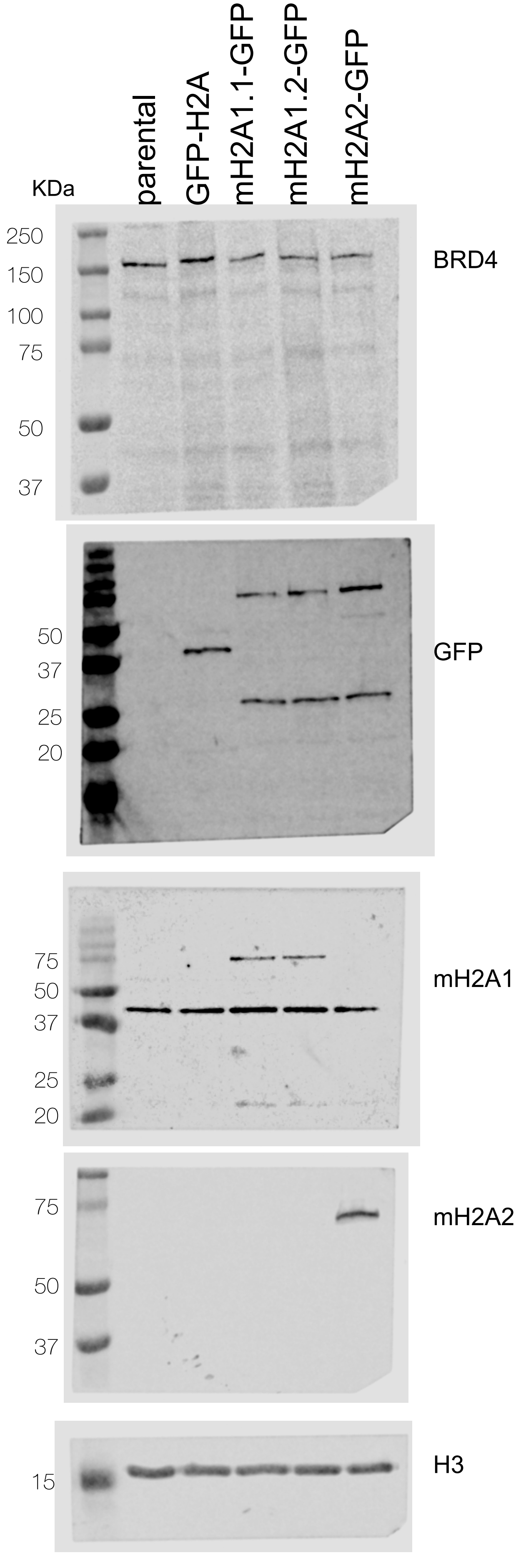

parental
GFP-H2A
mH2A1.1-GFP
mH2A1.2-GFP
mH2A2-GFP
KDa
250
150
100
75
50
37
BRD4
50
37
25
20
GFP
75
50
37
25
20
mH2A1
75
50
37
mH2A2
15
H3

Figure 6f

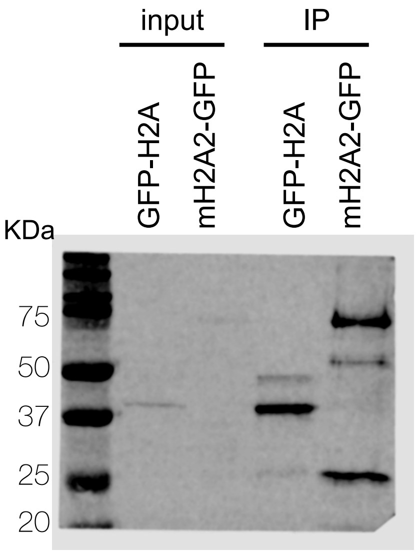

Longer exposure

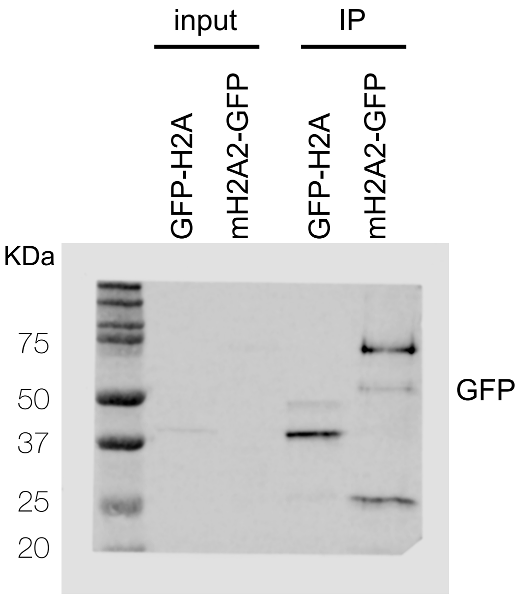

Shorter exposure

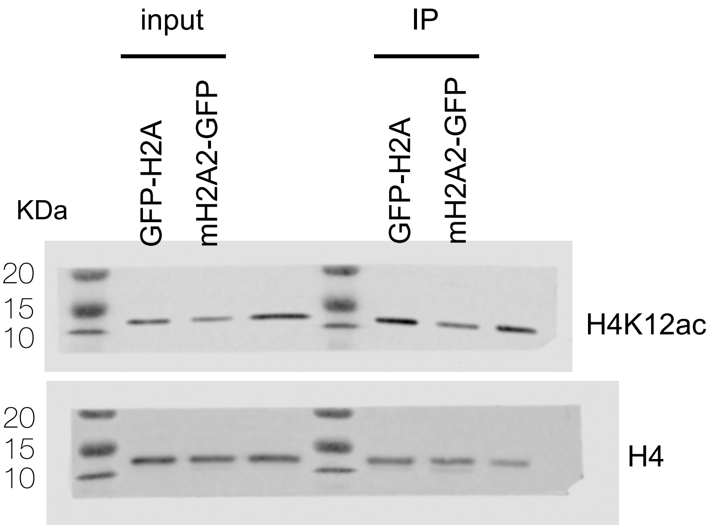

Supp. Figure 3d

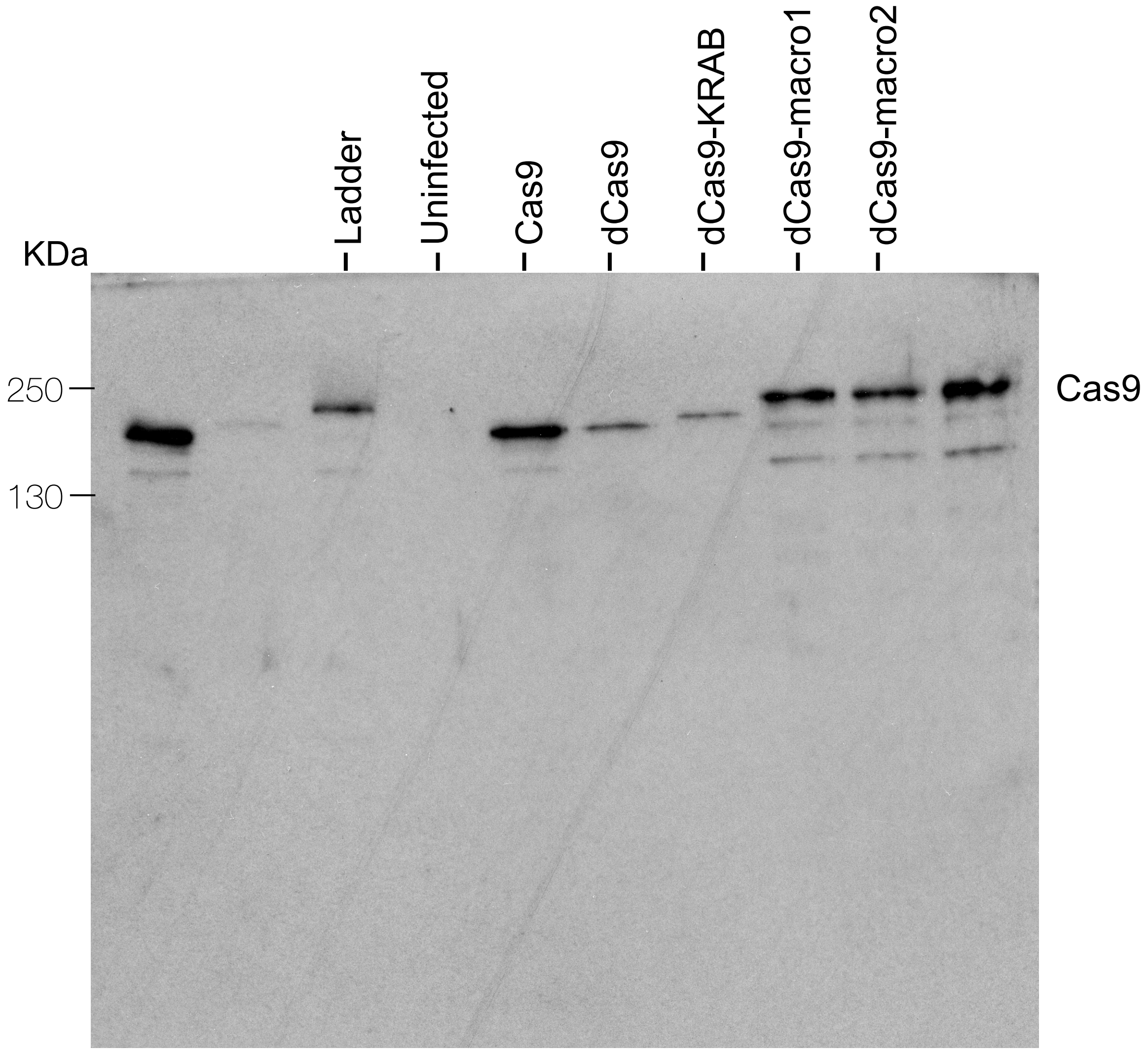

Supp. Figure 4b

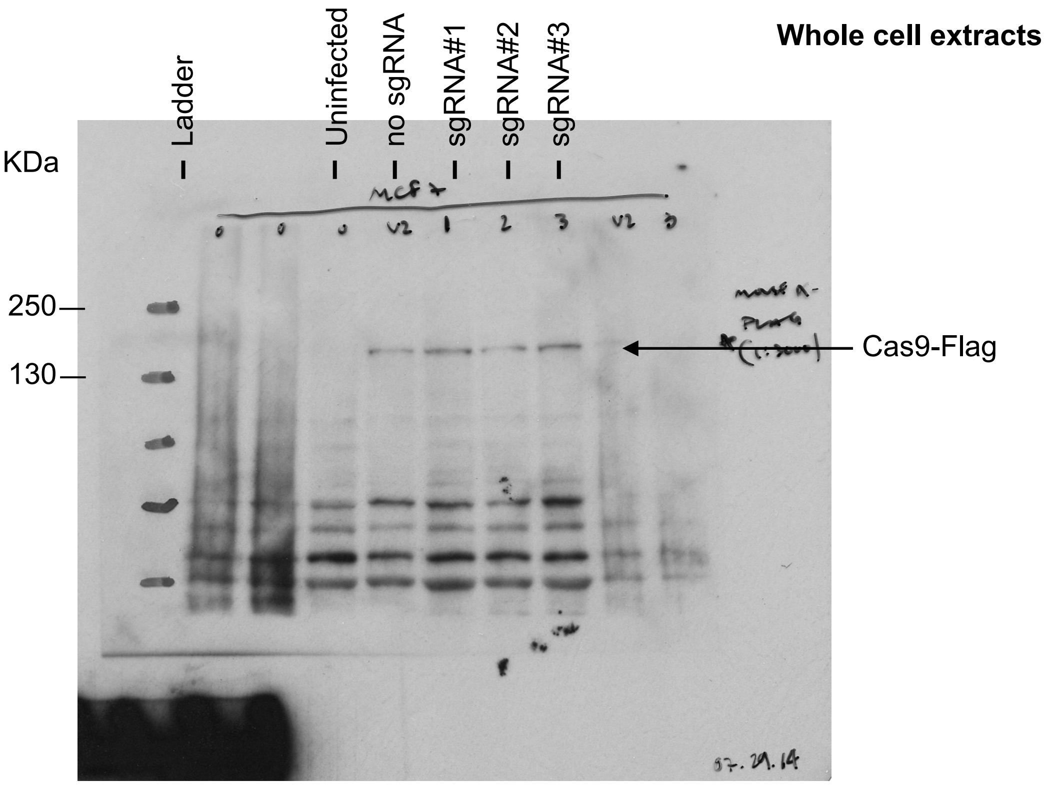

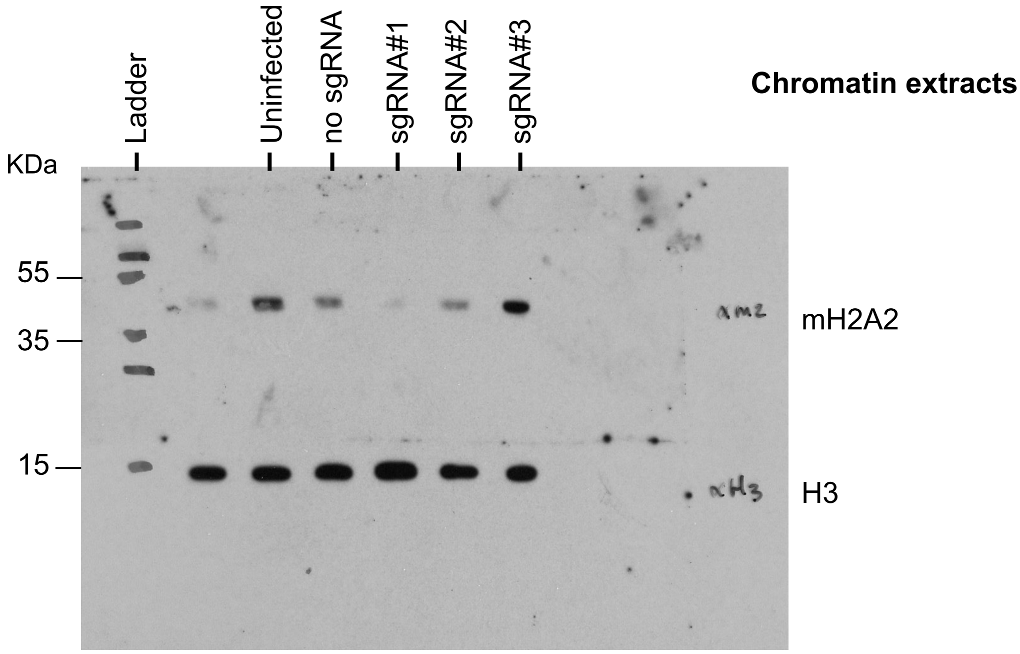

Supp. Figure 4f

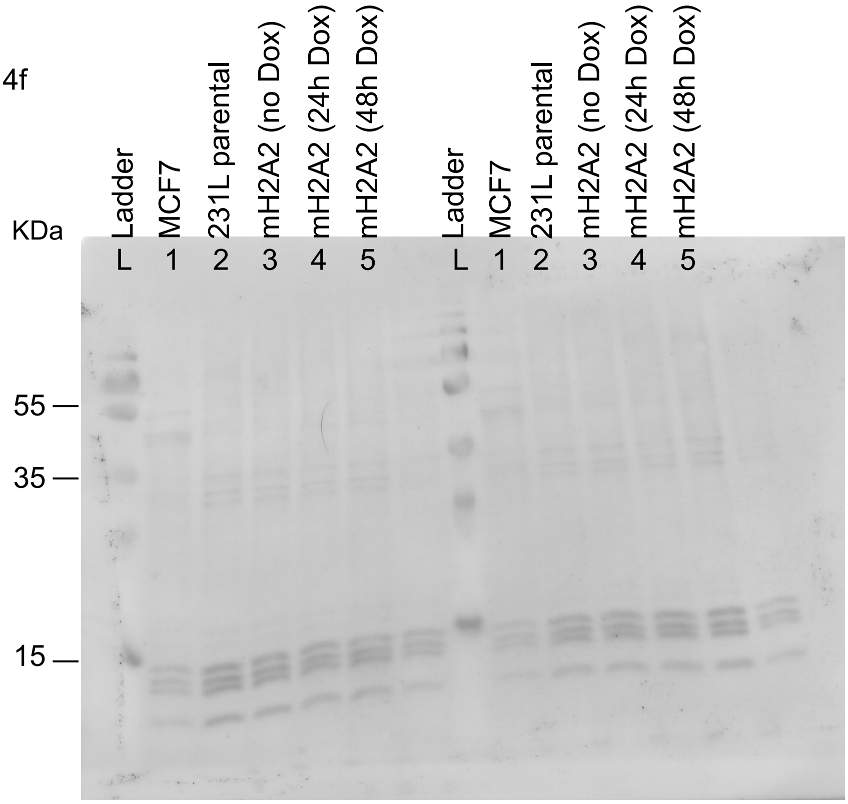

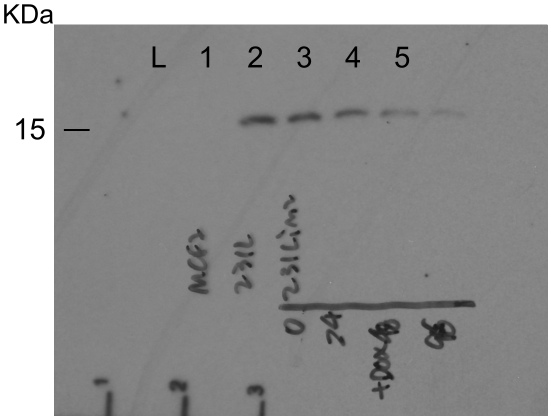

H3K27ac

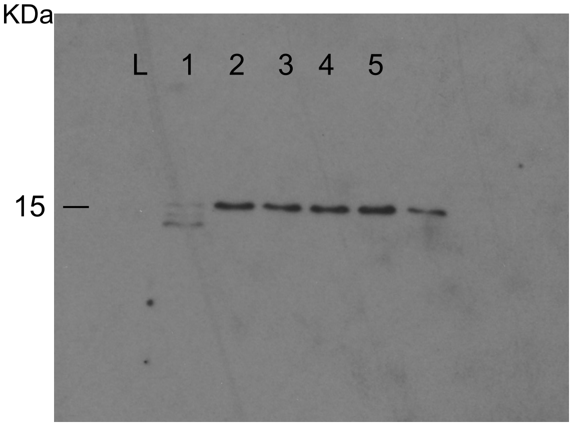

H3K27me3

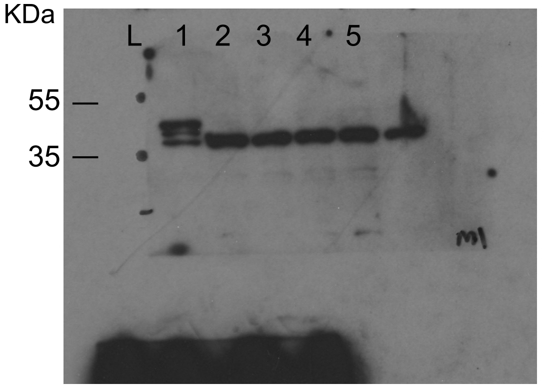

mH2A1

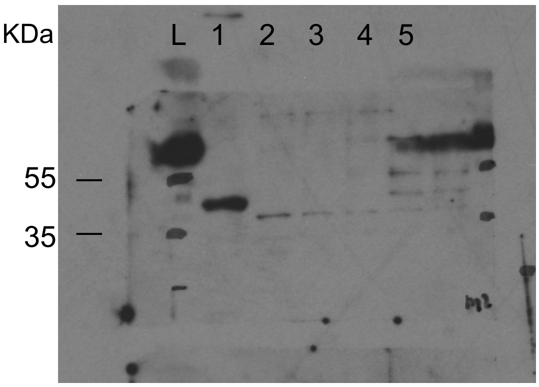

mH2A2

Supp. Figure 6f

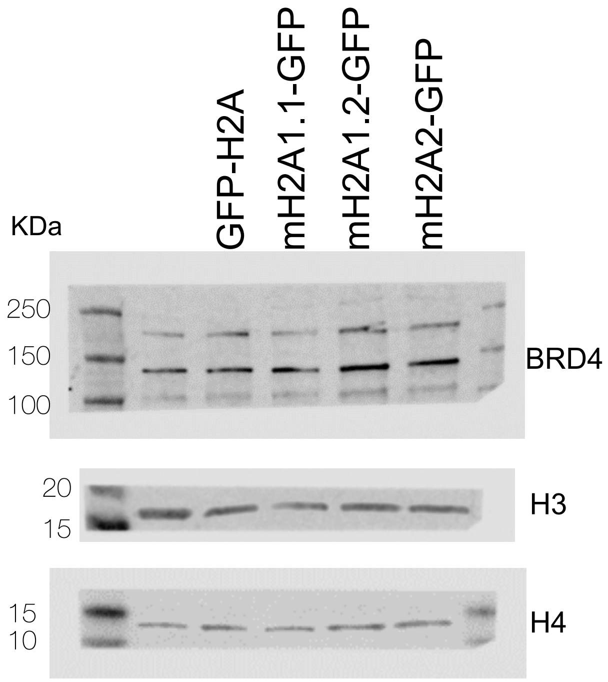

Supp. Figure 6h

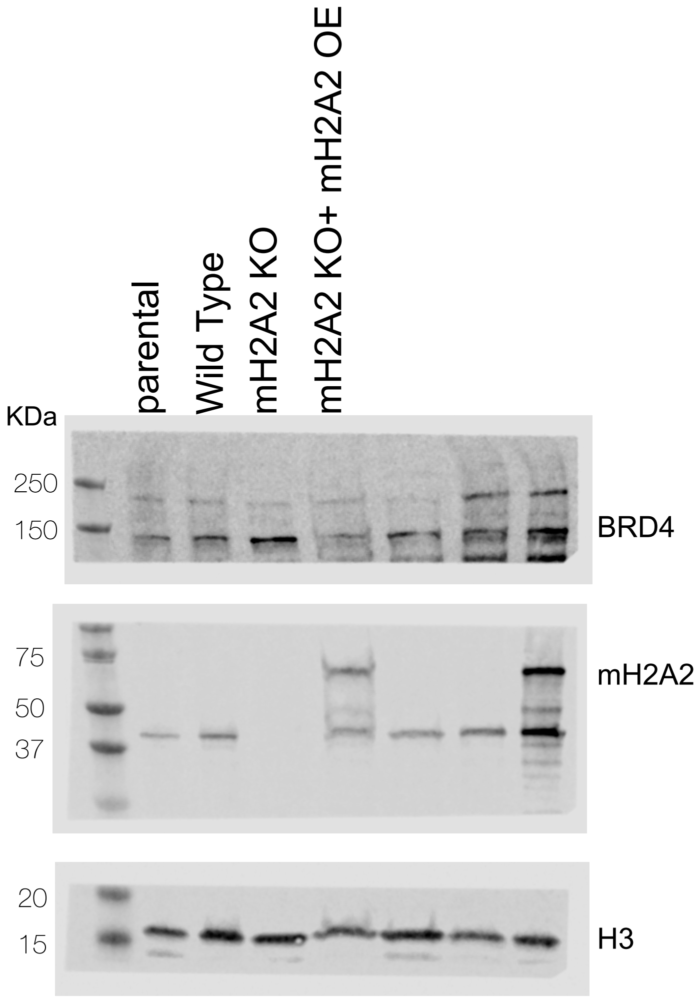

Supp. Figure 6i

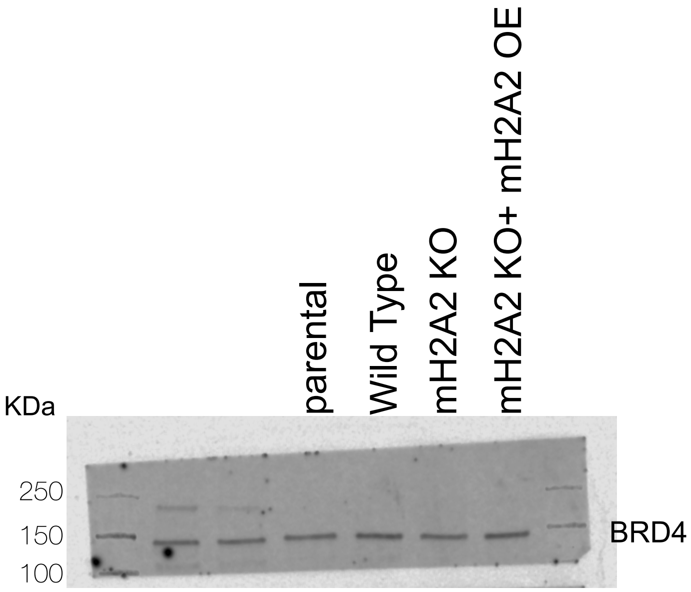

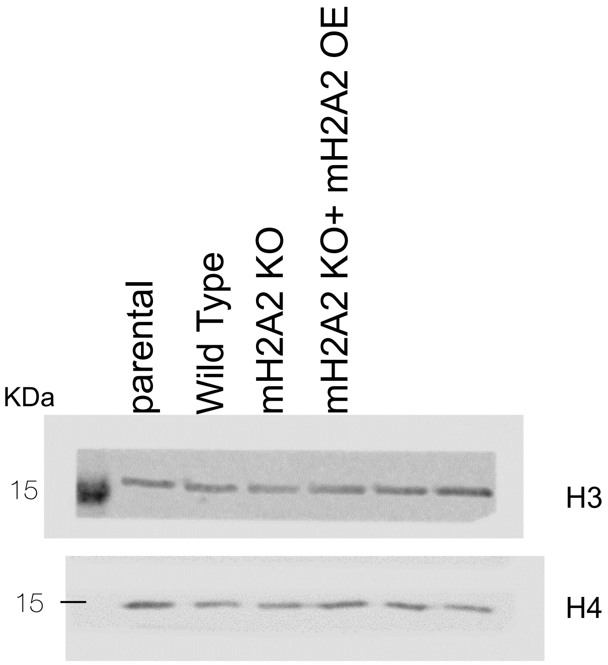

Supp. Figure 7a

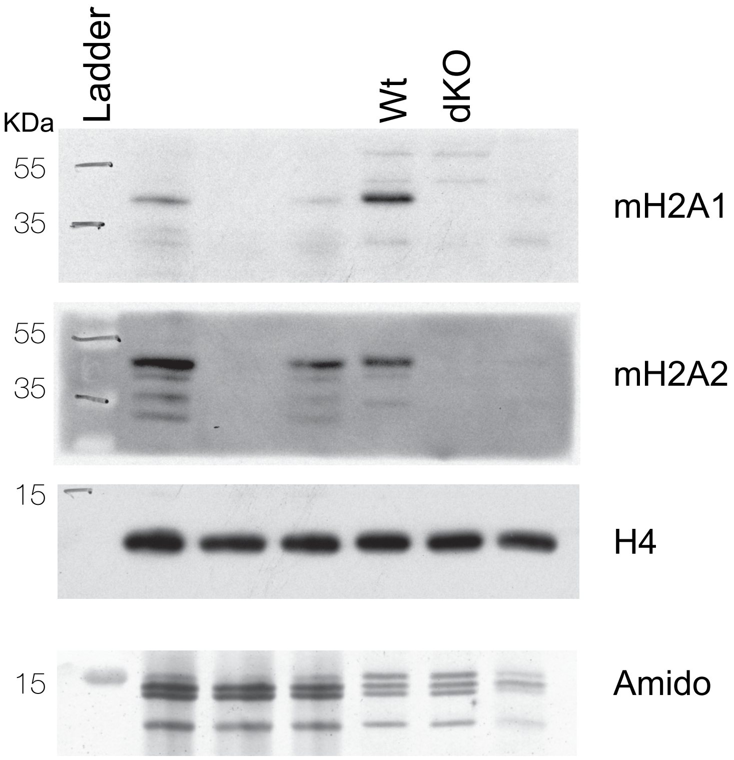

Supplement: Supplementary file 13 — Supplementary_Data_11 [file 42003_2023_4571_MOESM13_ESM.pdf]
