# Supplementary material for: MacroH2A histone variants modulate enhancer activity to repress oncogenic programs and cellular reprogramming
Source: Commun Biol. 2023 Feb 23;6:215. doi: 10.1038/s42003-023-04571-1 (PMC9950461; doi:10.1038/s42003-023-04571-1)
Supplement: Supplementary file 14 — Reporting Summary [file 42003_2023_4571_MOESM14_ESM.pdf]

nature research

Corresponding author(s): Alexandre Gaspar-Maia

Last updated by author(s): Jan 16, 2023

Reporting Summary

Nature Research wishes to improve the reproducibility of the work that we publish. This form provides structure for consistency and transparency in reporting. For further information on Nature Research policies, see our Editorial Policies and the Editorial Policy Checklist.

Statistics

For all statistical analyses, confirm that the following items are present in the figure legend, table legend, main text, or Methods section.

n/a	Confirmed	
☐	☒	The exact sample size (*n*) for each experimental group/condition, given as a discrete number and unit of measurement
☐	☒	A statement on whether measurements were taken from distinct samples or whether the same sample was measured repeatedly
☐	☒	The statistical test(s) used AND whether they are one- or two-sided<br>*Only common tests should be described solely by name; describe more complex techniques in the Methods section.*
☐	☒	A description of all covariates tested
☐	☒	A description of any assumptions or corrections, such as tests of normality and adjustment for multiple comparisons
☐	☒	A full description of the statistical parameters including central tendency (e.g. means) or other basic estimates (e.g. regression coefficient) AND variation (e.g. standard deviation) or associated estimates of uncertainty (e.g. confidence intervals)
☐	☒	For null hypothesis testing, the test statistic (e.g. *F*, *t*, *r*) with confidence intervals, effect sizes, degrees of freedom and *P* value noted<br>*Give P values as exact values whenever suitable.*
☒	☐	For Bayesian analysis, information on the choice of priors and Markov chain Monte Carlo settings
☒	☐	For hierarchical and complex designs, identification of the appropriate level for tests and full reporting of outcomes
☐	☒	Estimates of effect sizes (e.g. Cohen's *d*, Pearson's *r*), indicating how they were calculated

Our web collection on statistics for biologists contains articles on many of the points above.

Software and code

Policy information about availability of computer code

Data collection	Incucyte S3 (Sartorius), Lightcycler v4.05 (Roche), Image Lab v6.1.0 (BioRad), Image Studio v5.0 (LiCor), FlowJo v8.7
Data analysis	Custom algorithm will be available at https://github.com/LabFunEpi/mBE<br>The following software were used: TrimGalore v0.4.5<br>bowtie2 v2.3.3.1<br>Samtools v1.9<br>Picard v2.9.0<br>Macs2 v2.2.7.1<br>ataqv v1.3.0<br>UCSC Genome Browser<br>UCSC Genome Browser Tools<br>DeepTools v3.5.0<br>Bedtools v2.27.1<br>edgeR v3.36.0<br>STAR v2.7.3a<br>Salmon v1.5.2<br>tximport v1.22.0<br>10x Genomics Cell Ranger ATAC v2.0.0<br>Seurat v4.0.4<br>Signac v1.4.0<br>Cicero v1.3.5<br>Circlize v0.4.14<br>gprofiler2

ChromHMM v1.23
HOMER v4.11
HMCan v1.41
LILY
Intervene v0.6.5
Cistrome-GO
GREAT v4.0.4
ReMapEnrich v0.99.0
GAT v1.3.4
GARFIELD v2
CrossMap v0.5.2
GeneHancer v4.4
Ensembl grch37/release-104
Roadmap Epigenomics Project
ENCODE cCRE Registry V3
ENCODE blacklist v2
UCSC Table Browser
ReMap 2022
EMBL-EBI GWAS Catalog 2021-06-14
TSS annotations

For manuscripts utilizing custom algorithms or software that are central to the research but not yet described in published literature, software must be made available to editors and reviewers. We strongly encourage code deposition in a community repository (e.g. GitHub). See the Nature Research guidelines for submitting code & software for further information.

Data

Policy information about availability of data

All manuscripts must include a data availability statement. This statement should provide the following information, where applicable:

- Accession codes, unique identifiers, or web links for publicly available datasets
- A list of figures that have associated raw data
- A description of any restrictions on data availability

Data is available at the Gene Expression Omnibus database (Accession number: GSE171599)

Field-specific reporting

Please select the one below that is the best fit for your research. If you are not sure, read the appropriate sections before making your selection.

☒ Life sciences ☐ Behavioural & social sciences ☐ Ecological, evolutionary & environmental sciences

For a reference copy of the document with all sections, see nature.com/documents/nr-reporting-summary-flat.pdf

Life sciences study design

All studies must disclose on these points even when the disclosure is negative.

Sample size	Functional assays were performed at least in triplicates with similar results.
Data exclusions	No data was excluded from this study
Replication	All attempts to replicate this study were successful.
Randomization	For single cell studies, random selection of cells was used to validate the published analysis with no significant differences.
Blinding	Acquisition and quantification of MCF7 spheroids upon E2 treatment was done blindly.

Reporting for specific materials, systems and methods

We require information from authors about some types of materials, experimental systems and methods used in many studies. Here, indicate whether each material, system or method listed is relevant to your study. If you are not sure if a list item applies to your research, read the appropriate section before selecting a response.

Materials & experimental systems

n/a	Involved in the study	
☐	☒	Antibodies
☐	☒	Eukaryotic cell lines
☒	☐	Palaeontology and archaeology
☐	☒	Animals and other organisms
☒	☐	Human research participants
☒	☐	Clinical data
☒	☐	Dual use research of concern

Methods

n/a	Involved in the study	
☐	☒	ChIP-seq
☐	☒	Flow cytometry
☒	☐	MRI-based neuroimaging

Antibodies

Antibodies used

H3K27me3,Millipore,07-449,3018864
H3K27ac,Abcam,ab4729,GR3202987-4
H3K27ac,Cell signaling,8173BC,(D5E4)
H3K4me1,EDL(Homemade),1
H4K12ac,Millipore,07-595,1713305
P300 (Clone NM11),Active Motif,61401,1813001
BRD4,Bethyl,A301-985A50,4
BRD4,Abcam,ab128874,GR3251918-5
Cas9 ,Millipore,MAC133
H2A.Z,Abcam,ab150402
mH2A1,Abcam,ab37264,GR3219297-2
mH2A1,Millipore,07-219
mH2A2,Bernstein lab - Homemade,#1691
H3,Abcam,ab1791,GR3197444-2
H4,Abcam,ab177840,GR3235044-3
GFP,Roche,11814460001,27575600
ERα,Santa Cruz,SC8002,B1920
Mouse IgG DyLight 680,Invitrogen,SA5-10170,TK2670245
Rabbit IgG DyLight 800,Invitrogen,SA5-10044,TL2687937
APC - CD49b,BD Pharmigen,558295
PerCP/Cy5.5 CD49f,Biolegend,313617
APC/Cy7 CD326 (EpCAM),Biolegend,118217
V500 Ly6A/E - Sca1,BD horizon,561229
Anti - CD61 PE,eBioscience,12-0611-81

Validation

All antibodies used have previously been described in other publications.

Eukaryotic cell lines

Policy information about cell lines

Cell line source(s)

ATCC, Caliper (MDA-MB-231-luc-D3H2LN), NG4 (Christopher Schaniel).

Authentication

None of the cell lines were authenticated

Mycoplasma contamination

All the cell lines tested negative for Mycoplasma.

Commonly misidentified lines
(See ICLAC register)

None

Animals and other organisms

Policy information about studies involving animals; ARRIVE guidelines recommended for reporting animal research

Laboratory animals

Mus musculus

Wild animals

N/A

Field-collected samples

N/A

Ethics oversight

All animal experiments received prior approval from the ISMMS IACUC (Protocol # IACUC-2014-0093).

Note that full information on the approval of the study protocol must also be provided in the manuscript.

ChIP-seq

Data deposition

☒ Confirm that both raw and final processed data have been deposited in a public database such as GEO.

☒ Confirm that you have deposited or provided access to graph files (e.g. BED files) for the called peaks.

Data access links<br>*May remain private before publication.*	GSE171599
Files in database submission	HMEC-ATAC_peaks.bed, 231L_ATAC_GFP-H2A_2_4_NP.bed, 2G_K4me1-12_hg19_NP.bed, DF-omniATAC_peaks.bed, HMEC-1-ATAC.bw, HMEC_m1_fold.bigWig, HMEC_m2_fold.bigWig, MCF7_mH2A1_FE.bw, MCF7_mH2A2_FE.bw, Mel_m1_fold.bigWig, Mel_m2_fold.bigWig, 231L_ATAC_GFP-H2A_2_4.bw, 231L_ATAC_mH2A2_2_5.bw. 231L_m1_hg19.bw, 231L_m2_hg19.bw, 231L_H3K27me3_hg19.bw, 2M_m2_hg19.bw ,2G_K27Ac-2_hg19_Norm.bw, 2G_K4me1-12_hg19_Norm.bw, 2G_p300-16_hg19_Norm.bw, 2G_Brd4-6_hg19_Norm.bw, 2M_K27Ac-1_hg19_Norm.bw, 2M_K4me1-11_hg19_Norm.bw, 2M_p300-15_hg19_Norm.bw, 2M_Brd4-5_hg19_Norm.bw, DF-omniATAC-mm9.bw, HMEC_Input_ATCACG_L002_R1_001.fastq.gz, HMEC_m1_CGATGT_L003_R1_001.fastq.gz, HMEC_m2_ATCACG_L003_R1_001.fastq.gz, Input.fastq.gz, mH2A1.fastq.gz, mH2A2.fastq.gz, 0_5_E2_S1_L002_I1_001.fastq.gz, 0_5_E2_S1_L002_R1_001.fastq.gz, 0_5_E2_S1_L002_R2_001.fastq.gz, 0_5_E2_S1_L002_R3_001.fastq.gz, 2_1_E2_S4_L002_I1_001.fastq.gz, 2_1_E2_S4_L002_R1_001.fastq.gz, 2_1_E2_S4_L002_R2_001.fastq.gz, 2_1_E2_S4_L002_R3_001.fastq.gz, NHM_Input_R1.fastq.gz, NHM_mH2A1_R1.fastq.gz, NHM_mH2A2_R1.fastq.gz, 2G_ATAC.R1.fastq.gz, 2G_ATAC.R2.fastq.gz, 2M_ATAC.R1.fastq.gz, 2M_ATAC.R2.fastq.gz, 231LG_Input.fastq.gz, 231LG_mH2A1.fastq.gz, 231LG_mH2A2.fastq.gz, 231LG_H3K27me3.fastq.gz, 231LM_mH2A2.fastq.gz, 2G_Input-10.R1.fq.gz, 2G_Input-10.R2.fq.gz, 2G_K27Ac-2.R1.fq.gz, 2G_K27Ac-2.R2.fq.gz, 2G_K4me1-12.R1.fq.gz, 2G_K4me1-12.R2.fq.gz, 2G_p300-16.R1.fq.gz, 2G_p300-16.R2.fq.gz, 2G_Brd4-6.R1.fq.gz, 2G_Brd4-6.R2.fq.gz, 2M_K27Ac-1.R1.fq.gz, 2M_K27Ac-1.R2.fq.gz, 2M_K4me1-11.R1.fq.gz, 2M_K4me1-11.R2.fq.gz, 2M_p300-15.R1.fq.gz, 2M_p300-15.R2.fq.gz, 2M_Brd4-5.R1.fq.gz, 2M_Brd4-5.R2.fq.gz, mDF-omniATAC-5.FCHTYGHBBXX_L8_R1_IGGACTCCT.fastq.gz, mDF-omniATAC-5.FCHTYGHBBXX_L8_R2_IGGACTCCT.fastq.gz, HMEC_ATAC_R1.fastq.gz, MCF7_0_5_E2_scATAC_S4_L008_I1_001.fastq.gz, 0_5_E2_S1_L002_I1_001.fastq.gz MCF7_2_1_E2_scATAC_S1_L008_I1_001.fastq.gz, 2_1_E2_S4_L002_I1_001.fastq.gz, 1_dKO2_d3_S1_Lall_R1_001.fastq.gz,2_dKO2_d3_S2_Lall_R1_001.fastq.gz,D92-GEX_S3_L003_I1_001.fastq.gz,D92-ATAC_S2_L007_I1_001.fastq.gz,D92-2-GEX_S1_L004_I1_001.fastq.gz, D92-2-ATAC_S4_L008_I1_001.fastq.gz,D93-GEX_S2_L004_I1_001.fastq.gz, D93-ATAC_S1_L007_I1_001.fastq.gz ,D93-2-GEX_S4_L003_I1_001.fastq.gz ,D93-2-ATAC_S3_L008_I1_001.fastq.gz
Genome browser session<br>(e.g. UCSC)	https://genome.ucsc.edu/s/wazimismail/231L_2021<br>https://genome.ucsc.edu/s/wazimismail/MCF7_2021<br>https://genome.ucsc.edu/s/wazimismail/DF_2021<br>https://genome.ucsc.edu/s/wazimismail/HMEC_2021<br>https://genome.ucsc.edu/s/wazimismail/NHM_2021

Methodology

Replicates	One replicate for all ChIP-seq experiments, significant finding as the single cell sequencing and the BRD4 loss were confirmed with another biological replicate
Sequencing depth	Experiment, Total number of reads, uniquely mapped reads, length of reads, single/paired<br>HMEC mH2A1, 67425753, 65751670, 100, single<br>HMEC mH2A2, 73639420, 72103590, 100, single<br>MCF7 mH2A1, 36055353, 35300395, 100, single<br>MCF7 mH2A2, 79506273, 77898260, 100, single<br>NHM mH2A1, 57986443, 56607553, 100, single<br>NHM mH2A2, 49187362, 47929012, 100, single<br>231L mH2A1 (GFP), 67587068, 65007058, 95, single<br>231L mH2A2 (GFP), 62589169, 59718626, 95, single<br>231L mH2A2 (m2OE), 59163724, 57994853, 95, single
Antibodies	H3K27me3, Millipore, cat# 07-449, lot# 3018864<br>H3K27ac, Abcam, cat# ab4729, lot# GR3202987-4<br>H3K27ac ,Cell signaling, cat# 8173BC, lot# D5E4<br>H3K4me1, Homemade, lot# 1<br>P300 (Clone NM11), Active Motif, cat# 61401, lot# 01813001<br>BRD4, Bethyl, cat# A301-985A50, lot# 4<br>BRD4, Abcam, catalog#ab128874, lot#GR3251918-5<br>mH2A1, Abcam, catalog# ab37264, lot# GR3219297-2<br>mH2A2, Homemade, lot#1691
Peak calling parameters	macs2 callpeak --keep-dup 2 --bw 150 --bdg --SPMR -q 1e-5 --broad --broad-cutoff 1e-2
Data quality	TrimGalore was used to ensure sequencing quality standards. Macs2 parameters -q 1e-5 and --mfold 5 (default), were used to ensure peak quality

Software | The following software were used: TrimGalore v0.6.4, Cutadapt v3.1, bowtie2 v2.3.3.1, samtools v1.9, Picard v2.9.0, macs2 v2.2.7.1

Flow Cytometry

Plots

Confirm that:

- [ ] The axis labels state the marker and fluorochrome used (e.g. CD4-FITC).
- [ ] The axis scales are clearly visible. Include numbers along axes only for bottom left plot of group (a 'group' is an analysis of identical markers).
- [ ] All plots are contour plots with outliers or pseudocolor plots.
- [x] A numerical value for number of cells or percentage (with statistics) is provided.

Methodology

Sample preparation	NG4 embryonic stem cells and mammary epithelial cells were trypsinized, washed with PBS and strained before analyzed.
Instrument	LSRII
Software	BD FACS Diva, FlowJo
Cell population abundance	Proportions of positive and negative cells are described in the plots.
Gating strategy	GFP + and GFP- cells were identified for NG4. Red blood cell lysis buffer and EasyStep Mouse Epithelial Cell Enrichment kit (Stem Cell) was used to negatively select blood cells. Various MEC subpopulations were FACS sorted using specific cell lineage markers (EpCAM, CD49b, CD49f, Sca1)

- [x] Tick this box to confirm that a figure exemplifying the gating strategy is provided in the Supplementary Information.
